# Improved Snake Optimization Algorithm for Global Optimization and Engineering Applications

**DOI:** 10.1038/s41598-025-01299-2

**Published:** 2025-05-25

**Authors:** Kaiyuan Zheng, Huiyong Liu, Bopeng Li

**Affiliations:** College of Computer Science, Beijing Information Science and Technology University, No. 55 Taihang Road, Changping District, 102206 Beijing China

**Keywords:** Metaheuristic algorithm, Snake optimization algorithm, RIME algorithm, Global optimization, Engineering applications, Mathematics and computing, Computer science

## Abstract

In engineering applications, many complex problems can be formulated as mathematical optimization challenges, and efficiently solving these problems is critical. Metaheuristic algorithms have proven highly effective in addressing a wide range of engineering issues. The Snake Optimization Algorithm (SO) is a novel metaheuristic method with widespread use. However, SO has limitations, including reduced search efficiency in later stages and a tendency to get trapped in local optima, preventing full exploration of the solution space. To overcome these, this paper introduces the Multi-strategy Improved Snake Optimization Algorithm (ISO), which integrates six key strategies. First, the Sobol sequence is used for population initialization, ensuring uniform distribution and enhancing global exploration. Second, the RIME algorithm accelerates convergence and improves exploitation. Lens reverse learning further promotes exploration, avoiding local optima. Levy flight facilitates large random steps, balancing exploration and refinement. Adaptive step-size adjustment dynamically tunes the step size based on fitness, optimizing exploration-exploitation. Lastly, the Brownian random walk introduces local perturbations to fine-tune solutions. These strategies collectively improve convergence speed, stability, and optimization capability, ensuring an effective balance between exploration and exploitation. The ISO population distribution was evaluated using three uniformity algorithms: Average Nearest Neighbor Distance, Star Discrepancy, and Sum of Squared Deviations (SSD). ISO demonstrated improvements of 63.08%, 26.09%, and 8.88%, respectively, over SO. Its exploration-exploitation balance and convergence were analyzed on the 30-dimensional CEC-2017 benchmark functions. Additionally, ISO was tested on 23 classic benchmark functions, CEC-2011, and CEC-2017 benchmark functions. Results showed ISO’s superior performance in convergence speed, stability, and global optimization. Furthermore, ISO was successfully applied in four engineering domains: UAV path planning, robot path planning, wireless sensor network node deployment, and pressure vessel design. In all cases, ISO outperformed SO with rapid convergence and strong robustness, achieving performance improvements of 5.69%, 34.61%, 20.73%, and 7.8%, respectively, underscoring its superior efficacy in practical applications.

## Introduction

With the rapid advancement of society, academic research and engineering applications are increasingly confronted with complex optimization problems^1^. Optimization algorithms are typically categorized into two main types: deterministic algorithms and non-deterministic algorithms^2^. A deterministic algorithm consistently produces the same output for a given input and rigorously seeks the exact solution to the problem. While such algorithms often yield optimal solutions, their computational demands are high, particularly when applied to large datasets, and they tend to require longer execution times. Furthermore, in complex high-dimensional problems, deterministic algorithms are prone to becoming trapped in local optima, thereby exhibiting limited adaptability. In light of an increasingly complex and diverse problem space, there is a pressing need for more efficient, stable, and highly portable algorithms to overcome these challenges. The metaheuristic algorithm, proposed in 1986, is also known as the universal heuristic algorithm or the universal heuristic algorithm. In computer science and mathematical optimization, meta heuristic algorithms can provide a sufficiently good solution for an optimization problem. The non derivative or non gradient property is an important property of meta heuristic algorithms, which allows these algorithms to be quickly applied to optimization problems in various complex scenarios without needing to pay attention to the specific structure of the problem They can achieve near-optimal solutions while reducing computational resource requirements, making them suitable for complex real-world optimization issues. These algorithms simulate natural processes such as collective behavior, genetics, evolution, and simulated annealing, demonstrating strong robustness across diverse problems and widespread practical applications^3^. Despite significant advancements in various fields, metaheuristic algorithms also encounter challenges and issues^4^, including vulnerability to local minima, inadequate stability, and slow rates of convergence. Notably, the ‘No Free Lunch Theorem’ (NFL Theorem)^5^ explicitly states that no single algorithm can outperform all others across all possible optimization problems. In other words, an optimization algorithm may excel on certain problems but perform poorly on others. This compels researchers to design efficient algorithms tailored to specific optimization problems, aiming to provide superior solutions.

Metaheuristic optimization algorithms are a class of techniques designed to solve complex optimization problems that are often difficult or infeasible to tackle with traditional optimization methods. These algorithms are characterized by their ability to explore vast solution spaces and provide good solutions to problems with many variables, constraints, and nonlinearities. They combine exploration (searching new areas of the solution space) with exploitation (refining existing solutions), allowing them to find near-optimal solutions in large, complex, and often poorly understood search spaces.

In recent years, a variety of novel metaheuristic algorithms have been developed, each showcasing unique capabilities for addressing complex optimization problems. These include the Potter Optimization Algorithm (POA)^6^, Cave Weaving Optimization (CWO)^7^, Fossa Optimization Algorithm (FOA)^8^, Addax Optimization Algorithm (AOA)^9^, Dollmaker Optimization Algorithm (DOA)^10^, and Sculptor Optimization Algorithm (SOA)^11^. Each of these algorithms introduces distinct strategies that enhance the efficiency and versatility of the search process, broadening the range of applications in optimization tasks. For instance, the Potter Optimization Algorithm (POA) is inspired by the pottery-making process, where a potter molds clay to achieve a desired shape. This algorithm consists of a dual-phase structure: the exploration phase, which corresponds to the rough shaping of clay, and the exploitation phase, which focuses on refining and detailing the form. This division allows POA to effectively balance global exploration with local refinement, making it particularly well-suited for complex optimization problems that require both an extensive search and precise optimization. Similarly, the Cave Weaving Optimization (CWO) algorithm draws inspiration from organisms weaving structures in caves, adapting to changing environments. CWO models this behavior by incorporating two distinct phases: the exploration phase, which makes large adjustments in the solution space, and the exploitation phase, which focuses on fine-tuning and detailed adjustments. This dual-phase approach is highly effective for problems with dynamic or complex constraints, enabling CWO to excel in scenarios involving multiple optima or evolving objective functions. The Fossa Optimization Algorithm (FOA), inspired by the intelligent hunting strategy of the fossa, a predator native to Madagascar, introduces an innovative approach that balances exploration and exploitation. FOA mimics the fossa’s two-stage hunting process: the exploration phase represents the fossa’s broad search for prey, and the exploitation phase reflects the fossa’s pursuit of the prey through trees. This structure ensures that FOA performs a global search while refining solutions in promising areas, making it particularly effective in tackling complex, multimodal optimization problems that require a balance between wide-ranging exploration and focused exploitation. The Addax Optimization Algorithm (AOA), on the other hand, takes its inspiration from the foraging and digging behavior of the addax, a desert-dwelling antelope. AOA operates in two stages, with the exploration phase representing a broad search for resources (foraging) and the exploitation phase corresponding to more refined movements (digging). This dual-phase structure ensures that AOA can address both global search and local optimization, making it particularly versatile for large-scale, complex optimization problems in fields such as engineering and resource management. Meanwhile, the Dollmaker Optimization Algorithm (DOA) is inspired by the intricate process of doll making, where a doll is assembled and refined over multiple stages. In DOA, the exploration phase corresponds to the initial assembly of the doll, while the exploitation phase focuses on the meticulous refinement of details, such as facial features and finishing touches. This approach allows DOA to perform efficient exploration and exploitation, providing strong results across various benchmark functions. Finally, the Sculptor Optimization Algorithm (SOA) takes its inspiration from the sculpting process, where a sculptor makes both large and small modifications to the material to achieve the final form. SOA’s exploration phase simulates the rough cutting of a sculpture, making broad modifications to the material, while the exploitation phase focuses on more precise, detailed adjustments. This balanced approach between exploration and exploitation allows SOA to effectively solve both unimodal and multimodal optimization problems, as demonstrated by its performance on the CEC 2017 test suite.

However, in real-world scenarios, noise manifests in various forms, such as errors, model uncertainties, hardware malfunctions, and operational noise from devices. In the presence of noisy disturbances, algorithms may encounter challenges, making the exploration of optimization methods in noisy environments an urgent task. To mitigate the impact of noise when solving non-convex optimization problems, a coevolutionary neural network (CNS) consisting of a simplified neural dynamics (SND) model and a particle swarm optimization (PSO) algorithm has been proposed. The CNS method inherits the strengths of both the SND model and the PSO algorithm, striking an effective balance between rapid convergence and noise robustness. Compared to existing meta-heuristic algorithms, the CNS outperforms other methods in solving non-convex optimization problems. Moreover, the integration of the PSO algorithm significantly enhances the global convergence of the SND model, enabling it to effectively handle non-convex optimization tasks while improving the robustness of the optimization process.^12^ The Snake Optimization Algorithm (SO)^13^ draws inspiration from the behavioral patterns of snakes. While it has certain advantages in engineering problems^14^, it also suffers from limitations such as uneven population distribution and a tendency to converge prematurely to local optima. To address these challenges, we have systematically improved the algorithm, focusing on enhancing convergence speed, optimization accuracy, and effectively avoiding local optima. By employing multiple strategies to optimize SO, the proposed Improved Snake Optimization Algorithm (ISO) demonstrates excellent performance in solution quality, convergence speed, and stability.

The main contributions of this paper are as follows:

1. Developing a mathematical model to characterize and evaluate each phase of ISO.
2. Evaluating ISO’s effectiveness and robustness using a test suite of 23 tasks, CEC-2011 benchimark functions, and CEC-2017 benchmark functions, encompassing single-peak, multi-peak, hybrid, composite tasks, and real-world problems.
3. Testing ISO’s performance for solving practical optimization problems in UAV route planning, robotic trajectory planning, wireless network configuration, and pressure vessel design. These enhancements indicate that ISO can identify superior solutions more swiftly than comparative algorithms, demonstrating considerable potential in real-world applications. This finding is of profound importance for the advancement of the optimization domain.

The structure of this article is as follows: Section 2 is a literature review, reviewing and discussing past meta heuristic algorithms. The third and fourth chapters are the construction of the theoretical framework, with the third chapter explaining the original SO and the fourth chapter proposing three improvement strategies, including the fusion of RIME algorithm. Section 5 provides an in-depth analysis of the algorithmic complexity of ISO, including both time complexity and space complexity. Section 6 quantitatively calculates the population distribution of the ISO algorithm in space using three algorithms: Average Nearest Neighbor Distance, Sum of Squared Deviations (SSD), and Star Discrepancy, and uses CEC-2017 to determine the balance between exploration and development of ISO. Section 7 discusses the proposed ISO algorithm’s specific solution approach for solving constrained optimization problems. Section 8 employs a comprehensive and rigorous evaluation of the ISO algorithm alongside six competitive algorithms, utilizing the most classic and widely used 23 benchmark functions, the CEC-2011 benchmark functions, and the 30-dimensional, 50-dimensional, and 100-dimensional CEC-2017 benchmark functions. This thorough assessment aims to evaluate the performance of the ISO algorithm in solving optimization problems across various scenarios. Section 9 applies the algorithm to four types of engineering scenarios and compares and analyzes the results with six competing algorithms. Section 10 summarizes the entire work, analyzes the potential limitations of the ISO algorithm, and outlines future research directions.

## Literature review

Metaheuristic algorithms primarily encompass Evolutionary Algorithms (EA)^15^, Physics-inspired Algorithms (PhA)^16^, Human Behavior-Based Algorithms (HBA)^17^, and Swarm Intelligence Algorithms (SIA)^18^. Evolutionary Algorithms (EA) are optimization techniques grounded in the principles of natural selection and genetics. The fundamental concept is to emulate the evolutionary process of organisms through mechanisms such as selection, crossover, and mutation, thereby continually enhancing the quality of solutions. Genetic Algorithms (GA)^19^ represent a category of optimization algorithms derived from natural selection and genetic principles, utilized to address complex optimization challenges. Genetic Programming is a methodology for the automatic generation and selection of computer programs, inspired by the process of biological evolution, to fulfill user-specific tasks. Differential Evolution Algorithms (DEA)^20^ attain optimal solutions by discarding subpar individuals and preserving superior ones.Cultural Algorithms (CA)^21^ employ an additional domain termed the “belief space” to aggregate and exploit pertinent information about the behavior of individuals within the search space. Since their inception, Cultural Algorithms have been effectively extended and applied to resolve a variety of problems across different technological fields.

PHA draw inspiration from physical phenomena such as gravity, electromagnetism, and quantum mechanics. These algorithms emulate physical processes to determine ideal resolutions to intricate issues. Simulated Annealing (SA)^22^ is modeled after the metallurgical technique of heating a material to its liquefaction point and then incrementally cooling it to create a highly structured solid form. By systematically decreasing the system’s “temperature,” SA aims to find the global optimal solution to the problem. Particle Swarm Optimization (PSO)^23^ aims to achieve the optimal resolution by replicating the behavior of particles within the search space. In this method, each particle symbolizes a possible solution and continuously refines and enhances its position by learning from the collective experiences of the swarm. Quantum-inspired Evolutionary Algorithms (QIEA)^24^ draw from the advancements in quantum mechanics, employing concepts such as quantum bits and superposition states for optimization computations. This algorithm seeks the optimal solution through the progression of quantum states, thereby decreasing the time required for large-scale data processing and enhancing efficiency.

HBBA focus on improving solution quality by mimicking human learning and decision-making processes. These algorithms optimize by emulating human expert behaviors, decision tree structures, reinforcement learning, and case-based reasoning. The Case-Based Reasoning Algorithm (CBRA)^25^ addresses current problems by recalling and applying successful past cases. This algorithm emulates the human problem-solving approach through experience and is utilized in fields like medical diagnosis, legal judgment, and fault diagnosis.The Reinforcement Learning Algorithm (RLA)^26^ learns optimal strategies through interaction with the environment. Using reward and punishment mechanisms to mimic human trial-and-error learning, reinforcement learning algorithms continuously optimize behavior strategies and are widely applied in AI, robot control, and financial trading. The Draco Lizard Optimizer (DLO) is inspired by the Draco lizard’s gliding and camouflage behaviors. The DLO employs a two-phase process-global exploration followed by local refinement-to efficiently solve global optimization problems with high computational performance .^27^ The Frigatebird Optimizer (FBO) is inspired by the foraging and flight behaviors of frigatebirds. It uses a two-stage process: one for global search and another for fine-tuned local optimization. FBO performs well in optimization tasks, particularly in global exploration and local exploitation.^28^ The Fishing Cat Optimizer (FCO) is inspired by the fishing cat’s hunting techniques, such as ambush, detection, diving, and trapping. The algorithm is structured into four optimization phases, facilitating both effective global exploration and precise local refinement. In benchmark tests, the FCO has consistently outperformed various other algorithms, showcasing its ability to tackle complex optimization challenges.^29^

Swarm Intelligence Algorithms (SIA) are computational approaches specifically designed to address complex optimization problems by emulating the collective behaviors observed in nature. These algorithms draw inspiration from the interactions within biological groups such as ants, bees, birds, and fish. By leveraging simple, decentralized interactions among individual agents, SIA can produce sophisticated, intelligent collective behaviors that are highly effective in solving intricate optimization challenges. Ant Colony Optimization (ACO)^30^ is modeled after the foraging patterns of ants. It seeks optimal solutions, particularly in path optimization tasks, by replicating the movements of virtual ants within the search space and updating pheromone trails. The Salp Swarm Algorithm (SSA)^31^ draws its conceptual framework from the swarming behavior of salp chains. SSA has proven to be highly effective in optimizing various engineering design problems. The Eurasian Lynx Optimizer (ELO) is inspired by the Eurasian lynx’s hunting and survival strategies. It divides the optimization process into three phases: exploration, exploration & exploitation, and exploitation, with each phase using different search methods.^32^ The algorithm outperforms many other algorithms in global optimization problems, making it suitable for engineering applications . The Artificial Meerkat Algorithm (AMA) mimics the cooperative and competitive behaviors of meerkats in nature. It utilizes multiple search strategies and a multi-stage process to solve complex optimization problems. The algorithm has been shown to outperform several other algorithms in benchmark tests.^33^

Since the invention of SO , numerous researchers have enhanced this algorithm and applied the improved versions to engineering fields. Karam Khairullah Mohammed and colleagues optimized the SO for fast and precise maximum power tracking in photovoltaic systems. Chaohua Yan and Navid Razmjooy crafted an advanced version of the Snake Optimization Algorithm and applied it with Convolutional Neural Networks for nodule prediction on the “IQ-OTH/NCCD-Lung Cancer Dataset.t^34^. Weimin Zheng and his team introduced a compact strategy in the SO for population probability modeling, improving indoor positioning accuracy^35^. Yang Rui and colleagues proposed an efficient enhanced Snake Optimizer ,and benchmarked its performance against several algorithms in terms of benchmark functions and engineering design applications^36^. Building on the steps of the Snake Optimization Algorithm and previous improvement strategies,This article integrates three advanced strategies into the Snake Optimization (SO) algorithm. Firstly, Sobol sequence initialization^37^ enhances exploration by distributing initial points more uniformly across the search space, utilizing low-discrepancy sequences for broader coverage. RIME algorithm’s^38^ core formula, inspired by the physical rime ice formation, optimizes exploration and exploitation balance, allowing the algorithm to adaptively search complex, high-dimensional spaces.The Lens Imaging Reverse Learning strategy^39^ simulates lens imaging to enhance traversal and avoid local optima by reverse learning, significantly boosting convergence performance. Lastly, the incorporation of Lévy flight introduces a stochastic process with long-range jumps that prevent premature convergence by enabling the algorithm to escape local optima while maintaining a balance between local exploitation and global exploration. The improved algorithm is applied to 6 engineering questions, and comparative experiments have been conducted to evaluate its performance.

## Snake optimization algorithm

### Initialization

The original Snake optimization begins by generating a random population with a uniform distribution to initiate the optimization process. The initial population can be determined using the following equation:

1 $$\begin{aligned} X_i = X_{min} + r \times (X_{max} - X_{min}) \end{aligned}$$

In this context, $X_i$ denotes the position of the $i$-th individual, $r$ is a randomly generated number within the range [0, 1], and $X_{min}$ and $X_{max}$ represent the minimum and maximum bounds of the problem, respectively.

### Dividing the swarm into two equal groups: males and females

SO will set up a simplified gender distribution scenario. Assuming a balanced population composition consisting of 50% males and 50% females. To analyze this binary structure, we further divided the population into two distinct groups: the male group and the female group. It is worth noting that this classification mechanism is similar to the organizational structure of bee colonies in nature. Although there are significant differences in essence, in this context, we adopted an analogy approach to specifically define and distinguish these two gender groups through formulas (2) and (3) for subsequent data processing and analysis.

2 $$\begin{aligned} & N_m \approx N / 2 \end{aligned}$$

3 $$\begin{aligned} & N_f = N - N_m \end{aligned}$$

Where *N*, $N_m$, $N_f$ are the total number of individuals, the number of males, and the number of females, respectively.

### Evaluate each group and define temperature and food quantity

In order to optimize the selection process, we identified the best performing males from the male group, labeled as $(f_{\text {best}_m})$, while selecting the best performing females from the female group, labeled as $(f_{\text {best}_f})$. In addition, we also focused on the importance of food location $(f_{\text {food}})$ as one of the key factors affecting survival and reproduction.

- Temperature (*Temp*) can be expressed as:
  4 $$\begin{aligned} Temp = \exp \left( -\frac{t}{T}\right) \end{aligned}$$
  *t*, *T* represents the current and maximum iteration times, respectively.
- The definition of quantity (*Q*) is:
  5 $$\begin{aligned} Q = c_1 \times \exp \left( -\frac{t-T}{T}\right) \end{aligned}$$
  Where $c_1$ is a constant equal to 0.5.

### Exploration phase (no food)

If $Q < \text {Threshold}$ (Threshold = 0.25), the snakes search for food by selecting random positions and updating their positions accordingly. To model the exploration phase, the following equation is used:

6 $$\begin{aligned} X_{i,m}(t+1) = X_{rand,m}(t) \pm c_2 \times A_m \times ((X_{max} - X_{min}) \times rand + X_{min}) \end{aligned}$$

Here, $X_{i,m}$ denotes the position of the male, $X_{rand,m}$ indicates the position of a randomly selected male, $rand$ is a random number between 0 and 1, and $A_m$ represents the male’s ability to find food, calculated as follows:

7 $$\begin{aligned} A_m = \exp \left( -\frac{f_{rand,m}}{f_{i,m}}\right) \end{aligned}$$

In this description, $f_{\text {rand}_m}$ represents the fitness value of a randomly selected male individual $X_{\text {rand}}$, while $f_{im}$ specifically refers to the fitness value of the *i*-th individual in the male population. To evaluate the performance of randomly selected individuals relative to other individuals in their group (i.e., male group), we introduced a comparison mechanism. Among them, $c_2$ is set as a preset constant with a value of 0.05, which plays a regulatory role in the comparison process and may be used to control the selection pressure or adjust the sensitivity of fitness evaluation.

8 $$\begin{aligned} X_{i,f}(t+1) = X_{rand,f}(t) \pm c_2 \times A_f \times ((X_{max} - X_{min}) \times rand + X_{min}) \end{aligned}$$

We defined $X_{i,f}$ as specific location identifiers for each member in the female population. For random selection or comparison, we introduce $X_{\text {rand}, f}$ which represents the position of a female individual selected through a random process involving a random number *rand* between 0 and 1. In addition, the ability of female populations to search for food is quantified by the parameter $A_f$, which has been calculated based on specific attributes or behaviors of female individuals:

9 $$\begin{aligned} A_f = \exp \left( -\frac{f_{rand,f}}{f_{i,f}}\right) \end{aligned}$$

We defined $f_{rand,f}$ is the fitness of $X_{rand,f}$, and $f_{i,f}$ is the fitness of the ith individual in the female group.

### Exploitation phase (food exists)

If $$Q > \text {Threshold}$$:

- If the temperature $> \text {Threshold}$ (0.6) (hot):The snakes will move to the food only:
  10 $$\begin{aligned} X_{i,j}(t+1) = X_{food} \pm c_3 \times Temp \times rand \times (X_{food} - X_{i,j}(t)) \end{aligned}$$
  Where $X_{i,j}$ represents the position of the individual (male or female), $X_{food}$ is the position of the best individuals, and $c_3$ is a constant equal to 2.
- If the temperature $< \text {Threshold}$ (0.6) (cold):
  The snake will be in either fight mode or mating mode.

- Fight Mode:
  11 $$\begin{aligned} X_{i,m}(t+1) = X_{i,m}(t) + c_3 \times FM \times rand \times (Q \times X_{best,f} - X_{i,m}(t)) \end{aligned}$$
  Among them, $X_{i,m}$, $X_{\text {best}, f}$ are the *i* and best male positions respectively, and *FE* is the combat ability of female agents. *FM* is the fighting capabilities of the male agents:
  12 $$\begin{aligned} X_{i,f}(t+1) = X_{i,f}(t) + c_3 \times FF \times rand \times (Q \times X_{best,m} - X_{i,f}(t+1)) \end{aligned}$$
  Among them, $X_{i,f}$, $X_{\text {best}, m}$ are the *i* and best female positions respectively, and *FF* is the combat ability of female agents. The *FM* and *FF* can be expressed as:
  13 $$\begin{aligned} FM= & \exp \left( -\frac{f_{best,f}}{f_i}\right) \end{aligned}$$
  14 $$\begin{aligned} FF= & \exp \left( -\frac{f_{best,m}}{f_i}\right) \end{aligned}$$
  Where $f_{best,f}$ is the fitness of the best agent in the female group, $f_{best,m}$ is the fitness of the best agent in the male group, and $f_i$ is the fitness of the agent.

- Mating Mode:
  15 $$\begin{aligned} X_{i,m}(t+1)= & X_{i,m}(t) + c_3 \times M_m \times rand \times (Q \times X_{i,f}(t) - X_{i,m}(t)) \end{aligned}$$
  16 $$\begin{aligned} X_{i,f}(t+1)= & X_{i,f}(t) + c_3 \times M_f \times rand \times (Q \times X_{i,m}(t) - X_{i,f}(t)) \end{aligned}$$
  Where $X_{i,f}$ is the position of the ith agent in the female group, and $X_{i,m}$ is the position of the ith agent in the male group. $M_m$ and $M_f$ refer to the mating ability of males and females, respectively, and can be calculated as follows:
  17 $$\begin{aligned} M_m= & \exp \left( -\frac{f_{i,f}}{f_{i,m}}\right) \end{aligned}$$
  18 $$\begin{aligned} M_f= & \exp \left( -\frac{f_{i,m}}{f_{i,f}}\right) \end{aligned}$$

If an egg hatches, select the worst male and female and replace them:

19 $$\begin{aligned} X_{worst,m}= & X_{min} + rand \times (X_{max} - X_{min}) \end{aligned}$$

20 $$\begin{aligned} X_{worst,f}= & X_{min} + rand \times (X_{max} - X_{min}) \end{aligned}$$

Where $X_{worst,m}$ is the worst individual in the male group, and $X_{worst,f}$ is the worst individual in the female group.

### Termination conditions

**Fig. 1:**
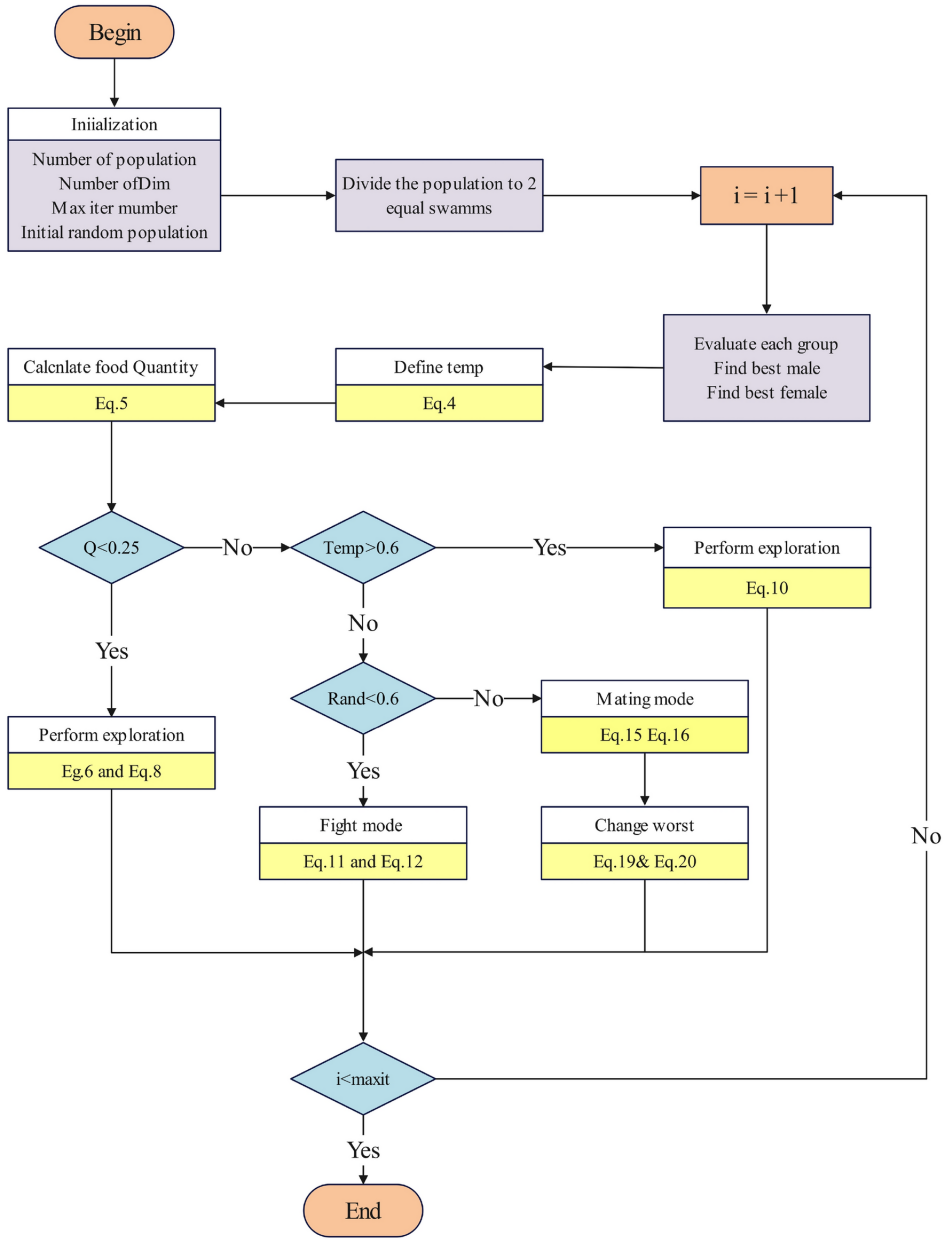
The flowchart of the SO. It outlines the process from population initialization to the application of different modes until the termination condition is met.

SO algorithm continues until the termination conditions are met. The termination conditions will be defined by a set number of iterations or a convergence criterion. The flowchart of the SO algorithm is shown in Fig. 1.

## Proposed method

### Initialization using sobol sequences

Sobol sequences,^40^ named after the Russian mathematician Ilya M. Sobol, are a type of low-discrepancy sequence used extensively in numerical simulations and optimization problems. These sequences are designed to produce points that are uniformly distributed throughout a multidimensional space, ensuring that the search space is comprehensively explored, particularly in high-dimensional problems. Unlike random sequences, Sobol sequences ensure better space-filling properties, reducing the risk of clustering or missing critical regions in the search domain.^41^ Sobol sequences belong to a family of quasi-random sequences known as *low-discrepancy sequences*. They are based on binary fractions and are generated through linear recurrence relations. For each dimension $D$, all coordinates possess linear recurrence relations. A non-negative instance $s$ is expressed in binary form as:

21 $$\begin{aligned} s = s_1 2^0 + s_2 2^1 + s_3 2^2 + \dots + s_w 2^{w-1} \end{aligned}$$

The $i$-th instance for dimension $D$ is generated using the following equation:

22 $$\begin{aligned} X_i^D = i_1 V_1^D + i_2 V_2^D + \dots + i_w V_w^D \end{aligned}$$

In (22), $v_1^D$ represents the binary function which belongs to the $d$-dimensional search space at the $i^{th}$ iteration. These direction occurrences are generated by using (23).

23 $$\begin{aligned} V_i^D = c_1 V_{i-1}^D + c_2 V_{i-2}^D + \cdots + c_q V_{q-1}^D + \left( \frac{V_i^D - q}{2q} \right) \end{aligned}$$

In (23), $c_q$ is a polynomial coefficient where $i > q$.

In the context of ISO, Sobol sequences are used to initialize the positions of the population.^42^The process of initializing the data using Sobol sequences follows these steps:

1.Generate a Sobol sequence $\{s_i\}_{i=1}^{N}$, where $s_i \in [0,1]^d$, and $d$ is the dimensionality of the problem.

2.Map the generated points to the search space defined by the lower and upper bounds, $lb$ and $ub$, respectively.

3.Assign the mapped points to the initial positions of individuals in the swarm.The position of each individual in the swarm $X_i$ is computed using the following equation:

24 $$\begin{aligned} X_i = lb + (ub - lb) \odot s_i \end{aligned}$$

where $\odot$ represents element-wise multiplication. This method ensures an even dispersion of the initial population across the search space, which sets the foundation for the exploration and exploitation phases of the optimization algorithm. Sobol sequences play a crucial role in enhancing the performance of optimization algorithms like ISO. By providing a uniform, low-discrepancy initialization in the search space, Sobol sequences improve the algorithm’s ability to explore the global optima, reduce the probability of early convergence to suboptimal solutions, and facilitate a more thorough exploration of the problem landscape. This makes Sobol sequence initialization particularly advantageous for high-dimensional optimization problems.

### Incorporating the RIME optimization algorithm

RIME^16^ is an efficient optimization algorithm based on the physical phenomenon of rime-ice. The RIME algorithm simulates the growth processes of soft-rime and hard-rime, constructing a soft-rime search strategy and a hard-rime puncture mechanism. Integrating this strategy within the ISO framework can enhance the exploration and exploitation behaviors in optimization methods, thereby improving the convergence capability of ISO. The computational method is as follows.

25 $$\begin{aligned} R_{ij}^{\text {new}} = R_{\text {best}, j} + r_1 \cdot \cos \left( \theta \right) \cdot \beta \cdot \left( h \cdot (Ub_{ij} - Lb_{ij}) + Lb_{ij}\right) \cdot r_2 < E \end{aligned}$$

Where

26 $$\begin{aligned} \theta = \pi \cdot \frac{t}{10 \cdot T}, \end{aligned}$$

And

27 $$\begin{aligned} \beta = 1 - \left( \left\lfloor \frac{W \cdot t}{T} \right\rfloor \mod W\right) . \end{aligned}$$

The integration of the RIME algorithm into the original snake optimization framework is critical^43^, necessitating a rigorous analysis of the effects on the convergence behavior and exploratory characteristics of the algorithm. The newly proposed equations (25), (26), and (27) introduce a refined mechanism for updating the position vectors. This strategy successfully balances the need for thorough exploration of the global search space with the requirement for intensive exploitation of local regions, achieved through the modulation of search amplitude by the temperature-based factor $\theta$ and the adaptive scaling factor $\beta$, both of which are influenced by the iteration count *t* and the problem’s dimensional size *W*.

### Lens imaging reverse learning

The population distributed in the function space can lead the group to find good resources due to its wide search range and flexible search methods^40^. Once some populations fall into local optima, the overall performance of the algorithm declines. Therefore, enhancing the search range of the finders is particularly important. General learning strategies have achieved good results in some optimization algorithms, but they have little impact on the algorithm’s performance. This is because general learning strategies can only perform reverse solving in local spaces, which, while enriching the population’s diversity, still have a narrow search range and lose the ability to explore the global space.^44^To improve the population’s search capability, an inverse learning strategy based on lens imaging is proposed and applied to the position update formulas of all individuals in the population to enhance the global search capability of ISO. [*lb*, *ub*] is the search space for solutions, and *y* represents the function value. Suppose there is a system $P_i$ with a height of $h_i$, and its projection at point *x* is *x*; this system is reproduced as another system $P_i^*$ on the other side with a lens focal length that is halved midway, with a height of $h_i^*$ and a projection at point $x_i$ as $x_i^*$. According to the principle of lens imaging, we obtain^45^:

28 $$\begin{aligned} \frac{\frac{lb+ub}{2} - x_i}{x_i^* - \frac{lb+ub}{2}} = \frac{h_i}{h_i^*} \end{aligned}$$

Let $k = \frac{h}{h'}$; then equation (29) can be rewritten as:

29 $$\begin{aligned} x_i^* = \frac{lb + ub}{2} + \frac{lb + ub}{2k} - \frac{x_i}{k} \end{aligned}$$

**Fig. 2:**
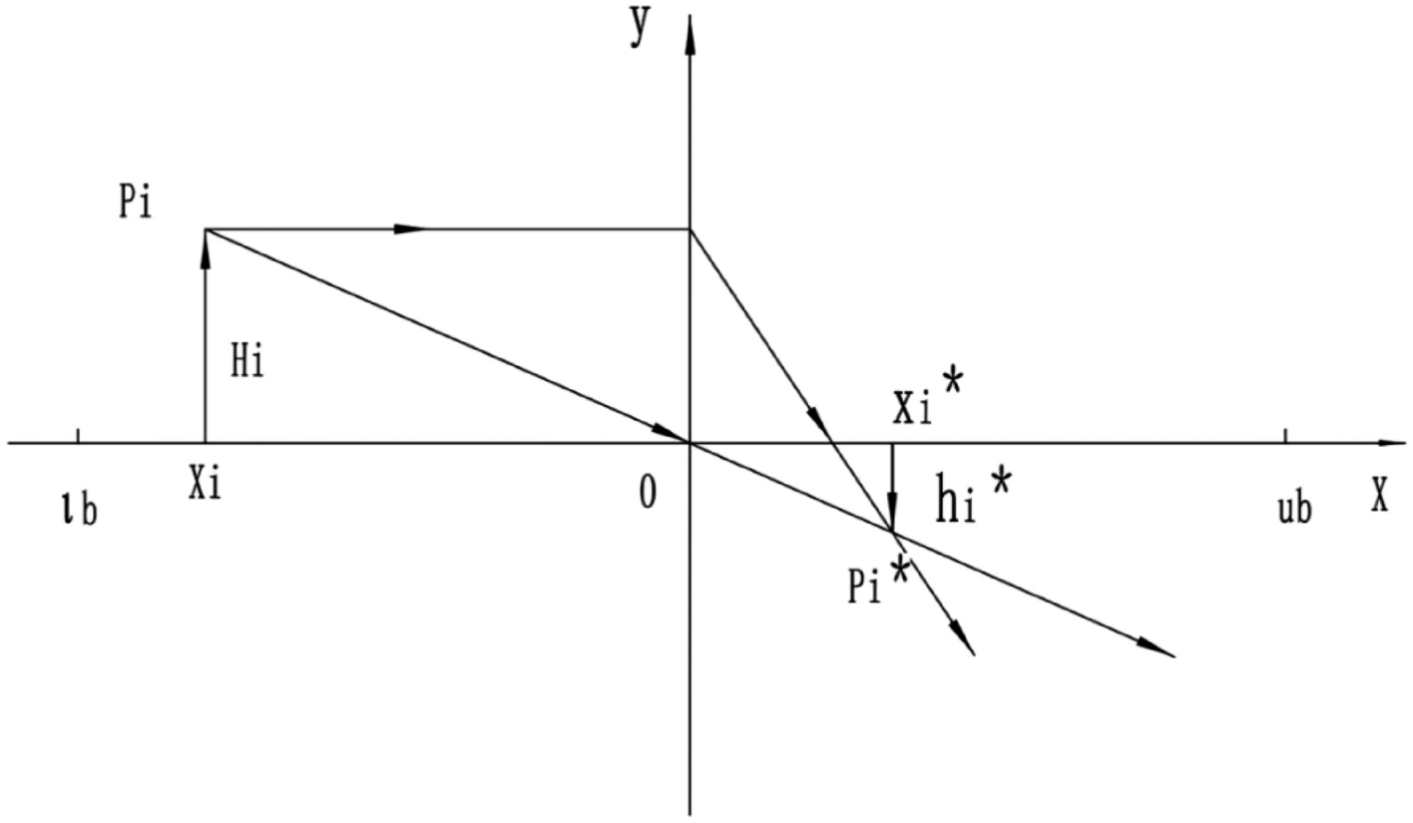
The schematic of the Lens Imaging Reverse Learning algorithm. For any population distributed within the function space, the Lens Imaging Reverse Learning algorithm can be used to obtain its inverse solution.

Equation (29) is the transformation formula for the back focal length of the original lens on the halved side. The scaling factor *k* is a freely selectable parameter that can be dynamically adjusted according to different problems. In this paper, the selection of the *k* value is based on the results of multiple experiments, which can maximize the solving ability of the ISO algorithm. As shown in Fig. 2, it illustrates the Lens Imaging Reverse Learning process.

### Lévy Flight

Lévy flight is a stochastic process characterized by a random walk where the step lengths are drawn from a Lévy distribution, known for its heavy-tailed nature. Unlike Gaussian processes, which produce steps of similar magnitude, Lévy flights exhibit a mixture of frequent small steps and rare, but significant, long jumps. This property allows algorithms using Lévy flight to maintain a balance between local search and global exploration. Mathematically, the step length $z$ of a Lévy flight is often represented as:

30 $$\begin{aligned} z = \frac{u}{|v|^{1/\beta }} \end{aligned}$$

where $u$ and $v$ are random variables following a normal distribution, and $\beta$ (commonly set between 1 and 2) controls the distribution of steps. The heavy-tailed nature of the Lévy distribution ensures that while most steps are small, occasionally large jumps are made, preventing the search process from being trapped in local optima.

This unique blend of movement patterns makes Lévy flight highly effective in global optimization tasks. The long jumps allow exploration of new regions in the search space, while the smaller steps fine-tune solutions in promising areas. This mechanism has been successfully applied in various metaheuristic algorithms, providing a more robust search capability compared to purely local search techniques. In the ISO algorithm, Lévy flight is integrated to enhance the global search capabilities, ensuring that the algorithm can escape local optima and explore the search space more efficiently.

### Adaptive step-size adjustment factor

This strategy enhances the algorithm’s global search capability through random leader selection, fitness-based guidance, and adaptive step size adjustment. Initially, a random leader is selected from the female population, and its position vector randf $ guides the update of the current individual’s position. The position update formula is as follows:

$$X_{\text {newf}}(i, j) = X_{\text {randf}}(j) + RT \times \text {Flag} \times C_2 \times A_f \times \left[ (ub(j) - lb(j)) \times \text {rand} + lb(j)\right]$$

Here, the fitness difference factor $A_f$ is defined as:

$$A_f = \exp \left( -\frac{f_{\text {rand}}}{f_i + \epsilon }\right)$$

where $f_{\text {rand}}$ represents the fitness of the randomly selected leader, and $f_i$ is the fitness of the current individual, with $\epsilon$ being a small constant to avoid division by zero. The term *RT* is an adaptive step size adjustment factor, $C_2$ is a constant, and $\text {rand}$ is a uniformly distributed random number.

This approach increases population diversity and enhances global search efficiency by combining random leadership with adaptive step size adjustments, allowing the algorithm to effectively balance exploration and exploitation.

### Brownian random walk search mechanism

The Brownian random walk search mechanism can be described as follows: the new position $X_{\text {newf}}$ of the individual is updated based on the current best solution $X_{\text {food}}$ and a random perturbation term *RB*(*i*, *j*) , which simulates small, random movements:

$$X_{\text {newf}}(i, j) = X_{\text {food}}(j) + RB(i, j) \times (\text {rand} \times X_{\text {food}}(j) - X_{\text {f}}(i, j))$$

**Fig. 3:**
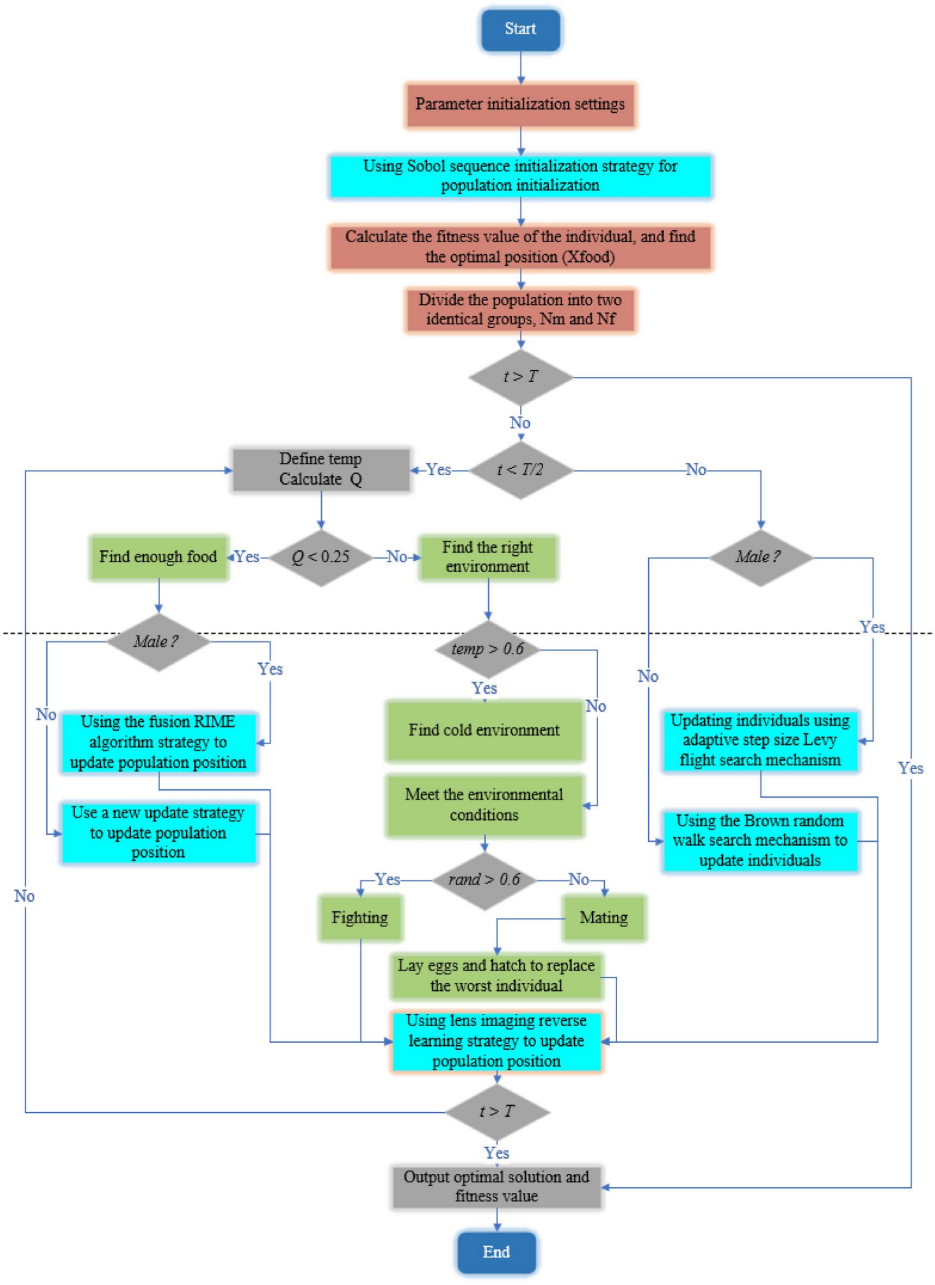
The flowchart of ISO. The diagram illustrates how six different strategies are seamlessly integrated into specific stages of the SO algorithm.

This allows the algorithm to explore the search space locally, enhancing the algorithm’s exploitation ability by mimicking the random motion found in Brownian particles. The following pseudocode outlines the specific steps of the ISO algorithm, and the flowchart of ISO is illustrated in Fig. 3.

**Algorithm 1:**
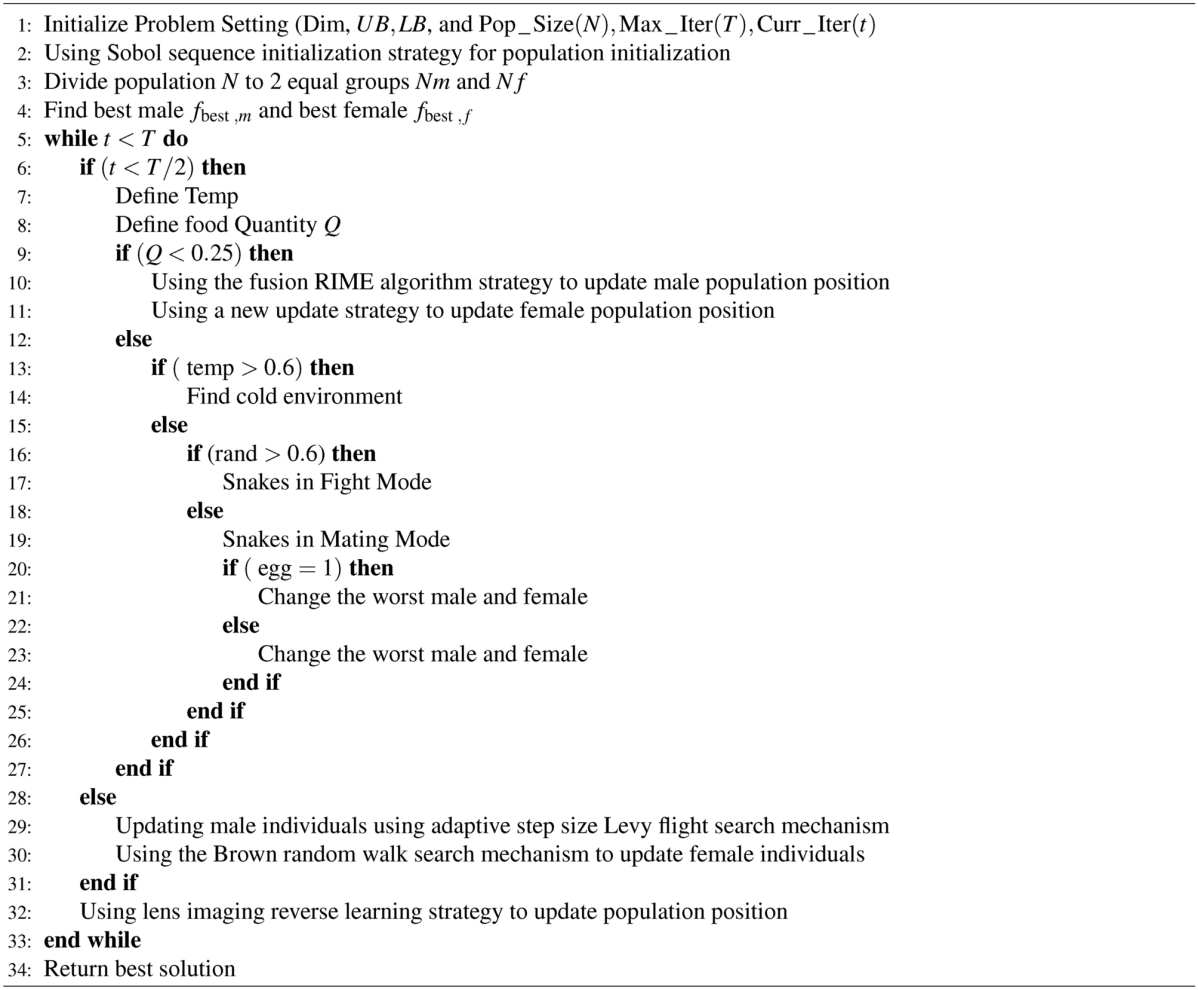
Pseudo Code of ISO

## Complexity analysis of ISO

In this section, we analyze the time complexity and space complexity of the Multi-strategy Improved Snake Optimization Algorithm (ISO). Time complexity measures the rate at which the algorithm’s execution time increases as the input size grows. It indicates the order of magnitude of the time required as the problem size expands. On the other hand, space complexity measures the rate at which the amount of memory required by the algorithm grows as the input size increases, reflecting the algorithm’s demand for memory resources. Both time complexity and space complexity are critical metrics for evaluating the efficiency of an algorithm.

### Time complexity analysis

The time complexity of the algorithm in the function ISO is $O(T \times N \times \text {dim})$, where $T$ denotes the number of iterations, $N$ represents the population size, and $\text {dim}$ is the dimensionality of the search space. The initial step, involving the generation of the Sobol sequence, has a time complexity of $O(N \times \text {dim})$, as it generates and scales the sequence for $N$ particles in a $\text {dim}$-dimensional space. The fitness evaluation for each particle takes $O(1)$ time per particle, resulting in an overall complexity of $O(N)$ for the initial fitness calculation. Within each iteration of the main optimization loop, several nested loops are executed, performing position updates, fitness evaluations, and conditional checks. Each of these operations has a time complexity of $O(N \times \text {dim})$. In particular, the “Rime fusion” and “Levy flight” steps also contribute an additional $O(N \times \text {dim})$ complexity. Finally, the global best fitness and position are updated in constant time, $O(1)$. Given that the algorithm performs these operations for $T$ iterations, the overall time complexity of the algorithm is $O(T \times N \times \text {dim})$.

### Space complexity analysis

The space complexity of the algorithm can be analyzed based on the main data structures and variables involved in storing the information. The most important variables in the algorithm are the particle position matrix $X$ and the particle fitness matrix $fitness$. The matrix $X$ has dimensions $N \times \text {dim}$, where $N$ is the number of particles and $\text {dim}$ is the dimension of the problem. This matrix stores the coordinates of each particle, so its space complexity is $O(N \times \text {dim})$. Similarly, the fitness matrix $fitness$ stores the fitness values of $N$ particles, and its space complexity is $O(N)$.In each iteration, the algorithm updates the particles’ positions, generating new position matrices $Xnewm$ and $Xnewf$, which also have dimensions $N \times \text {dim}$. Thus, the space complexity per iteration is also $O(N \times \text {dim})$. Additionally, to store subsets of the particle swarm and the best solutions, the algorithm uses temporary variables such as $Xm$, $Xf$, $fitness_m$, $fitness_f$, $Xbest_m$, and $Xbest_f$, which require space of size $O(N \times \text {dim})$ or $O(N)$, depending on the number of particles and dimensions.Other parts of the algorithm, such as the Frost-Ice algorithm, Levy flights, and lens imaging backpropagation, while updating the particle positions, only require temporary variables to store intermediate results. These operations do not contribute additional space complexity. Therefore, the extra memory required for intermediate calculations does not exceed $O(N \times \text {dim})$.

In conclusion, the space complexity of the algorithm is primarily determined by the storage requirements of the particle position matrix and the fitness matrix. The overall space complexity is $O(N \times \text {dim})$. As the number of particles $N$ and the problem dimension $\text {dim}$ increase, the memory requirement of the algorithm grows linearly.

## Population initialization and exploration-exploitation Analysis

In most cases, employing a strategy for uniform population initialization allows the initial population^46^ to be distributed across every corner of the space, providing a solid foundation for algorithm iterations. During the algorithm’s iteration process, the balance between exploration and exploitation^47^ is also a factor worth examining. The convergence behavior analysis, including search history, fitness comparisons, trajectories, and convergence curves, provides a comprehensive understanding of an algorithm’s efficiency and its ability to balance exploration and exploitation, which is crucial for evaluating its performance and suitability for solving complex optimization problems. The next three subsections provide a detailed explanation of the analysis process.

### Population initialization analysis

**Fig. 4:**
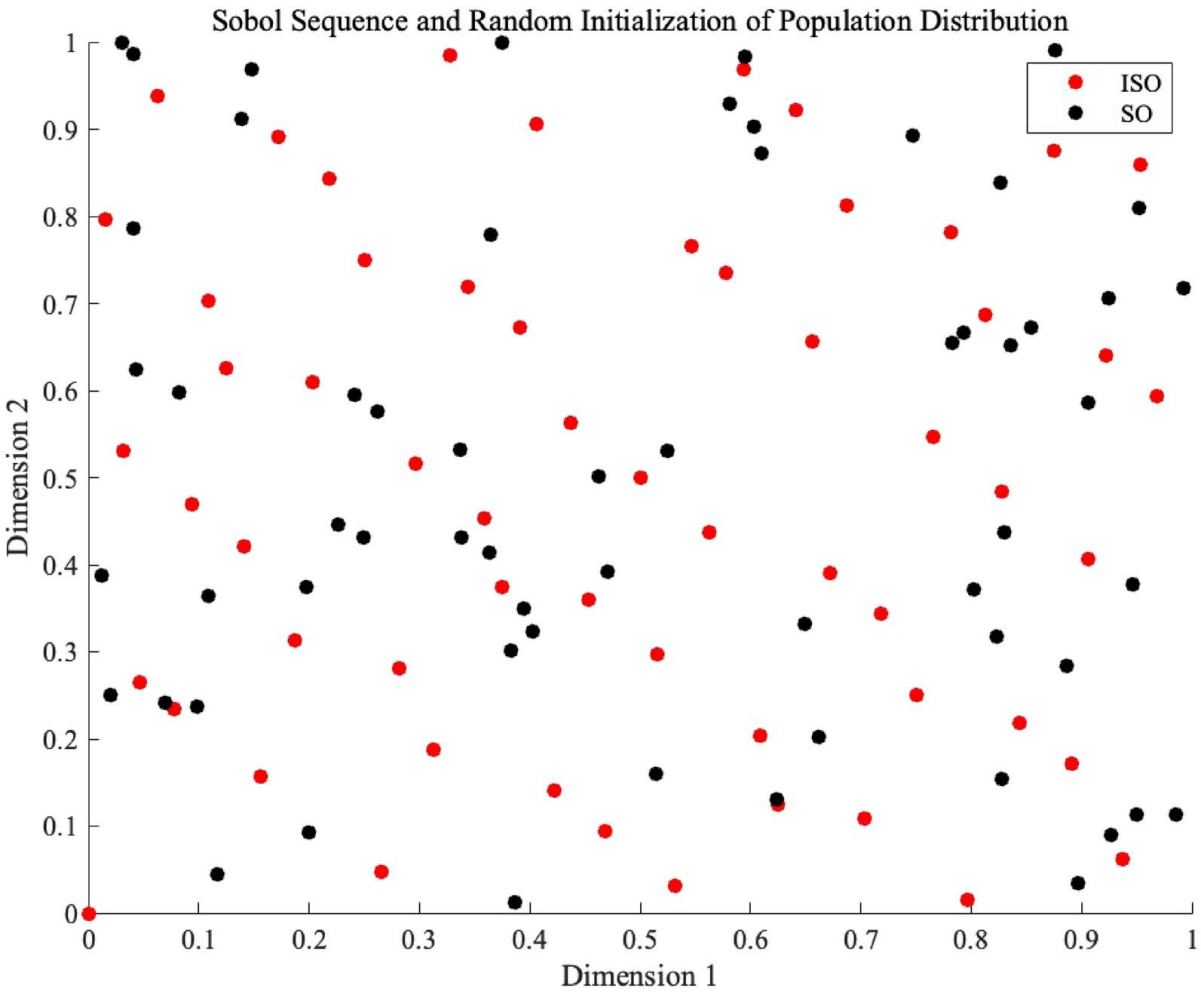
The population distribution of ISO and SO. The initial population generated by Sobol initialization in the ISO algorithm exhibits a more uniform distribution in the two-dimensional space compared to the initial population generated by random initialization in the SO algorithm.

Figure 4 shows the initialization state of the ISO population, and Table 1 presents the statistical results measured using three population uniformity evaluation algorithms: Star Discrepancy, Average Nearest Neighbor Distance, and Sum of Squared Deviations (SSD)

The three population uniformity evaluation algorithms-Star Discrepancy,Average Nearest Neighbor Distance and Sum of Squared Deviations (SSD) are crucial for evaluating the uniformity of population distributions. The specific calculations are as follows.

#### Star discrepancy

**Description:** The star discrepancy measures the overall spatial uniformity of a point set within a unit cube.^48^By calculating the maximum difference between the number of points within subrectangles and the expected number of points in a perfectly uniform distribution, the uniformity can be assessed. A smaller star discrepancy indicates a more uniform distribution, while a larger star discrepancy indicates less uniformity. **Formula:**

31 $$\begin{aligned} D^* = \max _{1 \le i \le n} \left| \frac{1}{n} \sum _{j=1}^{n} (p_j \le p_i) - \prod _{k=1}^{d} x_{ik} \right| \end{aligned}$$

where $n$ is the total number of points, $d$ is the dimension, $p_j$ is a point in the set, $p_i$ is a reference point, $x_{ik}$ is the coordinate of point $p_i$ in the $k$dimension.^49^

#### Average nearest neighbor distance

**Description:** The average nearest neighbor distance measures the uniformity of point distributions in space.^50^ By calculating the distance from each point to its nearest neighbor and taking the average of these distances, one can determine the distribution pattern. A larger average nearest neighbor distance indicates a more uniform distribution, while a smaller distance indicates clustering.^51^

**Formula:**

32 $$\begin{aligned} \text {Average Nearest Neighbor Distance} = \frac{1}{n} \sum _{i=1}^{n} \min _{j \ne i} d(p_i, p_j) \end{aligned}$$

where $n$ is the total number of points, $p_i$ and $p_j$ are points in the set, and $d(p_i, p_j)$ denotes the Euclidean distance between point $p_i$ and point $p_j$.

#### Sum of squared deviations (SSD)

**Description:** The sum of squared deviations evaluates the dispersion of a set of points relative to a central location. By calculating the squared distance from each point to the center and summing these values, one can quantify the spread of the point set. A smaller SSD indicates a more uniform distribution, while a larger SSD indicates greater dispersion.^52^

**Formula:**

33 $$\begin{aligned} \text {SSD} = \sum _{i=1}^{n} \sum _{j=1}^{d} (p_{ij} - c_j)^2 \end{aligned}$$

Where $n$ is the total number of points, $d$ is the dimension, $p_{ij}$ is the coordinate of the $i$-th point in the $j$-th dimension, and $c_j$ is the coordinate of the central point in the $j$-th dimension. Table 1 provides a detailed presentation of the population distribution results as measured by the three statistical methods. The Sobol sequence initialization (ISO) demonstrates superior uniformity compared to random initialization (SO). Specifically, ISO exhibits a significantly lower Star Discrepancy (0.047965 compared to 0.133966), showing an improvement of 63.08%. It also presents a higher Average Nearest Neighbor Distance (0.086739 compared to 0.069264), with a performance improvement of 26.09%, and a lower Sum of Squared Deviations (SSD) (9.990234 compared to 11.255603), reflecting an enhancement of 8.88%. These results collectively affirm that ISO provides a more homogeneous initial population than SO, offering a clearly more uniform initial population distribution, thereby laying a solid foundation for subsequent algorithm optimization. .^53^

**Table 1:** The results of the population distribution status as measured by the three population uniformity evaluation algorithms.

Parameter	SO (Random Initialization)	ISO (Sobol Sequence)
Star Discrepancy	0.133966	0.047965
Average Nearest Neighbor Distance	0.069264	0.086739
Sum of Squared Deviations (SSD)	11.255603	9.990234

### Exploration-exploitation analysis

The key to constructing effective metaheuristic algorithms is how to find a reasonable balance between the two. Excessive search can trap the algorithm in a large number of solution sets and prevent it from converging quickly; Moreover, excessive development can lead to algorithms easily falling into local optima, thereby losing the global optimum. So, the most ideal algorithm should be able to deeply mine it based on the search process and the quality feedback of the current solution, while ensuring the ability to search on a large scale, to achieve efficient and robust optimization effects. This dynamic strategy not only improves the algorithm’s adaptability, but also enhances its robustness, ensuring satisfactory optimization results in various situations In the context of metaheuristic algorithms, exploration and exploitation are considered two fundamental components^54^. Exploration involves investigating new solutions within the solution space, with the goal of discovering superior solutions in unexplored areas. Conversely, exploitation focuses on established solution spaces, performing searches within the local vicinities of known solutions to identify potentially better outcomes. Achieving a well-balanced combination of exploration and exploitation not only enables the algorithm to converge swiftly to optimal solutions, thereby improving search efficiency, but also provides the versatility to address a wide range of optimization challenges and complexities, demonstrating remarkable adaptability and robustness. An effective algorithm must maintain an optimal balance between these two aspects.*Div(t)* is a measure of dimension diversity calculated by Eq. (36). Here, $x_{id}$ represents the position of the $i^{th}$ dimension, and $Div_{max}$ denotes the maximum diversity throughout the entire iteration process.

34 $$\begin{aligned} Exploration(\%)= & \frac{Div(t)}{Div_{max}} \times 100 \end{aligned}$$

35 $$\begin{aligned} Exploitation(\%)= & \left| \frac{Div(t) - Div_{max}}{Div_{max}} \right| \times 100 \end{aligned}$$

36 $$\begin{aligned} Div(t)= & \frac{1}{D} \sum _{d=1}^{D} \frac{1}{N} \sum _{i=1}^{N} \left| median(x_d(t)) - x_{id}(t) \right| \end{aligned}$$

**Fig. 5:**
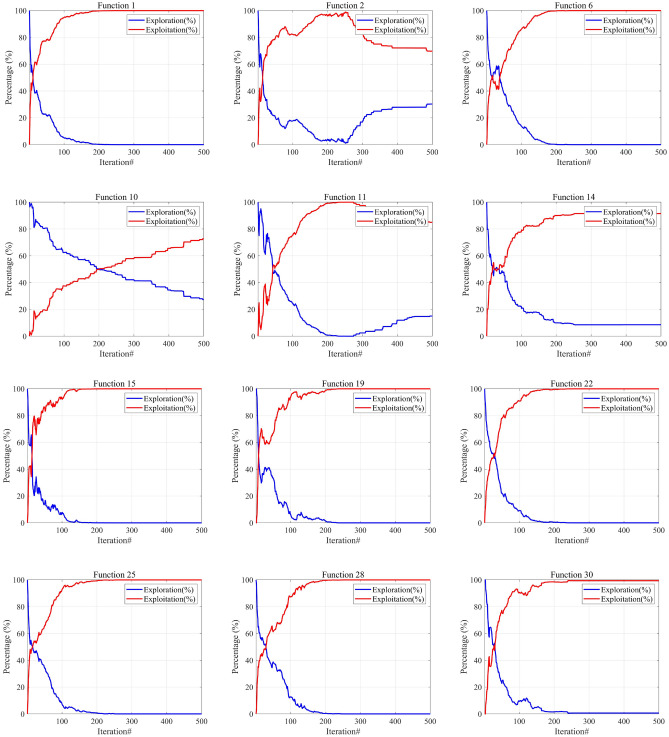
The exploration and exploitation testing results of ISO on the 30-dimensional CEC-2017 benchmark. For all functions, in most cases, the algorithm’s exploration capability is stronger than its exploitation capability during the initial stages of solving. As the number of iterations increases, the exploration and exploitation capabilities of the algorithm decrease and increase, respectively. In the later stages of solving, the exploitation capability of the algorithm becomes stronger than its exploration capability.

Figure 5 intuitively illustrates the balance between exploration and exploitation in ISO using the 30-dimensional CEC-2017 test functions. From the graph, it is evident that the intersection point of the exploration and exploitation ratio in the ISO algorithm.During the initial phase, a comprehensive exploration of the global search space is conducted, gradually transitioning to a local exploitation phase. Notably, in the later iterations of all functions, ISO maintains a relatively high level of exploitation, contributing to an improved convergence rate and search accuracy. Throughout the entire iterative process, ISO preserves a dynamic balance between exploration and exploitation. Consequently, ISO exhibits significant advantages in avoiding local optima and premature convergence.

### Convergence behavior analysis

**Fig. 6:**
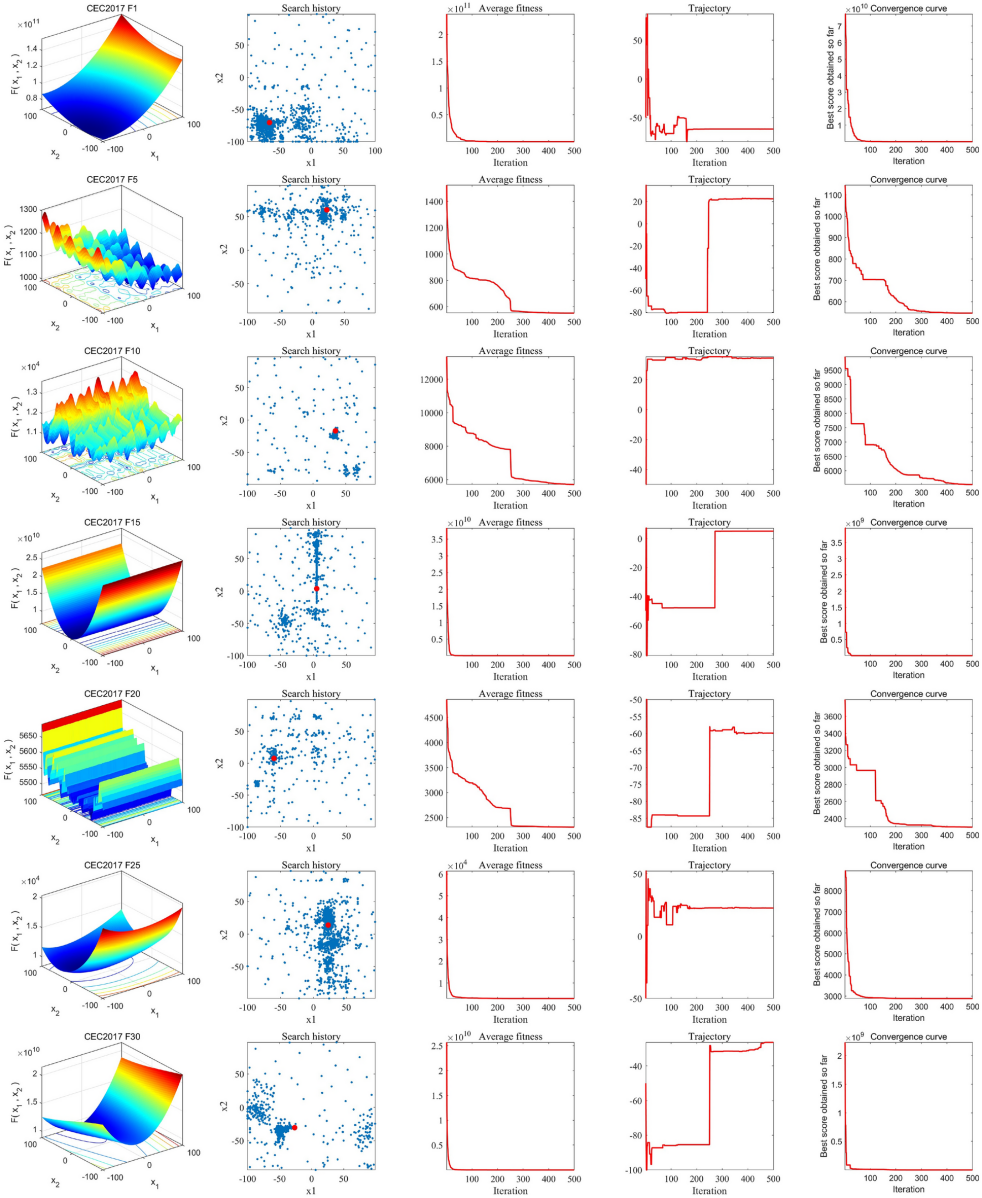
Convergence Behavior of ISO. The first column presents three-dimensional visualizations of the six CEC-2017 benchmark functions. The second column illustrates the search history of the population using the ISO. The third column compares the average fitness values of the SO and ISO algorithms. The fourth column shows the individual search trajectories of both algorithms. Finally, the fifth column displays the convergence curves of both algorithms.

To rigorously validate the convergence properties of ISO, we designed experiments to thoroughly analyze its convergence behavior. As shown in Fig. 6, the first column provides an intuitive visualization of the two-dimensional search space of the test function, vividly illustrating the complexity of the objective function. The second column offers detailed insights into the agents’ search trajectories, where it is evident that most agents tightly converge around the optimal solution while being distributed across the entire search space, demonstrating ISO’s exceptional ability to avoid local optima. The third column depicts the variation in the average fitness value of the search agents, starting with a relatively high value, which reflects the agents’ extensive exploration of the search space, followed by a rapid decline, indicating the agents’ inherent potential to locate the optimal solution. The fourth column showcases the search path of an individual agent, revealing a smooth transition from initial fluctuations to stability, illustrating the balanced shift from global exploration to local exploitation, which aids in achieving the global optimum. The final column presents the convergence curve of ISO, showing a consistent decline for unimodal functions, signifying the algorithm’s gradual approach toward the optimal solution with each iteration, and a stepwise downward trend for multimodal functions, underscoring ISO’s ability to escape local optima and ultimately converge to the global optimum. These experimental findings provide comprehensive insights into ISO’s convergence dynamics and highlight its robustness across different optimization scenarios, forming a strong foundation for further refinement of the algorithm.

## Solving constrained optimization problems using ISO

In this section, we present the mathematical formulation of constrained optimization problems, as well as the approach employed by the ISO algorithm to solve such problems.

### Constrained optimization problems

Constrained optimization problems are widespread in various fields, including engineering, economics, and artificial intelligence, where the objective is to optimize a certain function subject to a set of constraints. These constraints, which can include inequality and equality conditions, must be satisfied by any feasible solution. Mathematically, a constrained optimization problem can be formulated as:

$$\min _{\textbf{x}} f(\textbf{x})$$

where $f(\textbf{x})$ is the objective function and $\textbf{x} \in {\mathbb {R}}^n$ represents the decision variables. The constraints are typically given as:

$$g_i(\textbf{x}) \le 0, \quad i = 1, 2, \dots , m$$

$$h_j(\textbf{x}) = 0, \quad j = 1, 2, \dots , p$$

The primary challenge in solving constrained optimization problems lies in the need to find solutions that not only optimize the objective function but also adhere to the constraint conditions, which are often nonlinear, non-convex, or high-dimensional. This makes such problems computationally challenging, especially in real-world applications where the constraints may change over time or be difficult to express explicitly.

### The ISO algorithm for constrained optimization

The proposed ISO algorithm is a powerful metaheuristic method designed to address complex constraint optimization problems. It incorporates several advanced strategies that allow it to efficiently explore the solution space while ensuring that the solutions remain feasible within the constraints. The key strength of ISO lies in its ability to converge efficiently by balancing exploration (global search) and exploitation (local search) while adaptively handling constraints. At the core of the ISO strategy is the division of the population into two distinct groups-male and female. Each group performs local searches. This cooperative search mechanism ensures that information is exchanged between the two groups, thereby accelerating convergence and reducing the likelihood of premature convergence to local optima. This division promotes both diversity in the search space and specialization in solution improvement. A fundamental feature of ISO is its dynamic penalty mechanism, which assigns penalties to solutions that violate the problem’s constraints. These penalties guide the search process back into the feasible region of the solution space. The penalty factor is adaptive, meaning it changes throughout the optimization process, enabling a smooth transition between exploration (searching broadly for solutions) and exploitation (refining the best solutions). This dynamic adjustment ensures that the search is continuously driven by both the quality and feasibility of the solutions. In addition to the penalty mechanism, ISO employs a repair operation to maintain the feasibility of candidate solutions. Whenever a solution violates a constraint, this repair operation adjusts the solution to bring it back within the feasible region. This ensures that only valid solutions are retained in the population, improving the quality of the search process. The algorithm’s overall design is aimed at achieving a comprehensive and efficient approach to solving various constraint optimization problems. By incorporating these strategies-cooperative search, dynamic penalty adjustment, feasibility repair, and adaptive exploration-exploitation balancing-the ISO algorithm is well-equipped to explore the solution space thoroughly while ensuring the validity and optimality of the solutions. As such, the ISO method presents a robust framework for addressing the challenges of constrained optimization across a wide range of applications.

## Global optimization test

In this section, we compare ISO with six high-performance competing algorithms and evaluate its performance using 23 classic benchmark functions to analyze its capability in solving complex global optimization problems.The hardware configuration used in this experiment includes an Intel i9-13900K CPU, 32GB of memory, and a 4070 GPU. The software configuration consists of the Windows 10 operating system and MATLAB 2023a. The performance of the tested algorithms demonstrates significant strength, as each algorithm shows strong capabilities in solving various optimization problems across the 23 test functions. These algorithms are known for their reliable convergence, efficiency, and adaptability when dealing with complex objective functions. Their robust performance in minimizing errors and achieving optimal values further underscores their applicability in a wide range of optimization scenarios.

To rigorously assess the performance of the ISO algorithm, it was compared against several highly-cited and state-of-the-art algorithms. Specifically, we selected SO, RIME, SCA, WOA, GWO, and COA^55^ as competing algorithms. Among them, SO (proposed in 2020), RIME (poposed in 2023), and COA (poposed in 2024) represent some of the latest advancements in optimization methods. Additionally, SCA^56^, WOA^57^, and GWO^58,59^ are well-established algorithms with a significant academic impact, having been cited 4,524 times, 11,027 times, and 15,545 times, respectively. These algorithms, widely regarded for their performance and effectiveness in various optimization tasks, serve as benchmarks in validating the robustness of the ISO algorithm. Table 2 shows the parameter settings of ISO and six competing algorithms in detail, which explains the algorithms used for the experiment in more detail and rigor. By positioning ISO against such well-cited and contemporary methods, its performance can be evaluated in a broader context, further emphasizing its strengths and areas for future improvement.

**Table 2:** Parameter settings of ISO and six competing algorithms.

Algorithm	Parameter settings
SCA	$a=2, r 2=2 \times \pi \times r a n d, r 3=2 \times r a n d, r 4=r a n d$.
GWO	*a* : Linearly decreased from 2 to 0 .
WOA	*a* : decreases linearly from 2 to $0, \textrm{a}_2$ : linearly dicreases from -1 to $-2, b=1$.
RIME	$W=5, r 1=$ rand, $r 2=$ rand .
COA	Parameter-free
SO	Threshold $=0.25$, Thresold $2=0.6, C 1=0.5, C 2=0.05, C 3=2$.
ISO	Threshold $=0.15$, Thresold $2=0.6, C 1=0.5, C 2=0.05, C 3=2, W=5$

**Fig. 7:**
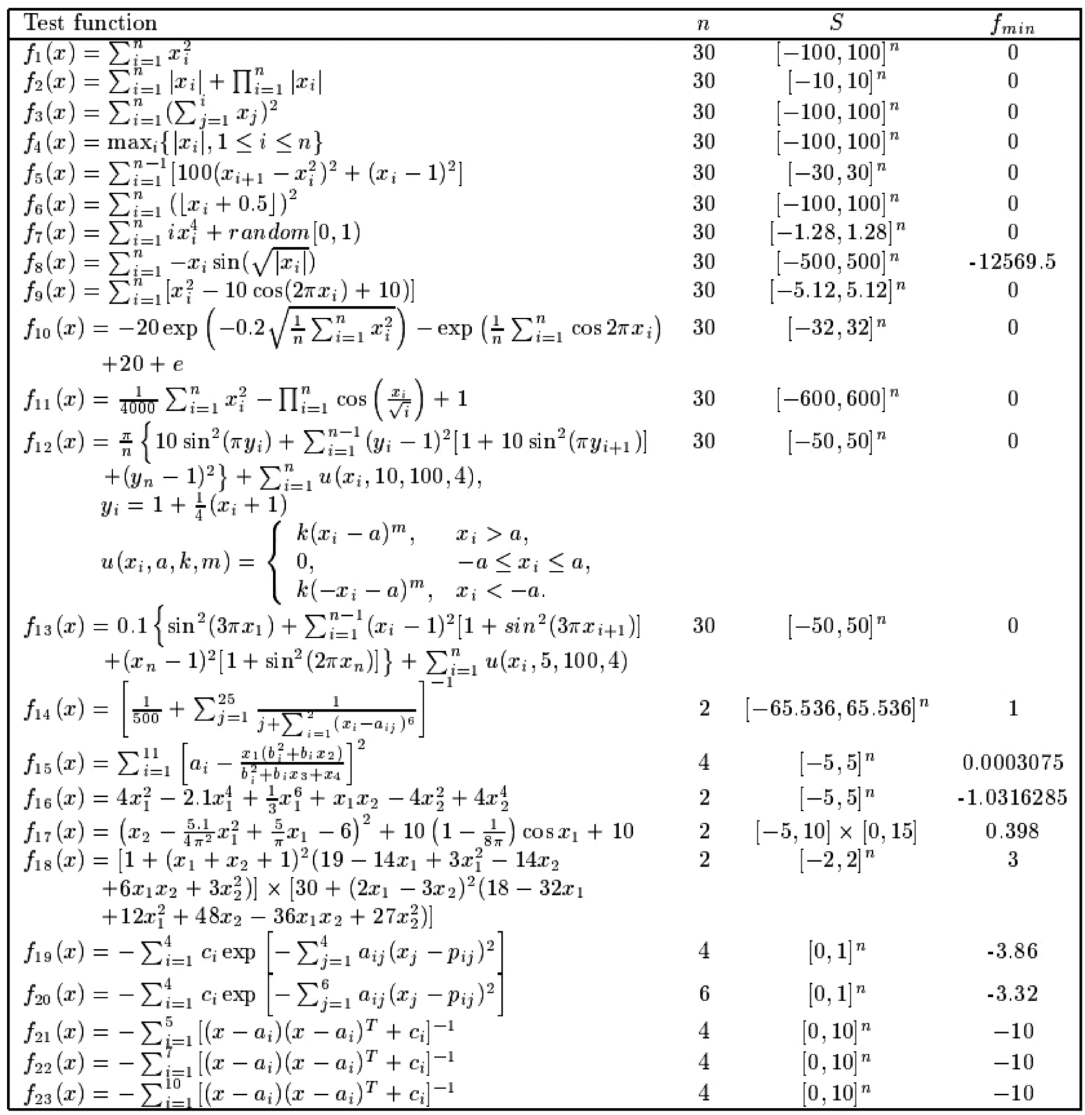
The 23 classic benchmark functions (also referred to as CEC-2005). The first column presents the mathematical expressions of the 23 functions, the second column lists the dimensions of the functions, the third column specifies the value ranges of the functions, and the fourth column provides the minimum values of the functions.

We selected 23 standard test functions. They are specifically designed to address significant challenges in optimization, becoming the gold standard for performance comparison in the field of metaheuristic algorithms, as shown in Fig. 7^59^. Functions Fl to F7 are uni-modal functions with a single global optimum, utilized to evaluate the algorithm’s developmental capabilities. Functions F8 to F13 are multimodal functions with one global optimum and several local optima, enabling the testing of the algorithm’s global search capabilities. Functions F14 to F23 are multimodal functions with fixed dimensions, suitable for simultaneously testing the algorithm’s developmental and search capabilities.

### Classic benchmark function test

**Fig. 8:**
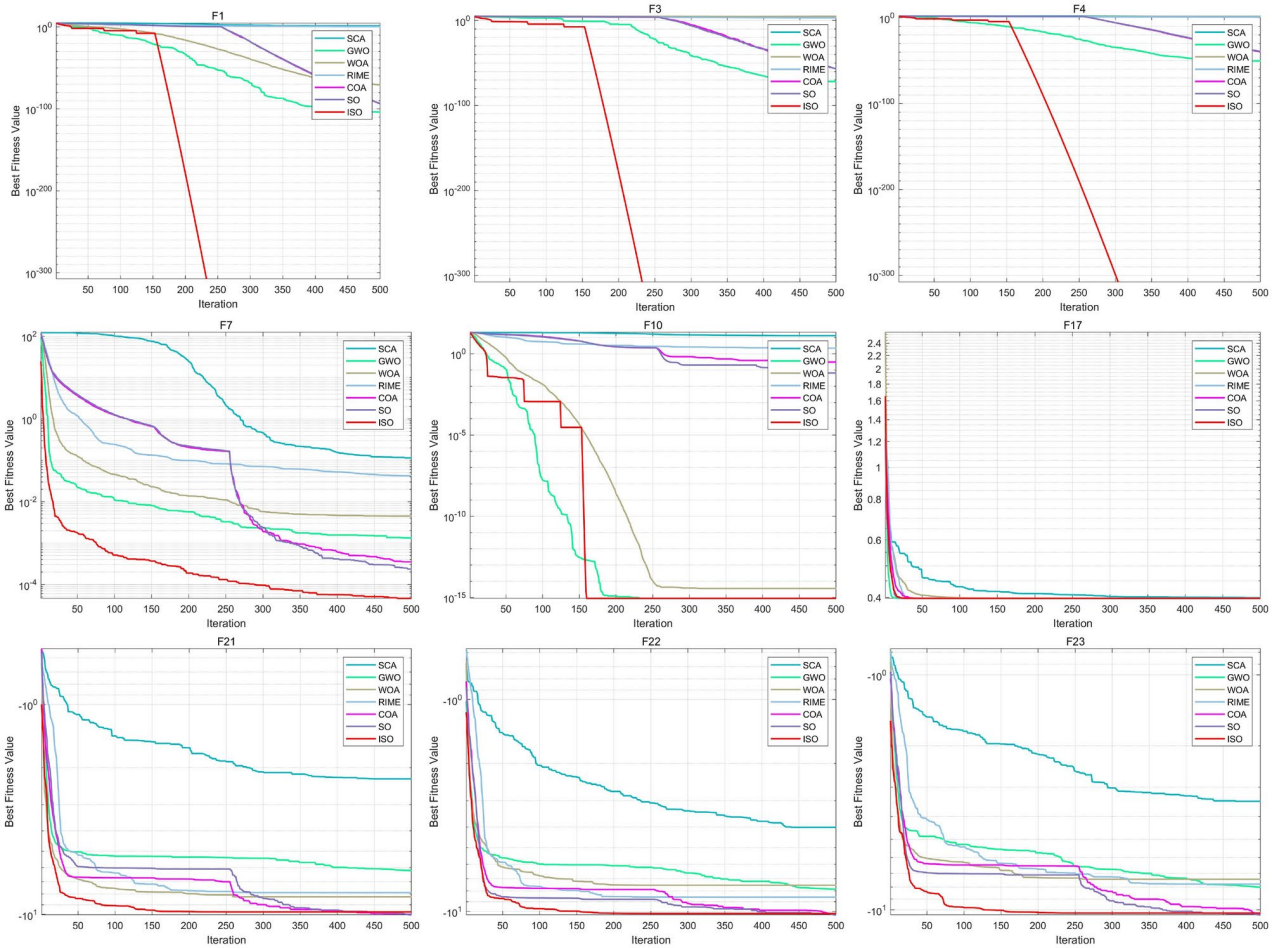
The experimental results of 23 classic benchmark function. In most cases, ISO exhibits faster convergence and superior capability in finding the optimal values compared to the six competitive algorithms across the 23 benchmark functions.

**Fig. 9:**
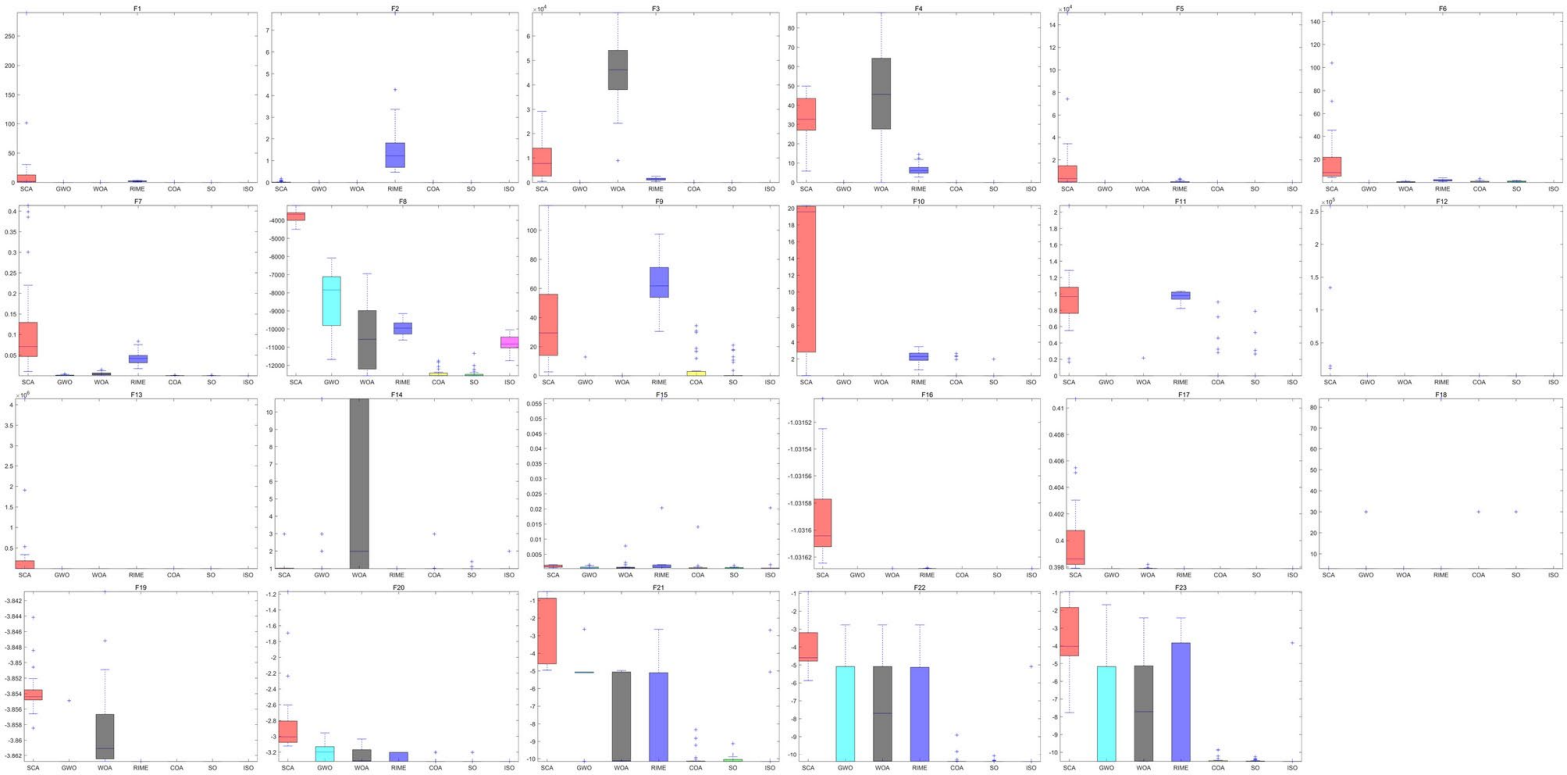
The box plots for the performance of seven different algorithms on the 23 benchmark functions clearly demonstrate that ISO exhibits the best performance in almost all cases.

The details of the classical benchmark functions are presented in Fig. 7, with Fig. 8 highlighting that the ISO algorithm performs better than other algorithms on test functions F1, F3, F4, F7, F10, F17, F21, F22, and F23. In these cases, ISO achieves results with fewer iterations. Figure 9 presents box plots based on the performance results of different algorithms across the 23 benchmark functions. The detailed performance results of seven different algorithms on the 23 benchmark functions are provided in Table 3, which includes key metrics such as Maximum Value, Minimum Value, Mean Value, and Standard Deviation. For the Wilcoxon rank-sum tests, a significance threshold of 5% was applied: a p-value below 5% indicates a statistically significant difference between the two algorithms, while a p-value above 5% suggests no significant distinction. Instances where the result is denoted as NaN indicate that the performances of the two algorithms are comparable and cannot be directly compared. The results of these statistical tests are summarized in Table 3.

**Table 3:** The experimental results of 23 classic benchmark functions.

		SCA	GWO	WOA	RIME	COA	SO	ISO
F1	Maximum Value	289.676	3.89E-103	4.35E-70	3.544242	9.89E-94	1.96E-93	0
	Minimum Value	0.000197	3.75E-154	1.33E-83	0.901612	1.18E-99	3.42E-99	0
	Mean Value	19.22186	1.47E-104	1.92E-71	2.17591	1.32E-94	2.06E-94	0
	Standard Deviation	54.65929	7.13E-104	8.22E-71	0.686074	2.76E-94	4.28E-94	0
F2	Maximum Value	0.177034	8.76E-56	2.66E-50	7.799352	5.72E-42	6.95E-42	0
	Minimum Value	0.00011	2.14E-90	2.28E-57	0.468725	2.40E-45	1.41E-45	0
	Mean Value	0.023353	2.92E-57	1.31E-51	1.541588	5.47E-43	5.89E-43	0
	Standard Deviation	0.041002	1.60E-56	4.90E-51	1.442036	1.22E-42	1.30E-42	0
F3	Maximum Value	29216.54	5.45E-71	69518.61	2579.32	6.53E-56	3.95E-56	0
	Minimum Value	460.86	7.69E-150	8903.104	473.0651	1.35E-64	1.82E-66	0
	Mean Value	8707.964	1.82E-72	46136.2	1336.204	3.09E-57	1.70E-57	0
	Standard Deviation	6946.543	9.95E-72	14170.73	449.3172	1.26E-56	7.33E-57	0
F4	Maximum Value	49.9207	1.02E-49	87.85811	14.50151	3.82E-39	1.01E-38	0
	Minimum Value	5.845735	5.24E-87	0.00112	2.820589	2.09E-43	3.71E-43	0
	Mean Value	33.5084	4.41E-51	42.96508	6.697529	2.28E-40	7.35E-40	0
	Standard Deviation	11.33993	1.93E-50	24.42778	2.795459	6.95E-40	2.00E-39	0
F5	Maximum Value	150709.3	26.78094	28.76342	3209.399	28.95667	28.97395	28.49889
	Minimum Value	70.78773	25.19421	27.00141	45.82914	1.144671	0.173366	24.22749
	Mean Value	14630.84	25.82226	27.85565	612.2056	24.67858	20.01334	25.67873
	Standard Deviation	30251.64	0.283913	0.507351	886.8687	8.991593	12.15285	0.889221
F6	Maximum Value	147.5737	0.014402	1.307712	4.004147	3.323682	1.62982	2.70E-07
	Minimum Value	4.26472	9.48E-06	0.08342	0.785151	0.012831	0.000393	5.59E-08
	Mean Value	22.42322	0.000924	0.437586	1.992807	0.828968	0.826771	1.47E-07
	Standard Deviation	32.53068	0.002626	0.301863	0.791337	0.685515	0.484683	5.16E-08
F7	Maximum Value	0.413655	0.004126	0.014671	0.083596	0.001666	0.001529	0.000254
	Minimum Value	0.010242	4.69E-05	4.37E-05	0.01735	1.09E-05	2.32E-05	3.90E-06
	Mean Value	0.114315	0.001335	0.004478	0.042367	0.000351	0.000236	4.61E-05
	Standard Deviation	0.114316	0.000992	0.003764	0.015144	0.00035	0.000281	5.30E-05
F8	Maximum Value	-3186.29	-6074.32	-6948.91	-9144.51	-11759.2	-11344.1	-10042.7
	Minimum Value	-4497.93	-11672.6	-12569.4	-10605	-12569.4	-12569.3	-11740.4
	Mean Value	-3781.86	-8520.82	-10507.5	-9955.22	-12448.9	-12454	-10765.2
	Standard Deviation	335.6797	1720.527	1715.336	393.3259	212.7875	247.2472	404.1246
F9	Maximum Value	117.0219	12.88607	5.68E-14	97.33039	34.38944	20.99379	0
	Minimum Value	2.599611	0	0	30.40351	0	0	0
	Mean Value	37.36069	0.429536	3.79E-15	64.00338	5.215658	3.105711	0
	Standard Deviation	30.01211	2.352664	1.44E-14	16.9998	10.18298	6.325755	0
F10	Maximum Value	20.32319	8.88E-16	7.99E-15	3.466336	2.643174	1.972303	8.88E-16
	Minimum Value	0.021025	8.88E-16	8.88E-16	0.701275	4.44E-15	4.44E-15	8.88E-16
	Mean Value	12.92944	8.88E-16	3.61E-15	2.216525	0.307756	0.065743	8.88E-16
	Standard Deviation	8.910452	0	2.22E-15	0.662619	0.803628	0.360092	0
F11	Maximum Value	2.080957	0	0.216218	1.033782	0.901081	0.788433	0
	Minimum Value	0.166406	0	0	0.822567	0	0	0
	Mean Value	0.936261	0	0.007207	0.96991	0.089579	0.063019	0
	Standard Deviation	0.340515	0	0.039476	0.055943	0.226275	0.180645	0
F12	Maximum Value	258908.4	0.006622	0.050303	6.111584	0.767537	2.984032	0.00666
	Minimum Value	0.713079	5.31E-08	0.006866	0.611823	0.000823	0.000226	4.81E-09
	Mean Value	13970.71	0.000279	0.021954	3.326231	0.078896	0.231012	0.000862
	Standard Deviation	52336.42	0.001227	0.011474	1.313808	0.158201	0.595674	0.002236
F13	Maximum Value	4144190	1.754106	1.074529	0.622379	2.997035	2.991735	2.17262
	Minimum Value	4.058753	0.011459	0.148864	0.09011	0.000511	0.005059	8.59E-08
	Mean Value	276998.7	0.50451	0.471298	0.271559	0.593331	0.505044	0.718383
	Standard Deviation	813864.7	0.38309	0.24732	0.125269	1.042795	0.850379	0.874078
F14	Maximum Value	2.982105	10.76318	10.76318	0.998004	2.982105	1.395186	1.992031
	Minimum Value	0.998004	0.998004	0.998004	0.998004	0.998004	0.998004	0.998004
	Mean Value	1.39855	2.139929	4.229676	0.998004	1.064914	1.015193	1.064272
	Standard Deviation	0.805347	2.970067	4.14191	3.07E-12	0.362124	0.074802	0.252193
F15	Maximum Value	0.00157	0.001489	0.007763	0.056545	0.014078	0.001327	0.020363
	Minimum Value	0.000578	0.000307	0.000315	0.000312	0.000308	0.000308	0.000307
	Mean Value	0.001031	0.000737	0.000877	0.006495	0.000973	0.000522	0.004371
	Standard Deviation	0.000348	0.00035	0.001359	0.014848	0.002486	0.00023	0.008136
F16	Maximum Value	-1.0315	-1.03163	-1.03163	-1.03163	-1.03163	-1.03163	-1.03163
	Minimum Value	-1.03162	-1.03163	-1.03163	-1.03163	-1.03163	-1.03163	-1.03163
	Mean Value	-1.03159	-1.03163	-1.03163	-1.03163	-1.03163	-1.03163	-1.03163
	Standard Deviation	3.20E-05	5.98E-16	1.91E-09	1.07E-07	5.53E-16	5.30E-16	9.84E-16
F17	Maximum Value	0.410699	0.397887	0.398196	0.39789	0.397887	0.397887	0.397887
	Minimum Value	0.397915	0.397887	0.397887	0.397887	0.397887	0.397887	0.397887
	Mean Value	0.399935	0.397887	0.397907	0.397888	0.397887	0.397887	0.397887
	Standard Deviation	0.002885	0	5.70E-05	6.53E-07	0	0	4.52E-13
F18	Maximum Value	3.00114	30	3.001592	84.00014	30	30	3
	Minimum Value	3	3	3	3	3	3	3
	Mean Value	3.000108	3.9	3.000098	5.700005	6.6	6.6	3
	Standard Deviation	0.000213	4.929503	0.000305	14.78853	9.335139	9.335139	1.89E-15
F19	Maximum Value	-3.84417	-3.8549	-3.84085	-3.86278	-3.86278	-3.86278	-3.86278
	Minimum Value	-3.85844	-3.86278	-3.86278	-3.86278	-3.86278	-3.86278	-3.86278
	Mean Value	-3.85377	-3.86199	-3.85873	-3.86278	-3.86278	-3.86278	-3.86278
	Standard Deviation	0.002506	0.002405	0.005189	2.40E-07	2.51E-15	2.45E-15	2.57E-15
F20	Maximum Value	-1.16826	-2.95642	-3.03314	-3.20303	-3.2031	-3.2031	-3.322
	Minimum Value	-3.12307	-3.322	-3.32178	-3.32199	-3.322	-3.322	-3.322
	Mean Value	-2.85032	-3.19212	-3.25116	-3.26649	-3.31803	-3.31407	-3.322
	Standard Deviation	0.440651	0.096175	0.092789	0.060322	0.021707	0.030164	1.55E-12
F21	Maximum Value	-0.49642	-2.63047	-4.96967	-2.63039	-8.32736	-9.12275	-2.68286
	Minimum Value	-4.95624	-10.1532	-10.1529	-10.1532	-10.1532	-10.1532	-10.1532
	Mean Value	-2.26096	-6.17625	-8.24956	-7.87782	-10.0023	-10.0652	-9.73425
	Standard Deviation	1.822012	2.273458	2.480985	2.681787	0.42948	0.200927	1.624491
F22	Maximum Value	-0.90756	-2.7659	-2.76326	-2.76579	-8.91203	-10.085	-5.08767
	Minimum Value	-5.87089	-10.4029	-10.4018	-10.4029	-10.4029	-10.4029	-10.4029
	Mean Value	-4.01077	-7.85094	-7.51982	-8.56719	-10.3255	-10.387	-10.2258
	Standard Deviation	1.24349	2.804623	2.958981	2.902378	0.287143	0.059191	0.970431
F23	Maximum Value	-0.94187	-1.67655	-2.41946	-2.42171	-9.86149	-10.2798	-3.83543
	Minimum Value	-7.76403	-10.5364	-10.5357	-10.5363	-10.5364	-10.5364	-10.5364
	Mean Value	-3.44927	-7.99568	-7.40227	-7.76389	-10.4629	-10.5072	-10.313
	Standard Deviation	1.767949	3.287719	3.263484	3.537858	0.173013	0.060396	1.223427

In most cases, ISO exhibits faster convergence and superior capability in finding the optimal values compared to the six competitive algorithms across the 23 classic benchmark functions.

**Table 4:** Wilcoxon rank-sum test results for the performance of seven different algorithms on 23 benchmark functions.

SCA	GWO	WOA	RIME	COA	SO	ISO
1.21E-12	1.21E-12	1.21E-12	1.21E-12	1.21E-12	1.21E-12	NaN
1.21E-12	1.21E-12	1.21E-12	1.21E-12	1.21E-12	1.21E-12	NaN
1.21E-12	1.21E-12	1.21E-12	1.21E-12	1.21E-12	1.21E-12	NaN
1.21E-12	1.21E-12	1.21E-12	1.21E-12	1.21E-12	1.21E-12	NaN
3.02E-11	0.245813802	1.41E-09	3.02E-11	6.77E-05	0.090490361	1
3.02E-11	3.02E-11	3.02E-11	3.02E-11	3.02E-11	3.02E-11	1
3.02E-11	9.92E-11	8.99E-11	3.02E-11	1.16E-07	1.25E-07	1
3.02E-11	4.12E-06	0.911708975	1.31E-08	3.02E-11	3.34E-11	1
1.21E-12	0.333710696	0.160741998	1.21E-12	1.93E-10	1.31E-07	NaN
1.21E-12	NaN	8.07E-08	1.21E-12	1.20E-13	6.14E-14	NaN
1.21E-12	NaN	0.333710696	1.21E-12	0.011035086	0.021577192	NaN
3.02E-11	8.84E-07	3.02E-11	3.02E-11	4.62E-10	6.12E-10	1
3.02E-11	0.332854692	0.118817344	0.270705338	0.93519197	0.773119942	1
1.29E-09	0.019733828	1.26E-10	3.91E-09	0.001586287	0.000186753	1
0.000397325	0.001947012	0.001170045	5.94E-05	0.00349206	0.002617066	1
1.48E-11	0.105849154	1.48E-11	1.48E-11	0.883674537	0.548903508	1
1.72E-12	0.333710696	1.72E-12	1.72E-12	0.333710696	0.333710696	1
2.77E-11	0.138998914	2.77E-11	2.77E-11	0.001199277	0.004184834	1
1.04E-11	0.853880751	1.04E-11	1.04E-11	0.23837805	0.032437277	1
1.72E-11	9.40E-06	1.72E-11	1.72E-11	8.66E-05	2.15E-05	1
4.96E-11	4.93E-06	1.94E-09	3.69E-09	0.001587743	0.005867001	1
2.71E-11	0.001214672	9.93E-11	2.63E-10	0.050124243	0.317261968	1
1.14E-10	0.001353814	2.73E-10	2.04E-10	0.003450636	0.000378365	1

The results show that, in most cases, ISO exhibits a statistically significant difference compared to the six competing algorithms.

In conclusion, the experimental results show that the ISO algorithm consistently outperforms other algorithms across several benchmark test functions, including F1, F3, F4, F7, F10, F17, F21, F22, and F23. In these instances, ISO not only achieves superior results but does so with fewer iterations, which emphasizes its efficiency in both convergence speed and solution quality. This indicates that the ISO algorithm is particularly effective in solving these test functions, requiring fewer computational resources to reach optimal or near-optimal solutions. The box plot results, presented in Fig. 9, offer a visual and intuitive illustration of ISO’s robust performance compared to the other algorithms across the 23 benchmark functions, further reinforcing its strong solving capability.^60^

The detailed performance results for seven different algorithms on the 23 benchmark functions, including key metrics such as Maximum Value, Minimum Value, Mean Value, and Standard Deviation, are comprehensively provided in Table 3. This table underscores the exceptional performance of ISO in comparison to the other algorithms. Additionally, the results of the Wilcoxon rank-sum tests, as summarized in Table 4, reveal statistically significant differences between ISO and the competing algorithms. Most importantly, the p-values for these tests are below the 5% significance threshold, which indicates that ISO exhibits a statistically significant performance advantage over the other algorithms in solving the majority of the test functions^61^.

### CEC-2011 benchmark function test

**Fig. 10:**
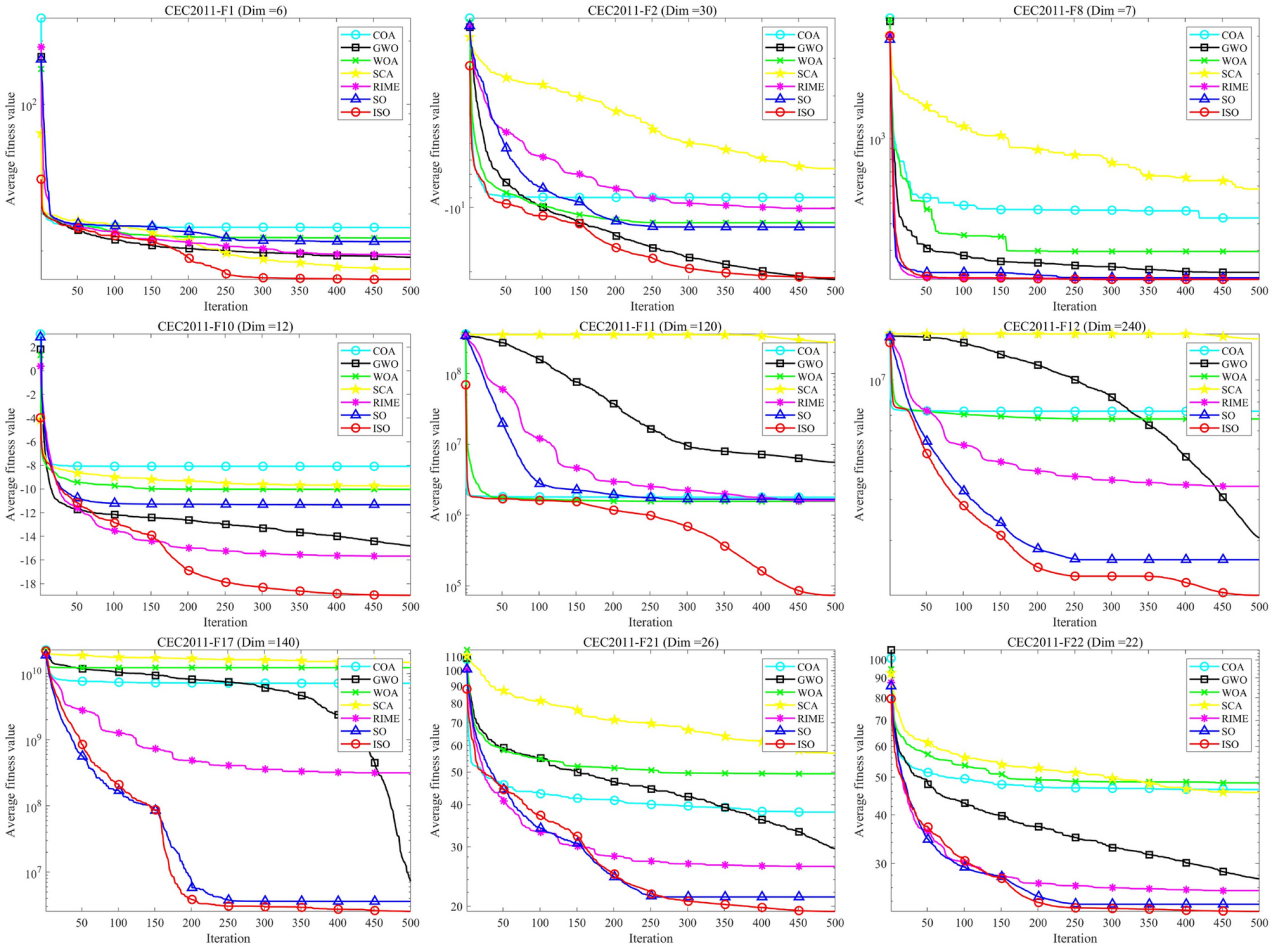
The experimental results of CEC-2011 benchmark functions. In most cases, ISO exhibits faster convergence and superior capability in finding the optimal values compared to the six competitive algorithms across the CEC-2011 benchmark functions.

The details of the CEC-2011 benchmark functions are presented in Table 5. Figure 10 presents the convergence curves based on the performance results of different algorithms on test functions F1, F2, F8, F10, F11, F12, F17, F21, and F22. The detailed performance results of various algorithms on the CEC-2011 benchmark functions are provided in Table 6, which includes the average values and standard deviations. The results of the statistical tests are summarized in Table 7.

**Table 5:** The CEC-2011 real-world problem benchmark functions.

Problem No. (as they should appear in the participant papers)	No. of Dimensions	Constraints	Bounds	Matlab Folder: Prob_10_Cire _Ante nna			
1. Parameter Estimation for Frequency Modulated (FM) Sound Waves Matlab Folder: Probs_1_to_8	6	Bound constrained	All dimensions bound between [-6.4, 6.35]	11. The ELD Problems Matlab Folder: Probs_11_ELD _Pac kage 11.1 DED instance 1 11.2 DED instance 2 11.3 ELD instance 1	120 216 (The objective function takes 240 parameters, but 24 of them are fixed at 55 and need not be optimized) 6	Inequality constraints Inequality constraints Inequality constraints	$P_{min}$=[10, 20, 30, 40, 50;] $P_{max}$=[75, 125, 175, 250, 300;] Lower_Limit=repmat(Pmin,1.24); Upper_Limik = repmat(Pmax, 1.24), (in MATLAB) $P_{min}$=[150, 135, 73, 60, 73, 57, 20, 47, 20]; $P_{max}$=[470, 460, 340, 300, 243, 160, 130, 120, 80]; Lower_Limit=repmat(Pmin, 1.24); Upper_Limit = repmat(Pmax, 1.24) (in MATLAB) 6 unit limits (in format [LB1, UB1; LB2, UB2;...]); [100, 500 ; 50, 200; 50, 300; 50, 150; 50, 200; 50, 120;]
2. Lennard-Jones Potential Problem Matlab Folder: Probs_1_to_8	$3 \times 10=30$ (10 atcen problem)	Bound constrained	Let $\vec {x}$ be the variable of the problem, which has three components for tree atoms, six components for 4 atoms and so on. The first variable due to the second atom i.e. $x_1 \in [0,4]$, then the second and third variables are such that $x_2 \in [0,4]$ and $x_3 \in [0, \pi ]$. The coordinates $x_i$ for any other atom is taken to be bound in the range: $\left[ -4-\frac{1}{4}\left[ \frac{i-4}{3}\right] , 4+\frac{1}{4}\left[ \frac{i-4}{3}\right] \right]$, where $\lfloor r\rfloor$ is the nearest least integer w.r.t. $r \in \square$.				
3. The Bifunctional Catalyst Blend Optimal Control Problem Matlab Folder: Probs_1_to_8	1	Bound constrained	[0.6, 0.9]				
4. Optimal Control of a Non-Linear Stirred Tank Reactor Matlab Folder: Probs_1_to_8	1	Unconstrained	No bound, initialization in the range [0, 5]				
11.4 ELD Instance 2 11.5 ELD Instance 3 11.6 ELD Instance 4 11.7 ELD Instance 5	13 15 40 140	Inequality constraints Inequality constraints Inequality constraints Inequality constraints	13 unit limits (in format [LB1, UB1; LB2, UB2; ...]); [0, 680, 0, 360, 0, 360, 60, 180, 60, 180, 60, 180, 60, 180, 60, 180, 60, 160, 40, 120; 40, 120, 55, 120, 55, 120]; 15 unit limits (in format [LB1, UB1; LB2, UB2; ....]) [130, 455; 150, 455; 20, 130; 20, 130; 150, 470; 135, 460; 135, 465; 60, 300; 25, 162; 25, 160; 20, 80; 20, 80; 25, 85; 15, 55; 15, 55;] 40 unit limits (in format [LB1, UB1; LB2, UB2; ....]): [36, 114; 36, 114; 60, 120; 80, 190; 47, 97; 68, 140; 110, 300; 135,300; 135, 300; 130, 300; 94, 375; 94, 375; 125, 500; 125, 500; 125, 500; 125, 500; 220,500; 220, 500; 242,550; 242, 550; 254, 550; 254, 550; 254, 550; 254, 550; 254, 550; 254, 550; 10, 150; 10, 150; 10, 150; 47, 97; 60, 190; 60, 190; 60, 190; 90, 200; 90, 200; 90, 200; 25, 110; 25, 110; 25, 110; 242, 550;]; 140 unit limits (in format [LB1, UB1; LB2, UB2; ...]): [71, 119; 120, 189; 125, 190; 125, 190; 90, 190; 90, 190; 260, 400; 280, 490; 260, 496; 260, 496; 260, 496; 260, 496; 260, 506; 260, 509; 260, 506; 260, 505; 260, 506; 260, 506; 260, 505; 260, 505; 260, 505; 60, 505; 260, 505; 260, 505; 280, 537; 280, 537; 280, 549; 280, 549; 260, 501; 260, 501; 260, 506; 260, 506; 260, 506; 260, 506; 260, 500; 260, 500; 120, 241; 120, 241; 423, 774; 423, 769; 319, 328; 160, 250; 160, 250; 160, 250; 160, 250; 160, 250; 160, 250; 160, 250; 160, 250; 165, 504; 165, 504; 165, 304; 165, 504; 180, 471; 180, 561; 103, 341; 198, 617; 100, 312; 153, 471; 163, 500; 95, 302; 160, 511; 160, 511; 196, 490; 196, 490; 196, 490; 196, 490; 130, 432; 130, 432; 137, 455; 137, 455; 195, 541; 175, 536; 175, 540; 175, 538;]	11.8 Hydrothermal Scheduling Instance 1 11.9 Hydrothermal Scheduling Instance 2 11.10 Hydrothermal Scheduling Instance 3	96 96 96	Inequality constraints Inequality constraints Inequality constraints	[175, 540; 330, 574; 160, 531; 160, 531; 200, 342; 56, 132; 115, 245; 115, 245; 115, 245; 207, 307; 207, 307; 175, 345; 175, 345; 175, 345; 175, 345; 360, 580; 415, 645; 795, 984; 795, 978; 578, 682; 615, 720; 612, 718; 612, 720; 758, 964; 755, 958; 750, 1007; 750, 1005; 713, 1013; 718, 1020; 791, 954; 786, 952; 795, 1006; 795, 1013; 795, 1021; 795, 1015, 94, 203; 94, 203; 94, 203; 244, 379; 244, 379; 244, 379; 95, 190; 95, 189; 116, 194; 175, 321; 2, 19; 4, 59; 15, 83; 9, 53; 12, 37; 10, 34; 112, 373; 4, 20; 5, 38; 5, 19; 50, 95; 5, 10; 42, 74; 42, 74; 41, 105; 17, 51; 7, 19; 7, 19; 26, 40;]; $$Q_{{min}} = \left[ {5\,\,6\,\,10\,\,13} \right];Q_{{max}} = \left[ {15\,\,15\,\,30\,\,25} \right];$$ Lower_Limit = repmat($$Q_{min} 1, 24);$$ Upper_Limit = repmat($$Q_{max} 1, 24);$$ Same as 11.8 Same as 11.8
				12. Messenger: Spacecraft Trajectory Optimization Problem Matlab Folder: Probs_12_to_13 _Pac kage	26	Bound constraints	
				13. Cassini 2: Spacecraft Trajectory Optimization Problem	22	Bound constraints	

The table provides a detailed description of each problem’s name, dimensions, constraints, and bounds.

**Table 6:** Experimental results of seven different algorithms on the CEC-2011 benchmark functions, presenting the mean and standard deviation for each function run by the seven algorithms.

Metric	COA	GWO	WOA	SCA	RIME	SO	ISO
Ave	25.85904526	18.68346041	23.06790421	16.40490796	19.25866088	22.17915786	14.64978448
Std	2.451358892	5.901118389	4.737964007	4.638824406	6.852844371	5.75093018	5.124227465
Ave	-8.975838962	-21.73756999	-11.79459701	-6.564341294	-10.10414016	-12.35418887	-21.34707753
Std	1.770917542	2.79605862	3.189728818	1.731308511	2.319149392	2.929043942	2.889722873
Ave	14.19191198	14.32479269	14.25844231	14.75454365	14.53423838	14.37558448	14.44141134
Std	0.224850038	0.111747104	0.183560922	0.398858708	0.902966565	0.434079684	1.250227885
Ave	-15.14529992	-31.34451913	-23.73377124	-17.20000891	-30.87433359	-31.07021545	-29.82779134
Std	2.09840959	2.525332014	3.702232351	1.129865485	2.591649756	2.904843952	3.139172229
Ave	-0.743817457	-21.26225688	-15.26538641	-9.075469704	-22.20400065	-21.6639018	-21.91846632
Std	10.39981455	2.746213698	2.846889911	2.195995394	2.266204006	2.190157776	2.800570147
Ave	2.171183206	1.156547564	2.146709443	2.314685851	1.534960712	1.722572659	1.254967431
Std	0.232110941	0.339540313	0.26723447	0.195337504	0.174883691	0.321895621	0.141093486
Ave	426.053079	237.4	297.3333333	581.1455953	222.7333333	224.0666667	220
Std	320.4667052	25.55939668	28.16984611	336.4373808	14.97108324	7.565499429	0
Ave	1093929.635	48637.14193	498225.747	511326.472	107295.2455	115457.6703	23804.35792
Std	146397.0424	25302.56378	111300.7204	102134.0244	31000.37334	49556.45736	7645.42468
Ave	-8.082753043	-14.80944206	-10.03005803	-9.742026027	-15.67383872	-11.3225446	-18.98510921
Std	0.695115664	3.63346697	0.852888981	0.779319081	3.44916253	0.722108083	1.598177677
Ave	1794235.331	5592711.049	1566337.939	277554558	1596593.205	1678774.108	73577.80711
Std	73708.80388	433815.3293	101248.5757	29835089.23	344043.1407	256859.5698	25930.13646
Ave	7292718.796	2052202.889	6749859.398	15076976.15	3438024.441	1647171.386	1153031.545
Std	153219.9392	292439.3041	358147.8561	604204.4809	292981.6909	174558.0009	65146.56768
Ave	15456.11019	15522.7668	15569.19637	15984.96057	15538.68923	15480.44691	15461.25747
Std	7.396801586	33.94865265	186.5010339	344.364093	61.50543327	21.69548403	15.90173347
Ave	19196.93235	19544.18613	19445.59974	22618.62156	20321.00721	20314.72072	19305.96828
Std	209.6152613	257.544863	136.4733018	4333.685396	1161.708584	1448.007386	145.7470837
Ave	2474476.986	33103.46009	211826.4662	181124.9772	33143.19814	33135.08191	33107.3383
Std	2832903.639	113.2941208	189366.9424	72464.90023	129.4551529	73.89360882	51.47018902
Ave	143356.8036	143042.1541	147700.0688	1490821.599	150441.3981	147224.1036	144246.7364
Std	5027.092647	5859.412949	5669.71094	1368738.046	7150.053621	5377.248629	4276.791157
Ave	7096779804	7256381.432	12218875061	14712813130	316446350.2	3595861.643	2551542.446
Std	1066257645	20075250.28	2651723346	1758144679	358601013.7	1011289.242	592890.6074
Ave	24181095.89	1116232.648	11656792.62	47163662.3	1441958.659	1188510.933	1124476.672
Std	6639891.603	104689.2648	7480042.997	9000825.475	573444.0854	299443.7684	227206.5349
Ave	23347013.24	1643006.99	16240666.8	45369706.98	1869743.428	1772772.243	1964258.568
Std	5830586.189	206973.1755	10809347.27	8415244.833	354283.1554	298500.2087	318191.0208
Ave	22985892.63	1152502.667	15482055.93	41436886.93	1317736.655	1109938.505	1112280.284
Std	6404711.364	207966.6189	13610674.16	10049232.06	485500.8722	207143.0234	173183.4985
Ave	38.04788875	29.63342455	49.45383259	56.92694839	26.26062405	21.3059085	19.31199209
Std	3.509516931	6.67613462	8.53326163	7.310651786	5.779908641	3.212254709	3.275337823
Ave	46.47668187	27.3986487	48.34152455	45.64710736	25.55480009	23.57913516	22.60730634
Std	5.032627751	3.411642779	8.680274327	5.06171515	3.738524992	2.674471043	2.910832586

**Table 7:** Wilcoxon rank-sum test results for the performance of seven different algorithms on CEC-2011 benchmark functions.

COA	GWO	WOA	SCA	RIME	SO	ISO
9.7555E-10	0.00037704	7.08811E-08	0.001003532	0.01383162	2.49131E-06	1
3.01986E-11	0.473346557	9.91863E-11	3.01986E-11	3.01986E-11	2.87158E-10	1
0.002013771	2.32804E-08	1.76958E-06	3.03616E-09	5.26911E-06	0.06200766	1
3.01986E-11	0.063532651	2.57212E-07	3.01986E-11	0.180899533	0.180899533	1
3.01986E-11	0.363222313	1.17374E-09	3.01986E-11	0.795845542	0.773119942	1
3.01986E-11	0.00262428	3.01986E-11	3.01986E-11	4.68563E-08	6.04595E-07	1
1.20684E-12	2.17018E-06	1.15469E-12	1.11465E-12	0.333710696	0.002772825	1
3.01986E-11	3.35195E-08	3.01986E-11	3.01986E-11	3.01986E-11	3.01986E-11	1
2.9991E-11	0.00022539	3.01986E-11	3.01986E-11	0.000238848	3.01986E-11	1
3.01986E-11	3.01986E-11	3.01986E-11	3.01986E-11	3.01986E-11	3.01986E-11	1
3.01986E-11	3.01986E-11	3.01986E-11	3.01986E-11	3.01986E-11	4.07716E-11	1
0.501143668	2.92155E-09	7.69496E-08	3.01986E-11	2.0338E-09	5.60728E-05	1
0.016284809	0.000238848	0.000140669	4.44242E-07	2.19589E-07	2.02829E-07	1
3.01986E-11	0.529782491	1.84999E-08	3.01986E-11	0.079781647	0.055545693	1
0.610007552	0.200948897	0.020680749	3.01986E-11	0.000421751	0.051877131	1
3.01986E-11	7.19879E-05	3.01986E-11	3.01986E-11	1.85673E-09	2.77257E-05	1
3.01986E-11	0.108689773	3.01986E-11	3.01986E-11	0.001370333	0.332854692	1
3.01986E-11	0.000140669	3.01986E-11	3.01986E-11	0.190730333	0.025101283	1
3.01986E-11	0.325526587	3.01986E-11	3.01986E-11	0.014412183	0.750587232	1
3.01986E-11	5.96731E-09	3.01986E-11	3.01986E-11	2.00229E-06	0.009068761	1
3.01986E-11	3.80526E-07	3.01986E-11	3.01986E-11	0.00033679	0.149448706	1

The results show that, in most cases, ISO exhibits a statistically significant difference compared to the six competing algorithms.

In conclusion, the experimental results demonstrate that the ISO algorithm consistently outperforms other algorithms on the CEC-2011 benchmark functions, particularly for F1, F2, F8, F10, F11, F12, F17, F21, and F22. In these cases, ISO not only achieves superior performance but does so with fewer iterations, highlighting its efficiency in both convergence speed and solution quality. This indicates that the ISO algorithm is particularly effective in solving these functions, requiring fewer computational resources to reach optimal or near-optimal solutions. Figure 10 presents the convergence curves for the selected functions, providing a clear visual representation of ISO’s superior performance relative to other algorithms. Additionally, Table 6 presents the detailed performance results of different algorithms on the CEC-2011 benchmark functions, including key metrics such as mean values and standard deviations. These results further demonstrate the comparative advantages of ISO over the other algorithms. Table 6 summarizes the results of the Wilcoxon rank-sum tests, showing that the performance differences between ISO and the other algorithms are statistically significant. Specifically, the p-values for these tests are below the 5% significance threshold, confirming that ISO exhibits a statistically significant performance advantage over the competing algorithms across most of the test functions.

### CEC-2017 benchmark function test

**Fig. 11:**
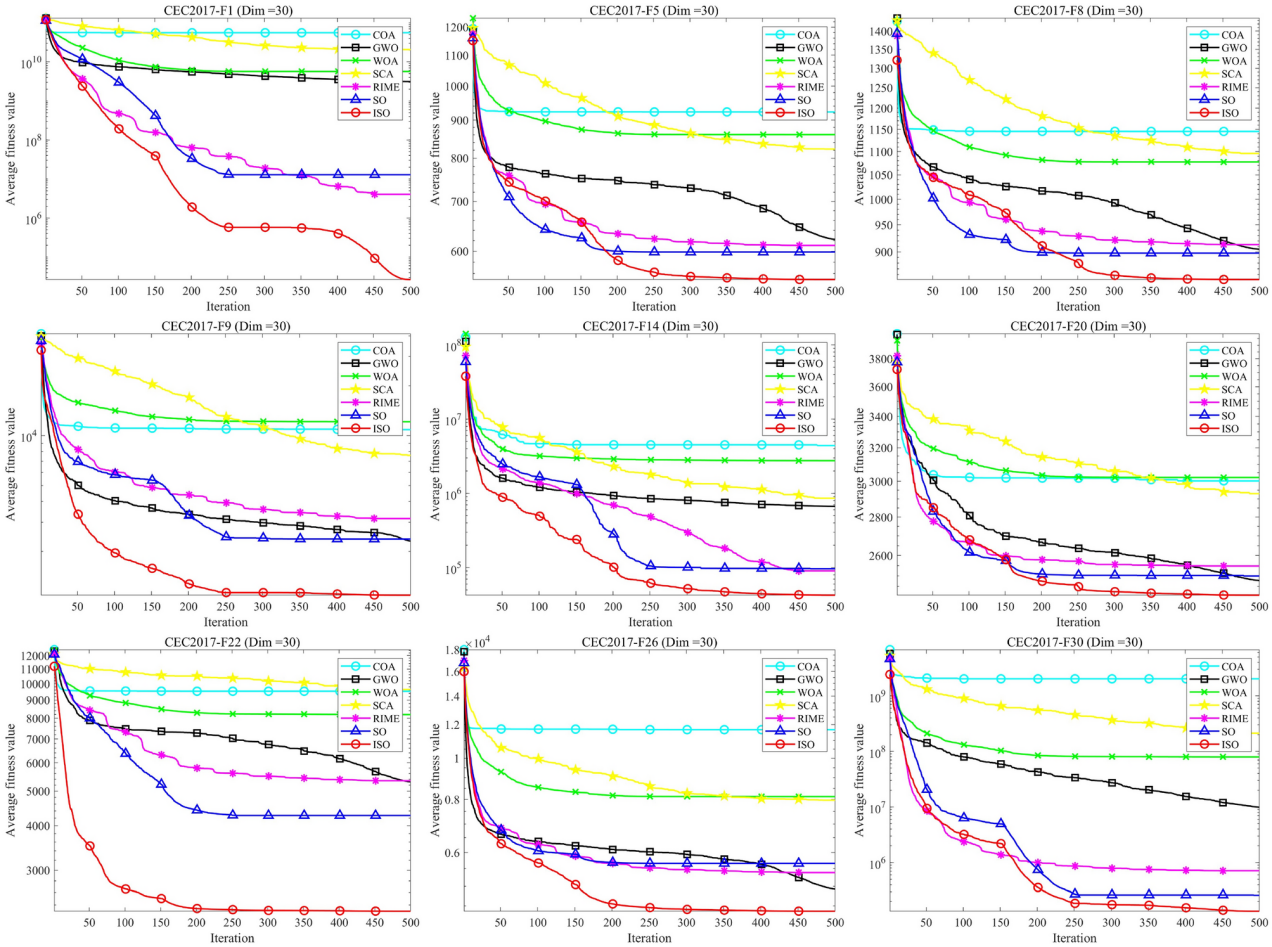
The experimental results of CEC-2017 benchmark functions (Dim=30). In most cases, ISO exhibits faster convergence and superior capability in finding the optimal values compared to the six competitive algorithms across the CEC-2017 benchmark functions (Dim=30).

**Fig. 12:**
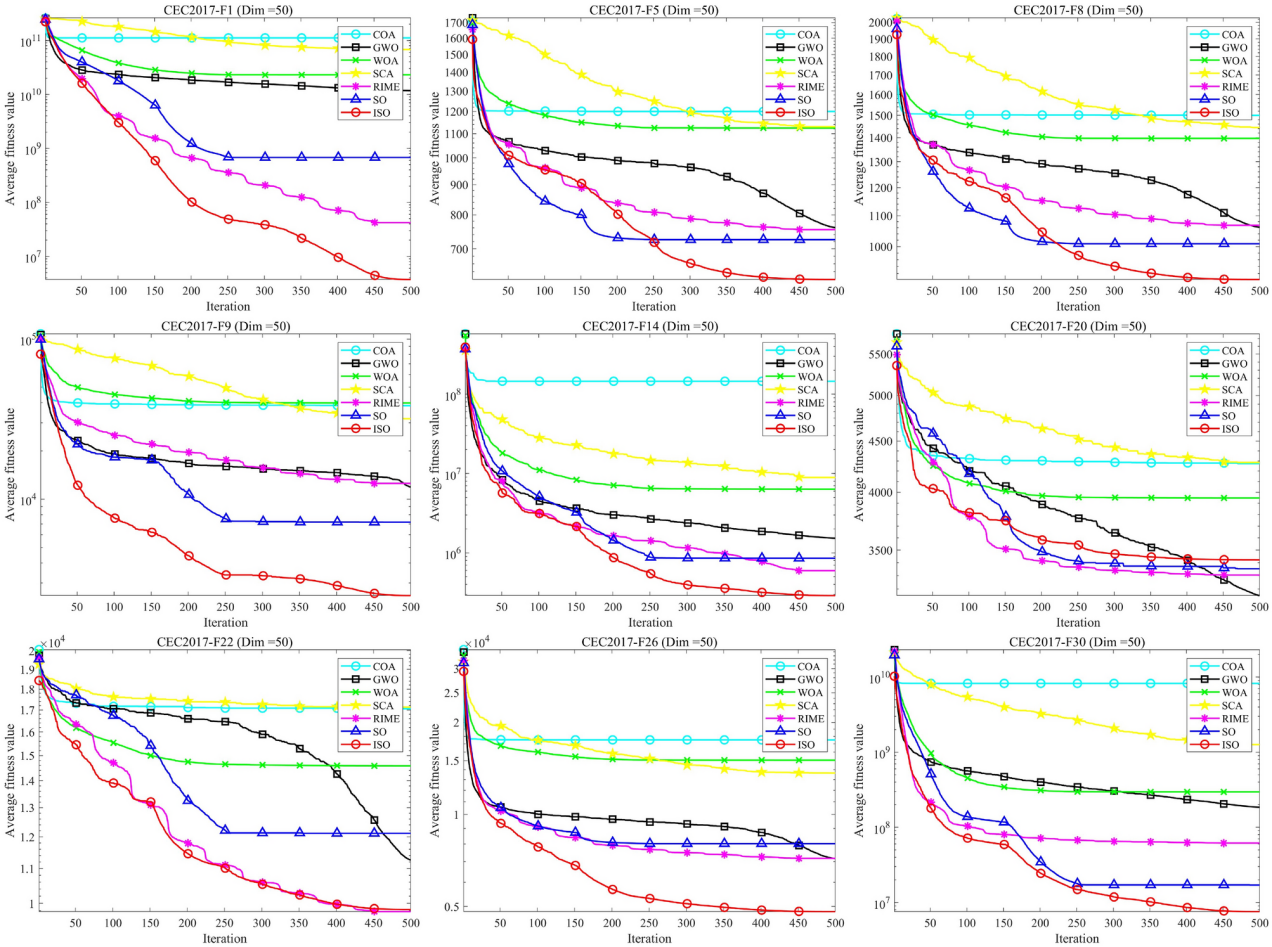
The experimental results of CEC-2017 benchmark functions (Dim=50). In most cases, ISO exhibits faster convergence and superior capability in finding the optimal values compared to the six competitive algorithms across the CEC-2017 benchmark functions (Dim=50).

**Fig. 13:**
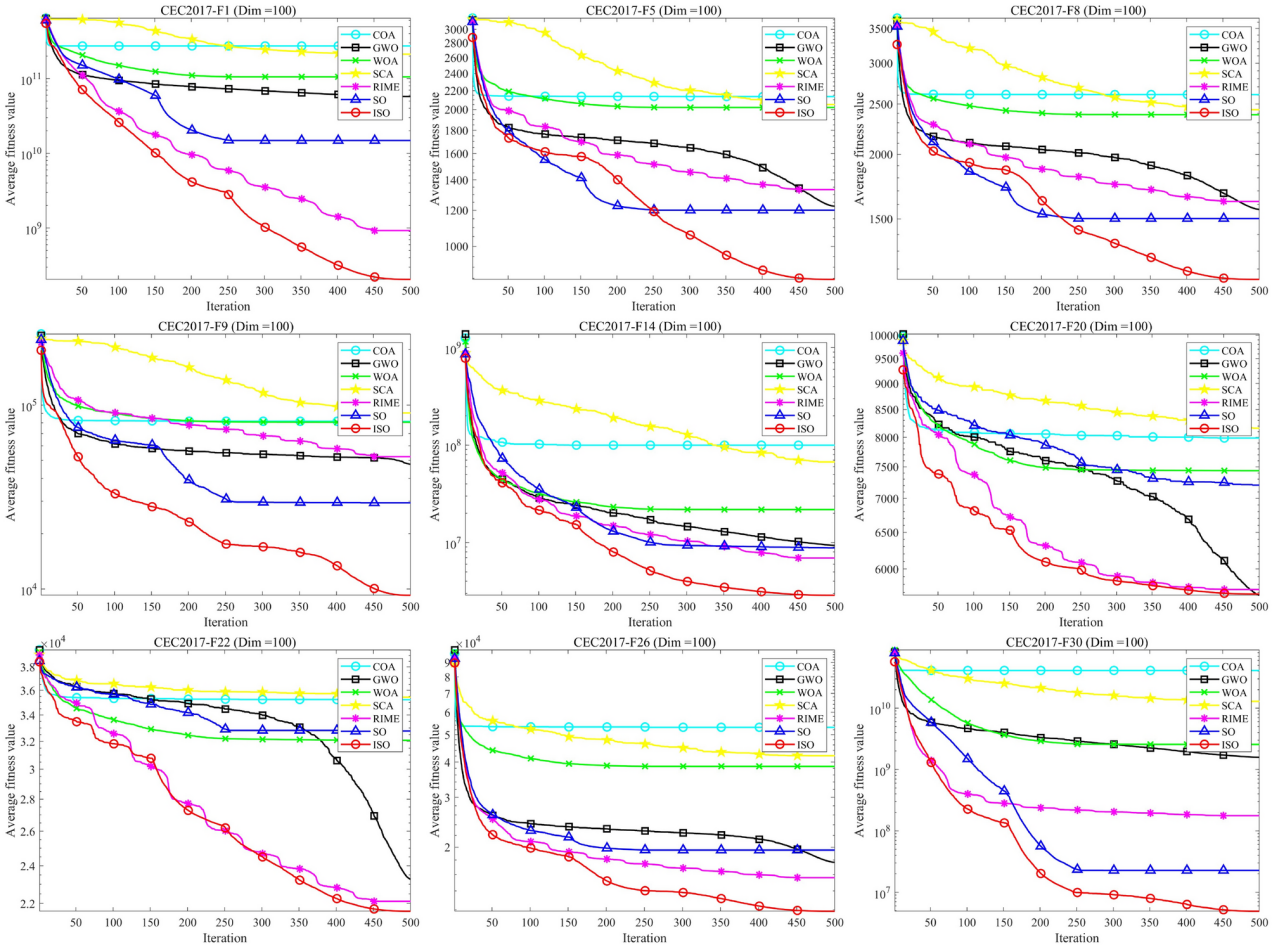
The experimental results of CEC-2017 benchmark functions (Dim=100). In most cases, ISO exhibits faster convergence and superior capability in finding the optimal values compared to the six competitive algorithms across the CEC-2017 benchmark functions (Dim=100).

Table 8 provides detailed information on the CEC-2017 benchmark functions. Figures 11, 12, and 13 present the convergence curves of the ISO algorithm and six competing algorithms when solving the 30-dimensional, 50-dimensional, and 100-dimensional CEC-2017 benchmark functions, respectively. The performance results, including the average values and standard deviations for ISO and the six competing algorithms across the 30-dimensional, 50-dimensional, and 100-dimensional CEC-2017 benchmark functions, are summarized in Tables 9, 10, and 11. The results of the rank-sum tests are shown in Tables 12, 13 and 14, providing further statistical analysis of the algorithms’ performance.

**Table 8:** CEC-2017 test function.

Class	ID	CEC2017 Function name	Rang	$f_{min}$
Unimodal	C01	Shifted and Rotated Bent Cigar Function	$[-100,100]^D$	100
	C02	Shifted and Rotated Sum of Different Power Function	$[-100,100]^D$	200
	C03	Shifted and Rotated Zakharov Function	$[-100,100]^D$	300
Multimodal	C04	Shifted and Rotated Rosenbrock’s Function	$[-100,100]^D$	400
	C05	Shifted and Rotated Rastrigin’s Function	$[-100,100]^D$	500
	C06	Shifted and Rotated Expanded Scaffer’s F6 Function	$[-100,100]^D$	600
	C07	Shifted and Rotated Lunacek Bi_Rastrigin Function	$[-100,100]^D$	700
	C08	Shifted and Rotated Non-Continuous Rastrigin’s Function	$[-100,100]^D$	800
	C09	Shifted and Rotated Levy Function	$[-100,100]^D$	900
	C10	Shifted and Rotated Schwefel’s Function	$[-100,100]^D$	1000
Hybrid	C11	Hybrid Function 1 (N=3)	$[-100,100]^D$	1100
	C12	Hybrid Function 2 (N=3)	$[-100,100]^D$	1200
	C13	Hybrid Function 3 (N=3)	$[-100,100]^D$	1300
	C14	Hybrid Function 4 (N=4)	$[-100,100]^D$	1400
	C15	Hybrid Function 5 (N=4)	$[-100,100]^D$	1500
	C16	Hybrid Function 6 (N=4)	$[-100,100]^D$	1600
	C17	Hybrid Function 6 (N=5)	$[-100,100]^D$	1700
	C18	Hybrid Function 6 (N=5)	$[-100,100]^D$	1800
	C19	Hybrid Function 6 (N=5)	$[-100,100]^D$	1900
	C20	Hybrid Function 6 (N=6)	$[-100,100]^D$	2000
Composition	C21	Composition Function 1 (N=3)	$[-100,100]^D$	2100
	C22	Composition Function 2 (N=3)	$[-100,100]^D$	2200
	C23	Composition Function 3 (N=4)	$[-100,100]^D$	2300
	C24	Composition Function 4 (N=4)	$[-100,100]^D$	2400
	C25	Composition Function 5 (N=5)	$[-100,100]^D$	2500
	C26	Composition Function 6 (N=5)	$[-100,100]^D$	2600
	C27	Composition Function 7 (N=6)	$[-100,100]^D$	2700
	C28	Composition Function 8 (N=6)	$[-100,100]^D$	2800
	C29	Composition Function 9 (N=3)	$[-100,100]^D$	2900
	C30	Composition Function 10 (N=3)	$[-100,100]^D$	3000

**Table 9:** Experimental results of seven different algorithms on the CEC-2017 benchmark functions (Dim=30), presenting the average and standard deviation for each function run by the seven algorithms.

Metric	COA	GWO	WOA	SCA	RIME	SO	ISO
Ave	55890532489	3135726382	5696722355	20738435576	4067641.28	12890208.63	26583.81954
Std	9480369921	2575297441	2261328885	3928589989	2265052.11	27629774.99	22847.3229
Ave	3.77564E+49	5.07008E+31	4.72743E+36	7.69387E+38	8.44502E+17	8.17103E+26	3.65405E+17
Std	1.69221E+50	1.36778E+32	2.51792E+37	3.13644E+39	2.72596E+18	3.55503E+27	3.56073E+17
Ave	83037.47777	60988.68827	276623.0604	84451.73653	50494.23183	68386.40049	20912.20585
Std	6561.570322	14367.54251	66831.35137	18034.75803	20215.71831	10844.66246	5073.263199
Ave	15043.1082	636.0128759	1227.25759	3207.666242	521.3446447	555.9448523	513.1164565
Std	2729.546842	78.83717909	274.544692	1005.010319	25.97473353	33.65567997	22.51778982
Ave	922.9318936	622.3790328	860.4780634	822.7601469	610.7404783	598.3310121	549.635476
Std	28.4930464	39.41039942	59.90719888	24.25919206	28.94331208	19.17682916	10.67071754
Ave	689.1553347	613.6503448	686.8388919	665.557909	614.3031212	619.5407873	603.560839
Std	7.375624001	3.894430389	12.50648998	8.234407661	7.629031343	7.92434797	2.562433931
Ave	1421.035795	898.0493454	1301.69803	1261.790241	868.4951217	905.6726291	808.0286279
Std	52.21359176	56.40792739	87.16364515	62.04657048	39.35444662	50.09263559	26.5321499
Ave	1145.445794	904.857897	1077.593725	1095.9362	913.2668133	897.8338129	851.5781449
Std	22.73935219	26.11503174	52.08348551	25.07728933	27.4904428	22.61932171	25.01048444
Ave	10870.18998	2307.157079	12142.46216	7603.953612	3159.334399	2377.034736	1089.157432
Std	1353.121902	941.2388309	4359.158519	1111.102864	1362.721031	873.6392134	245.8199564
Ave	8891.591427	4890.474632	7651.424327	8898.332892	4737.887523	4470.941519	5198.613497
Std	512.2526502	1278.721232	644.497836	352.4950229	759.8445093	879.742462	668.1387385
Ave	9334.201157	2762.851635	9502.877789	3884.846626	1327.363662	1417.784919	1271.557086
Std	1980.359024	1323.505181	3557.397281	990.2145423	53.51905581	74.65009872	62.35432764
Ave	14258918410	166314993.9	523863513	2422785117	17927546.53	2864544.062	1710265.154
Std	3245264612	381106960.7	441946808.3	703006615.4	17692984.7	2099724.997	1330282.315
Ave	10166924314	8857699.474	16184692.05	1146618149	181512.1608	48131.65182	20718.13672
Std	5304547760	24150262.62	16844004.08	541765713	201278.3311	38337.53581	14662.02796
Ave	4404480.696	667009.2132	2754377.022	861470.1783	90316.51632	96441.91244	42513.07902
Std	2625729.865	807789.578	2977449.786	650862.0955	49117.53181	119691.9571	47029.47364
Ave	671311880.1	864113.4053	3886411.662	48863677.05	19588.79875	16370.08989	9460.062306
Std	473966501.1	2409555.436	5815342.266	46684303.63	12837.79958	11548.35522	13264.87516
Ave	6093.348408	2771.472803	4263.95777	4107.145353	2829.099562	2541.844627	2476.25451
Std	1250.230117	467.5381831	634.4303614	256.4895198	272.0177573	296.7545065	321.5740422
Ave	4780.548545	2097.086845	2865.738435	2881.365543	2244.636601	2280.94047	2078.706297
Std	2701.317771	181.1973378	312.4398381	164.148377	217.4349225	189.7570725	173.0056261
Ave	47374509.57	1660487.732	15622653.27	14484030.84	1970565.476	952289.901	607891.714
Std	33812404.17	1772190.353	27933073.41	9753091.673	1940765.721	854615.7441	927298.3926
Ave	708371639.2	1420181.465	30159272.63	102221561.1	16175.37454	14652.07984	9205.589656
Std	377236881.3	3840397.031	19543628.88	68898354.11	16527.60334	13610.1277	6339.380242
Ave	3002.558789	2477.609893	3022.310892	2929.49551	2547.975296	2499.698846	2408.889621
Std	185.1168979	195.3529466	224.897809	161.278453	193.4120196	150.3015253	165.8473769
Ave	2739.735537	2401.915464	2625.944072	2607.989537	2425.417318	2398.954433	2362.493672
Std	55.40715412	30.1698233	62.05291632	26.28058678	28.46078262	18.69142471	24.94203992
Ave	9527.398572	5312.031558	8196.365692	9642.068674	5348.34809	4271.543955	2306.644793
Std	1081.794649	2391.008099	1493.915868	1566.997501	1496.08022	1899.399412	5.676305176
Ave	3623.036818	2786.698519	3159.55692	3082.371813	2793.358902	2806.136892	2720.346172
Std	155.1686311	47.69381921	109.5423638	43.88823014	34.6575252	44.973745	20.92039453
Ave	3847.390488	2971.668223	3307.378772	3254.169503	2948.348116	2957.094298	2902.252735
Std	163.4304311	70.79429317	76.45789601	41.78240797	37.53339709	32.40810309	40.4174322
Ave	5239.029575	3015.733258	3259.166628	3560.098646	2919.132699	2943.908076	2901.82976
Std	542.8125046	66.94753854	106.6804589	147.8766843	21.56062627	38.5168293	17.14488183
Ave	11674.14299	4925.607177	8123.278283	7968.186076	5384.189708	5656.922604	4372.11918
Std	713.293735	499.2855442	802.6616574	422.201936	451.3703132	468.6619561	516.1624913
Ave	4492.344458	3268.714229	3542.076288	3562.954487	3245.726688	3301.45516	3263.508299
Std	359.6101635	30.31973212	212.4822063	77.41001784	21.26560197	35.19221028	19.85223145
Ave	7485.378968	3467.298047	4021.734277	4510.815744	3289.056078	3365.769974	3268.906582
Std	709.0054651	67.61174291	591.0079641	338.6748304	24.53133684	67.79696305	39.95838269
Ave	9224.163821	3952.935839	5632.467335	5298.069689	4090.539912	4114.791496	3830.110578
Std	2221.024058	203.9081825	772.6861281	318.4364962	218.5686453	210.9977586	235.9389158
Ave	2023286684	9909713.457	79476238.3	212679260.5	711872.3461	259693.0747	134496.8666
Std	1059737665	8412978.164	54349765.11	91127983.46	632850.7563	253351.3988	153738.5423

**Table 10:** Experimental results of seven different algorithms on the CEC-2017 benchmark functions (Dim=50), presenting the average and standard deviation for each function run by the seven algorithms.

Metric	COA	GWO	WOA	SCA	RIME	SO	ISO
Ave	1.11345E+11	11753851342	23057266520	67960136527	42098885.56	677981970.1	3730300.873
Std	6929973954	5134681289	5307694965	7556815764	18624924.54	364228864.7	5256631.135
Ave	1.24178E+85	6.2681E+59	2.12781E+80	3.18282E+72	2.33616E+42	1.59671E+56	1.96886E+47
Std	6.40162E+85	3.42674E+60	1.04659E+81	1.68504E+73	8.32604E+42	8.37789E+56	9.54718E+47
Ave	205816.9519	167187.9458	351051.7457	212514.5764	219794.2329	159085.2935	152544.1462
Std	25262.51787	26259.63303	153239.5094	36814.40412	42313.25866	13171.29522	34228.713
Ave	38642.9732	1412.403028	5114.147064	13908.66878	667.4337153	913.9419695	629.1428534
Std	5833.677948	575.7464054	1424.554524	2809.033388	65.03812543	96.59371938	44.43318779
Ave	1199.90785	760.8935907	1124.050705	1130.118687	754.8751625	725.5435091	620.7013599
Std	29.55096171	30.10490073	75.52544191	35.04379082	47.91027095	40.59997471	41.88391162
Ave	702.6158735	624.1073499	698.2426029	684.3237605	630.6272758	631.8467341	608.9742288
Std	3.776063618	5.078740666	11.42903104	7.030894768	7.809657701	5.395459463	3.896330496
Ave	2050.046112	1146.858325	1882.992621	1891.159366	1110.492418	1200.524669	934.3763755
Std	51.42774373	72.44627143	85.73642155	135.3991395	49.6656864	54.47143574	39.4698516
Ave	1499.876607	1061.535421	1397.072468	1444.71286	1067.758936	1008.664911	903.1646517
Std	31.13549764	42.33424559	60.01313391	34.2763513	61.82240886	28.61895727	23.21284527
Ave	38374.08865	11906.01623	39892.79051	31783.17462	12556.54552	7189.249254	2501.224149
Std	3498.523919	5156.790971	10824.87117	4054.447668	6409.233123	2696.425388	927.5237836
Ave	15446.22673	8104.247024	13056.28108	15556.257	8250.579389	9703.722374	7960.01392
Std	504.6855635	1356.646896	1058.737841	451.4878604	870.0026449	2578.498664	990.9742726
Ave	26342.70793	8170.732688	8849.777877	13612.08887	1768.212942	3695.151822	1433.5662
Std	2744.131699	2225.493719	1926.434581	2836.573929	145.5727295	1210.086137	106.3312179
Ave	86728652805	1216457461	4392237070	22011909534	157258266.1	58300200.49	13670272
Std	15654059401	1095397368	2304877632	4613199636	81144463.51	41025061.93	10256365.21
Ave	52492570165	474621699.5	620548244.3	6326304193	464049.6175	278555.8998	41166.41787
Std	13707415781	880680115.4	347320466.4	2503047653	198970.1205	220047.2011	18362.13258
Ave	145600304.6	1528043.157	6346540.097	8910560.622	596789.0109	855517.5322	289394.2892
Std	104423794	1194330.678	4421370.899	5517895.821	481020.4495	938844.5315	307668.5818
Ave	8861107230	57487508.94	78787225.83	1184118625	112842.2311	41042.05549	18744.75583
Std	3360098983	204284335.8	97769770.9	513216498.4	59529.47041	35006.7346	8722.551969
Ave	9777.999603	3646.534171	6572.856904	6452.894599	3823.778708	3411.144192	3587.00943
Std	1183.172702	565.603842	883.6726226	306.6057632	397.0476994	364.8179181	543.0810955
Ave	12697.59678	3121.86095	4896.915815	5153.611304	3339.86451	3279.964492	3091.834628
Std	8299.63065	297.6413311	713.7068361	403.5148043	320.4424567	329.511859	480.1532932
Ave	227977212.6	12482765.29	59235947.17	56485444.5	5994130.348	4706459.755	2349681.958
Std	74331812.4	13968794.45	45105566.02	22927182.75	4420398.931	4304806.168	1511426.088
Ave	4480653902	11168491.12	21543754.34	659185328.1	454972.5703	87035.48115	21993.51857
Std	1821936078	22488227.84	18624125.99	366471660.3	581538.5269	110214.1807	12531.81045
Ave	4272.721938	3152.391436	3947.0357	4284.146782	3305.238122	3353.355211	3422.918644
Std	221.6611093	373.7959816	398.7377284	213.1530985	284.1286215	370.2875696	270.9238047
Ave	3285.703506	2558.851265	3067.363934	2975.339867	2563.098322	2519.263985	2406.349684
Std	115.3995017	56.83777376	101.4317682	50.17701648	40.06751283	30.67966117	21.51747273
Ave	17041.22579	11261.70252	14582.43234	17128.98183	9776.730218	12116.99745	9832.03828
Std	490.7008567	2946.884544	1136.847892	429.9568605	813.5507463	2398.999139	2575.528899
Ave	4621.032675	3067.301381	3826.060442	3712.665716	3055.222335	3106.819994	2896.945954
Std	212.5746997	90.9378622	183.5871782	62.82632073	70.60863206	57.93702588	44.87355612
Ave	4816.879362	3213.948527	3893.062297	3874.980902	3193.059425	3236.438933	3063.504663
Std	271.1429613	99.37453079	119.4642496	88.77487971	83.71539235	78.81735621	38.72377105
Ave	15958.32846	3788.303567	5182.451867	8803.055947	3151.545247	3354.781052	3109.171183
Std	1390.076959	300.7889794	599.4631248	1246.365955	51.18022115	121.5360353	44.22264033
Ave	17525.63269	7171.550084	15045.31806	13658.68489	7182.48646	8022.378875	4812.506165
Std	656.8159095	694.868042	1720.137552	671.2107298	567.6406542	761.4144353	869.9989901
Ave	7212.725854	3660.369679	4991.317695	4961.658315	3613.305942	3825.964324	3613.258103
Std	685.9734336	98.73817621	620.1749172	227.5971993	101.8033468	124.4891612	65.48559899
Ave	13838.67237	4557.441528	6008.101212	8977.801516	3469.305888	4546.11584	3452.626601
Std	1815.468996	416.4206302	590.9032101	1095.85919	53.45528747	598.6974852	93.65791711
Ave	100729.4024	4888.683728	9213.745742	9436.646997	5121.824383	5078.624746	4315.195466
Std	91353.64361	371.4357939	1812.989605	1584.752174	371.1678651	363.8174954	332.1396749
Ave	8238095544	185190884.3	296397633.5	1263556030	61607485.89	17048297.66	7561761.371
Std	2777364370	55303081.44	135035529.1	458304202.6	22772014.72	11704028.75	2259699.95

**Table 11:** Experimental results of seven different algorithms on the CEC-2017 benchmark functions (Dim=100), presenting the average and standard deviation for each function run by the seven algorithms.

Metric	COA	GWO	WOA	SCA	RIME	SO	ISO
Ave	1.11345E+11	11753851342	23057266520	67960136527	42098885.56	677981970.1	3730300.873
Std	6929973954	5134681289	5307694965	7556815764	18624924.54	364228864.7	5256631.135
Ave	1.24178E+85	6.2681E+59	2.12781E+80	3.18282E+72	2.33616E+42	1.59671E+56	1.96886E+47
Std	6.40162E+85	3.42674E+60	1.04659E+81	1.68504E+73	8.32604E+42	8.37789E+56	9.54718E+47
Ave	205816.9519	167187.9458	351051.7457	212514.5764	219794.2329	159085.2935	152544.1462
Std	25262.51787	26259.63303	153239.5094	36814.40412	42313.25866	13171.29522	34228.713
Ave	38642.9732	1412.403028	5114.147064	13908.66878	667.4337153	913.9419695	629.1428534
Std	5833.677948	575.7464054	1424.554524	2809.033388	65.03812543	96.59371938	44.43318779
Ave	1199.90785	760.8935907	1124.050705	1130.118687	754.8751625	725.5435091	620.7013599
Std	29.55096171	30.10490073	75.52544191	35.04379082	47.91027095	40.59997471	41.88391162
Ave	702.6158735	624.1073499	698.2426029	684.3237605	630.6272758	631.8467341	608.9742288
Std	3.776063618	5.078740666	11.42903104	7.030894768	7.809657701	5.395459463	3.896330496
Ave	2050.046112	1146.858325	1882.992621	1891.159366	1110.492418	1200.524669	934.3763755
Std	51.42774373	72.44627143	85.73642155	135.3991395	49.6656864	54.47143574	39.4698516
Ave	1499.876607	1061.535421	1397.072468	1444.71286	1067.758936	1008.664911	903.1646517
Std	31.13549764	42.33424559	60.01313391	34.2763513	61.82240886	28.61895727	23.21284527
Ave	38374.08865	11906.01623	39892.79051	31783.17462	12556.54552	7189.249254	2501.224149
Std	3498.523919	5156.790971	10824.87117	4054.447668	6409.233123	2696.425388	927.5237836
Ave	15446.22673	8104.247024	13056.28108	15556.257	8250.579389	9703.722374	7960.01392
Std	504.6855635	1356.646896	1058.737841	451.4878604	870.0026449	2578.498664	990.9742726
Ave	26342.70793	8170.732688	8849.777877	13612.08887	1768.212942	3695.151822	1433.5662
Std	2744.131699	2225.493719	1926.434581	2836.573929	145.5727295	1210.086137	106.3312179
Ave	86728652805	1216457461	4392237070	22011909534	157258266.1	58300200.49	13670272
Std	15654059401	1095397368	2304877632	4613199636	81144463.51	41025061.93	10256365.21
Ave	52492570165	474621699.5	620548244.3	6326304193	464049.6175	278555.8998	41166.41787
Std	13707415781	880680115.4	347320466.4	2503047653	198970.1205	220047.2011	18362.13258
Ave	145600304.6	1528043.157	6346540.097	8910560.622	596789.0109	855517.5322	289394.2892
Std	104423794	1194330.678	4421370.899	5517895.821	481020.4495	938844.5315	307668.5818
Ave	8861107230	57487508.94	78787225.83	1184118625	112842.2311	41042.05549	18744.75583
Std	3360098983	204284335.8	97769770.9	513216498.4	59529.47041	35006.7346	8722.551969
Ave	9777.999603	3646.534171	6572.856904	6452.894599	3823.778708	3411.144192	3587.00943
Std	1183.172702	565.603842	883.6726226	306.6057632	397.0476994	364.8179181	543.0810955
Ave	12697.59678	3121.86095	4896.915815	5153.611304	3339.86451	3279.964492	3091.834628
Std	8299.63065	297.6413311	713.7068361	403.5148043	320.4424567	329.511859	480.1532932
Ave	227977212.6	12482765.29	59235947.17	56485444.5	5994130.348	4706459.755	2349681.958
Std	74331812.4	13968794.45	45105566.02	22927182.75	4420398.931	4304806.168	1511426.088
Ave	4480653902	11168491.12	21543754.34	659185328.1	454972.5703	87035.48115	21993.51857
Std	1821936078	22488227.84	18624125.99	366471660.3	581538.5269	110214.1807	12531.81045
Ave	4272.721938	3152.391436	3947.0357	4284.146782	3305.238122	3353.355211	3422.918644
Std	221.6611093	373.7959816	398.7377284	213.1530985	284.1286215	370.2875696	270.9238047
Ave	3285.703506	2558.851265	3067.363934	2975.339867	2563.098322	2519.263985	2406.349684
Std	115.3995017	56.83777376	101.4317682	50.17701648	40.06751283	30.67966117	21.51747273
Ave	17041.22579	11261.70252	14582.43234	17128.98183	9776.730218	12116.99745	9832.03828
Std	490.7008567	2946.884544	1136.847892	429.9568605	813.5507463	2398.999139	2575.528899
Ave	4621.032675	3067.301381	3826.060442	3712.665716	3055.222335	3106.819994	2896.945954
Std	212.5746997	90.9378622	183.5871782	62.82632073	70.60863206	57.93702588	44.87355612
Ave	4816.879362	3213.948527	3893.062297	3874.980902	3193.059425	3236.438933	3063.504663
Std	271.1429613	99.37453079	119.4642496	88.77487971	83.71539235	78.81735621	38.72377105
Ave	15958.32846	3788.303567	5182.451867	8803.055947	3151.545247	3354.781052	3109.171183
Std	1390.076959	300.7889794	599.4631248	1246.365955	51.18022115	121.5360353	44.22264033
Ave	17525.63269	7171.550084	15045.31806	13658.68489	7182.48646	8022.378875	4812.506165
Std	656.8159095	694.868042	1720.137552	671.2107298	567.6406542	761.4144353	869.9989901
Ave	7212.725854	3660.369679	4991.317695	4961.658315	3613.305942	3825.964324	3613.258103
Std	685.9734336	98.73817621	620.1749172	227.5971993	101.8033468	124.4891612	65.48559899
Ave	13838.67237	4557.441528	6008.101212	8977.801516	3469.305888	4546.11584	3452.626601
Std	1815.468996	416.4206302	590.9032101	1095.85919	53.45528747	598.6974852	93.65791711
Ave	100729.4024	4888.683728	9213.745742	9436.646997	5121.824383	5078.624746	4315.195466
Std	91353.64361	371.4357939	1812.989605	1584.752174	371.1678651	363.8174954	332.1396749
Ave	8238095544	185190884.3	296397633.5	1263556030	61607485.89	17048297.66	7561761.371
Std	2777364370	55303081.44	135035529.1	458304202.6	22772014.72	11704028.75	2259699.95

**Table 12:** Wilcoxon rank-sum test results for the performance of seven different algorithms on CEC-2017 benchmark functions (Dim=30).

COA	GWO	WOA	SCA	RIME	SO	ISO
3.01986E-11	3.01986E-11	3.01986E-11	3.01986E-11	3.01986E-11	3.01986E-11	1
3.01986E-11	3.01986E-11	3.01986E-11	3.01986E-11	0.074827008	3.01986E-11	1
3.01986E-11	3.01986E-11	3.01986E-11	3.01986E-11	2.87158E-10	3.01986E-11	1
3.01986E-11	1.20567E-10	3.01986E-11	3.01986E-11	0.118817344	8.1975E-07	1
3.01986E-11	4.07716E-11	3.01986E-11	3.01986E-11	9.91863E-11	1.20567E-10	1
3.01986E-11	1.32885E-10	3.01986E-11	3.01986E-11	3.19674E-09	9.91863E-11	1
3.01986E-11	4.57257E-09	3.01986E-11	3.01986E-11	4.31061E-08	6.12104E-10	1
3.01986E-11	9.26029E-09	3.01986E-11	3.01986E-11	3.82489E-09	2.19474E-08	1
3.01986E-11	2.66947E-09	3.01986E-11	3.01986E-11	6.69552E-11	1.46431E-10	1
3.01986E-11	0.009068761	3.01986E-11	3.01986E-11	0.011710684	0.000268057	1
3.01986E-11	3.01986E-11	3.01986E-11	3.01986E-11	0.000526404	8.48477E-09	1
3.01986E-11	3.33839E-11	3.01986E-11	3.01986E-11	2.1544E-10	0.053685253	1
3.01986E-11	3.01986E-11	3.01986E-11	3.01986E-11	9.7555E-10	7.19879E-05	1
3.01986E-11	8.84109E-07	1.95678E-10	4.07716E-11	5.26501E-05	0.01221194	1
3.01986E-11	2.87158E-10	3.01986E-11	3.01986E-11	4.35308E-05	0.000587373	1
3.01986E-11	0.016954881	3.01986E-11	3.01986E-11	3.36814E-05	0.549326784	1
3.01986E-11	0.773119942	3.33839E-11	3.01986E-11	0.003848068	0.000149316	1
3.33839E-11	0.000168132	7.38029E-10	8.99341E-11	0.000149316	0.007617064	1
3.01986E-11	3.68973E-11	3.01986E-11	3.01986E-11	0.27718919	0.122352926	1
4.50432E-11	0.264326213	1.32885E-10	6.06576E-11	0.004637118	0.087710377	1
3.01986E-11	3.01026E-07	3.01986E-11	3.01986E-11	7.11859E-09	9.06321E-08	1
3.01986E-11	3.01986E-11	3.01986E-11	3.01986E-11	1.09367E-10	3.68973E-11	1
3.01986E-11	6.72195E-10	3.01986E-11	3.01986E-11	2.60985E-10	1.41098E-09	1
3.01986E-11	3.5708E-06	3.01986E-11	3.01986E-11	1.86817E-05	1.72903E-06	1
3.01986E-11	3.68973E-11	3.01986E-11	3.01986E-11	0.000421751	4.44405E-07	1
3.01986E-11	5.09117E-06	3.01986E-11	3.01986E-11	3.19674E-09	4.97517E-11	1
3.01986E-11	0.482516904	4.50432E-11	3.01986E-11	0.00262428	9.51394E-06	1
3.01986E-11	8.15274E-11	3.01986E-11	3.01986E-11	0.000178356	1.20233E-08	1
3.01986E-11	0.040595001	3.01986E-11	3.01986E-11	0.000110577	2.43271E-05	1
3.01986E-11	3.68973E-11	3.01986E-11	3.01986E-11	3.08105E-08	0.004856016	1

The results show that, in most cases, ISO exhibits a statistically significant difference compared to the six competing algorithms.

**Table 13:** Wilcoxon rank-sum test results for the performance of seven different algorithms on CEC-2017 benchmark functions (Dim=50).

COA	GWO	WOA	SCA	RIME	SO	ISO
3.01986E-11	3.01986E-11	3.01986E-11	3.01986E-11	4.07716E-11	3.01986E-11	1
3.01986E-11	5.07231E-10	3.01986E-11	3.01986E-11	0.000356384	4.61591E-10	1
2.78287E-07	0.0351366	2.1544E-10	1.8731E-07	9.83289E-08	0.108689773	1
3.01986E-11	3.33839E-11	3.01986E-11	3.01986E-11	0.010314672	3.33839E-11	1
3.01986E-11	2.87158E-10	3.01986E-11	3.01986E-11	2.87158E-10	1.07018E-09	1
3.01986E-11	4.97517E-11	3.01986E-11	3.01986E-11	6.69552E-11	4.07716E-11	1
3.01986E-11	3.01986E-11	3.01986E-11	3.01986E-11	3.33839E-11	3.01986E-11	1
3.01986E-11	3.01986E-11	3.01986E-11	3.01986E-11	4.07716E-11	4.97517E-11	1
3.01986E-11	3.68973E-11	3.01986E-11	3.01986E-11	7.38908E-11	5.57265E-10	1
3.01986E-11	0.888302842	3.01986E-11	3.01986E-11	0.318304227	0.010314672	1
3.01986E-11	3.01986E-11	3.01986E-11	3.01986E-11	3.82016E-10	3.01986E-11	1
3.01986E-11	3.01986E-11	3.01986E-11	3.01986E-11	3.33839E-11	4.57257E-09	1
3.01986E-11	3.01986E-11	3.01986E-11	3.01986E-11	3.01986E-11	9.7555E-10	1
3.01986E-11	1.01045E-08	3.01986E-11	3.01986E-11	8.14648E-05	0.000498182	1
3.01986E-11	3.68973E-11	3.01986E-11	3.01986E-11	3.33839E-11	0.000212646	1
3.01986E-11	0.958731491	3.33839E-11	3.01986E-11	0.072445596	0.084999697	1
3.01986E-11	0.652043622	4.50432E-11	3.01986E-11	0.043583548	0.084999697	1
3.01986E-11	2.49131E-06	4.07716E-11	3.01986E-11	4.35308E-05	0.008684371	1
3.01986E-11	3.01986E-11	3.01986E-11	3.01986E-11	1.46431E-10	0.000140669	1
8.99341E-11	0.000729511	1.10772E-06	4.50432E-11	0.11536236	0.258051495	1
3.01986E-11	3.01986E-11	3.01986E-11	3.01986E-11	3.01986E-11	3.68973E-11	1
3.01986E-11	0.830255284	3.33839E-11	3.01986E-11	0.00037704	0.015014133	1
3.01986E-11	5.49405E-11	3.01986E-11	3.01986E-11	8.15274E-11	3.01986E-11	1
3.01986E-11	2.1544E-10	3.01986E-11	3.01986E-11	1.17374E-09	4.97517E-11	1
3.01986E-11	3.01986E-11	3.01986E-11	3.01986E-11	0.000526404	8.15274E-11	1
3.01986E-11	3.68973E-11	3.01986E-11	3.01986E-11	3.68973E-11	3.01986E-11	1
3.01986E-11	0.099257608	3.01986E-11	3.01986E-11	0.549326784	8.89099E-10	1
3.01986E-11	3.01986E-11	3.01986E-11	3.01986E-11	0.045146208	4.97517E-11	1
3.01986E-11	1.25408E-07	3.01986E-11	3.01986E-11	3.49711E-09	7.38029E-10	1
3.01986E-11	3.01986E-11	3.01986E-11	3.01986E-11	3.01986E-11	2.87897E-06	1

The results show that, in most cases, ISO exhibits a statistically significant difference compared to the six competing algorithms.

**Table 14:** Wilcoxon rank-sum test results for the performance of seven different algorithms on CEC-2017 benchmark functions (Dim=100).

COA	GWO	WOA	SCA	RIME	SO	ISO
3.01986E-11	3.01986E-11	3.01986E-11	3.01986E-11	1.46431E-10	3.01986E-11	1
3.01986E-11	4.61591E-10	3.01986E-11	3.01986E-11	0.001236185	3.68973E-11	1
0.347827783	0.009883401	4.07716E-11	1.09069E-05	7.77255E-09	0.297271689	1
3.01986E-11	3.01986E-11	3.01986E-11	3.01986E-11	9.26029E-09	3.01986E-11	1
3.01986E-11	3.01986E-11	3.01986E-11	3.01986E-11	3.01986E-11	3.01986E-11	1
3.01986E-11	3.01986E-11	3.01986E-11	3.01986E-11	3.01986E-11	3.01986E-11	1
3.01986E-11	3.01986E-11	3.01986E-11	3.01986E-11	3.01986E-11	3.01986E-11	1
3.01986E-11	3.01986E-11	3.01986E-11	3.01986E-11	3.01986E-11	3.01986E-11	1
3.01986E-11	3.01986E-11	3.01986E-11	3.01986E-11	3.01986E-11	3.01986E-11	1
3.01986E-11	0.057459546	3.01986E-11	3.01986E-11	3.83494E-06	3.01986E-11	1
3.01986E-11	3.33839E-11	3.01986E-11	3.01986E-11	0.706171488	3.01986E-11	1
3.01986E-11	3.01986E-11	3.01986E-11	3.01986E-11	3.01986E-11	3.01986E-11	1
3.01986E-11	3.01986E-11	3.01986E-11	3.01986E-11	3.01986E-11	3.01986E-11	1
3.01986E-11	2.60985E-10	3.68973E-11	3.01986E-11	6.2828E-06	1.01045E-08	1
3.01986E-11	3.01986E-11	3.01986E-11	3.01986E-11	3.01986E-11	2.1544E-10	1
3.01986E-11	0.001057554	3.01986E-11	3.01986E-11	1.38525E-06	0.001952677	1
3.01986E-11	0.01221194	3.01986E-11	3.01986E-11	0.000268057	0.005322078	1
3.01986E-11	0.020680749	6.06576E-11	3.01986E-11	1.35943E-07	5.09117E-06	1
3.01986E-11	3.01986E-11	3.01986E-11	3.01986E-11	3.01986E-11	2.87158E-10	1
3.01986E-11	0.180899533	1.09367E-10	3.01986E-11	0.662734758	9.7555E-10	1
3.01986E-11	3.01986E-11	3.01986E-11	3.01986E-11	3.01986E-11	3.01986E-11	1
3.01986E-11	0.994101921	3.01986E-11	3.01986E-11	0.217016825	3.01986E-11	1
3.01986E-11	4.50432E-11	3.01986E-11	3.01986E-11	3.01986E-11	3.01986E-11	1
3.01986E-11	4.50432E-11	3.01986E-11	3.01986E-11	4.1825E-09	3.01986E-11	1
3.01986E-11	3.01986E-11	3.01986E-11	3.01986E-11	2.59736E-05	3.01986E-11	1
3.01986E-11	3.01986E-11	3.01986E-11	3.01986E-11	5.49405E-11	3.01986E-11	1
3.01986E-11	3.4742E-10	3.01986E-11	3.01986E-11	3.83067E-05	1.46431E-10	1
3.01986E-11	3.01986E-11	3.01986E-11	3.01986E-11	0.000811998	3.01986E-11	1
3.01986E-11	3.01986E-11	3.01986E-11	3.01986E-11	3.01986E-11	4.97517E-11	1
3.01986E-11	3.01986E-11	3.01986E-11	3.01986E-11	3.01986E-11	8.10136E-10	1

The results show that, in most cases, ISO exhibits a statistically significant difference compared to the six competing algorithms.

The ISO algorithm was evaluated on the CEC-2017 benchmark functions across various dimensions, including 30-dimensional, 50-dimensional, and 100-dimensional problems. The experimental results demonstrate that ISO consistently exhibits superior performance in terms of both convergence speed and solution quality when compared to six competing algorithms. The convergence curves, presented in Figs. 11, 12, and 13, illustrate that ISO achieves faster convergence and more accurate solutions across all dimensions. The performance metrics, including the mean values and standard deviations provided in Tables 8, 12 and 13, further confirm the stability and efficiency of the ISO algorithm. Additionally, the results of the rank-sum tests, summarized in Tables 9, 10 and 11, reveal statistically significant performance differences between ISO and the other algorithms, underscoring its superior effectiveness in solving the CEC-2017 benchmark functions. These findings highlight ISO’s robust performance and its suitability for solving high-dimensional optimization problems with enhanced efficiency.

## Engineering application

### UAV path planning (UPP)

For decades, Unmanned Aerial Vehicles (UAVs) have been recognized as one of the most challenging and promising technologies in the field of aviation. UAVs have found widespread applications in various domains, including agriculture^62^, power line inspection^63^, aerial photography^64^, and national defense^65^. Among the key technologies driving UAV development, path planning has emerged as a critical area of research, attracting significant attention from scholars worldwide^66^. UAVs, often conceptualized as particles of negligible size, must navigate the complexities of their environment efficiently. The principal objective of UAV path planning is to design an optimal flight path that minimizes overall cost, while adhering to the performance requirements of the UAV. In this problem, the maximum number of iterations is $T = 100$, and the population size is $N = 15$. In UAV path planning, the primary considerations are threat cost and fuel cost, as presented in Eq. (37).

37 $$\begin{aligned} Ft = k_1 \times F_{tc} + k_2 \times F_{fc} \end{aligned}$$

Here, *Ft* indicates the total cost, $F_{tc}$ represents the threat cost calculated using Eq. (38), $F_{fc}$ denotes the fuel cost calculated by Eq. (39), and $k_i (i = 1,2)$ are weight coefficients.

38 $$\begin{aligned} F_{tc} = \sum _{j=2}^{\text {dim}-1} \sqrt{\left( \frac{x_{end}}{j+1}\right) ^2 + (path(j) - path(j-1))^2} \end{aligned}$$

where $x_{end}$ represents the abscissa value of the flight endpoint, and *path*(*j*) represents the *j*th subpath.

39 $$\begin{aligned} F_{fc} = \sum _{j=1}^{6} F_{tc}(j) \sum _{b=1}^{6} \left( \frac{1}{\text {dis}(C_b, P_b, (j-0.1) \cdot dx, (0.9 \cdot path(j-1) + 0.1 \cdot path(j)))} \right) ^4 \end{aligned}$$

where $C_b$ is the coordinate of the *b*th threat center, $P_b$ is the threat level of the *b*th threat center, and $\text {dis}$ is the distance function between two points.

40 $$\begin{aligned} \left\{ \begin{array}{l} k_i \ge 0 \\ \sum _{i=1}^{k} k_i = 1 \end{array} \right. \end{aligned}$$

**Fig. 14:**
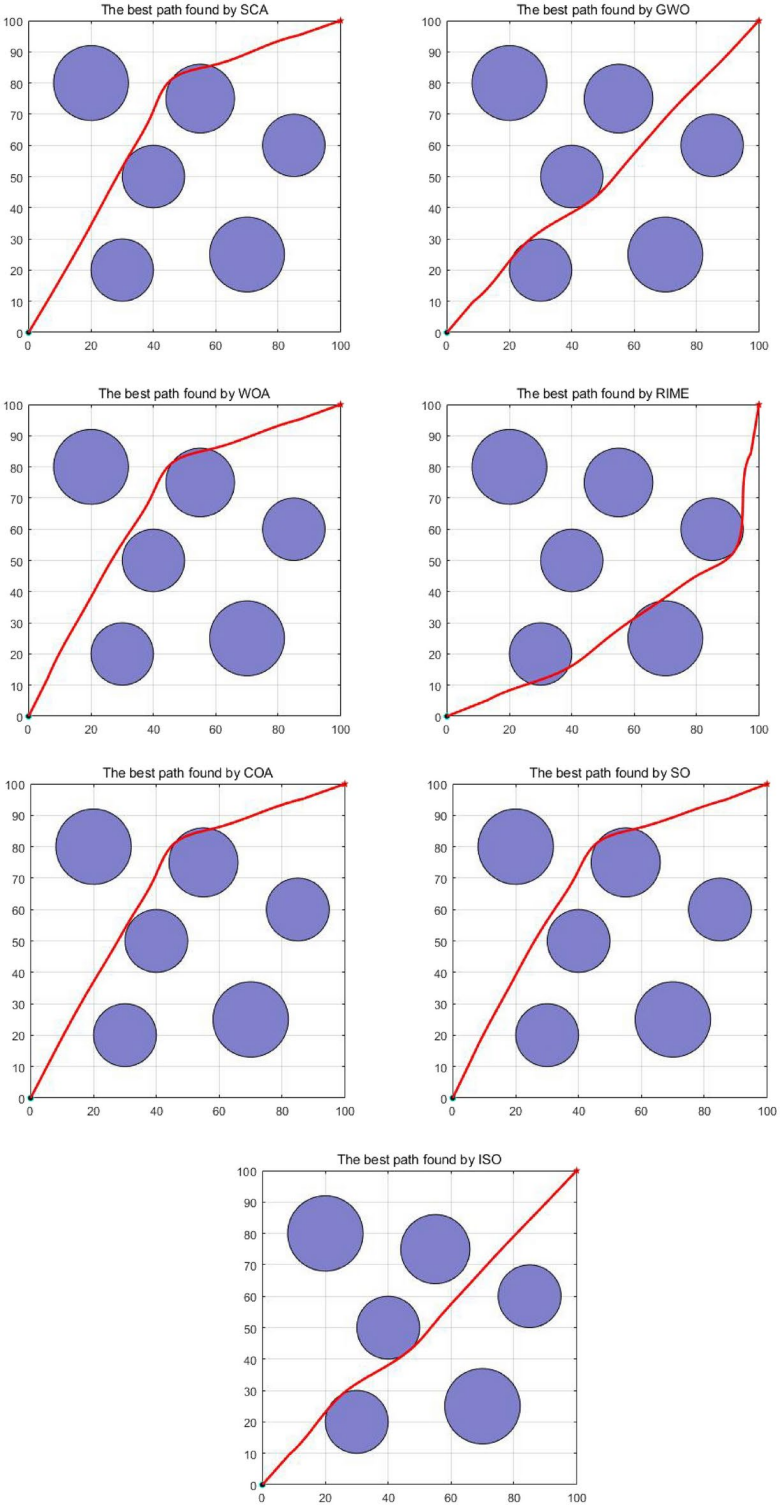
Visualization of the results for the 2D UAV path planning problem using seven different algorithms. The figure shows that the path obtained by ISO is the shortest and the smoothest.

**Fig. 15:**
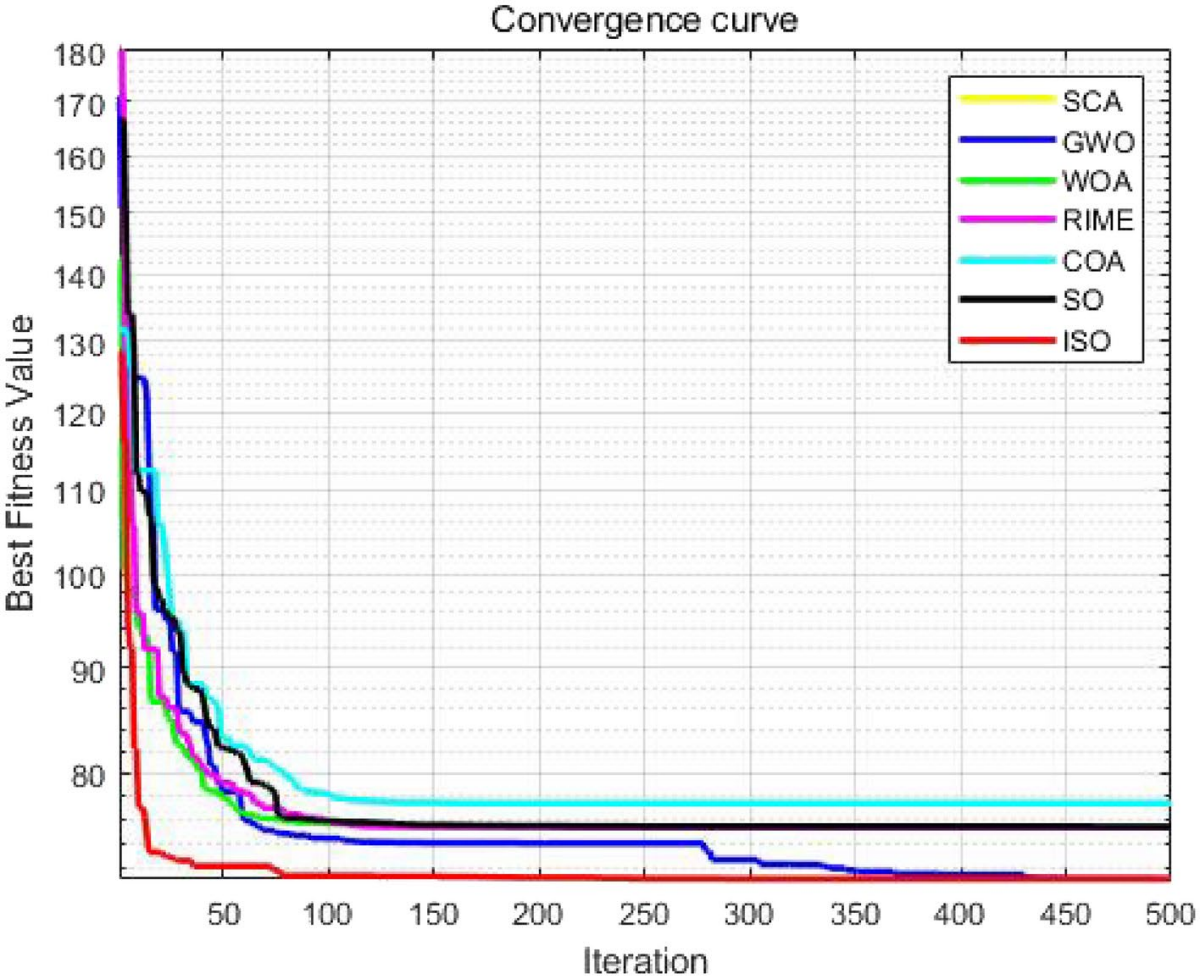
The convergence curves of seven different algorithms applied to the 2D UAV path planning problem. The figure demonstrates that ISO has the fastest convergence speed and is capable of finding the shortest path.

Figure 14 displays the visual results of different algorithms for solving UAV path planning. It is evident that the trajectory obtained by ISO is smoother. Figure 15 presents the convergence curves of various algorithms for this problem, showing that ISO achieves faster convergence and superior results compared to other algorithms. Table 15 quantitatively displays the final solution results. The results indicate that the ISO algorithm yielded the best performance, with a value of 73.52, demonstrating its superiority in optimal path planning. The SO algorithm followed closely with a value of 77.54, while the RIME algorithm produced a comparable value of 77.61. The GWO algorithm performed slightly better than both SO and RIME, achieving a value of 79.14. The SCA algorithm resulted in a value of 77.00, and the WOA algorithm produced a result of 77.72, placing them in the mid-range of performance. Overall, the ISO algorithm ranked first, outperforming the other methods in terms of path planning efficiency for UAVs.

**Table 15:** The final results for the 2D UAV path planning problem using seven different algorithms.

SCA	GWO	WOA	RIME	COA	SO	ISO
75.29	71.07	75.31	77.34	75.30	75.34	**71.05**

The table indicates that ISO achieved the best solution.

### Robot path planning (RPP)

Mobile robots play an important role in a wide range of fields such as the automation industry^67^, military security^68^, agricultural operations^69^, mining exploration^70^, and warehouse management^71^. One of their core tasks is path planning, which aims to comprehensively consider the geometric dimensions, physical limitations, and time efficiency of the robot, and plan a collision-free and optimized travel route in complex working environments^72^. Unlike motion planning, which delves into dynamic characteristics, path planning focuses more on finding a path that minimizes travel time and accurately reflects environmental features from a kinematic perspective. To achieve this goal, robots need to construct or receive spatial models of their operating environment. These models can be explicit, actively created and stored by the robots^73^, or implicit, indirectly utilized through algorithms. These spatial models can exist in various forms, including discretized representations such as grid maps, where space is uniformly divided into small blocks, or non-uniform discretization forms such as topological maps, which focus more on representing critical paths and the connection relationships between nodes. Additionally, there are continuous mapping methods that accurately describe the environment by defining a continuous coordinate sequence of polygon boundaries and paths around the boundaries. Although this method is more efficient in memory usage, in the field of robot path planning, discretized maps are favored for their ability to naturally transform into graph structures. The graph structure not only intuitively reflects environmental characteristics, but also benefits from the rich search and optimization algorithm resources in the field of graph theory, making the calculation process both efficient and concise. Therefore, although continuous mapping has its unique advantages, in practical operation, discretized maps are still the mainstream choice for robot path planning^74^. In this experiment, a robot walking on the ground is regarded as a particle of negligible size. Primary considerations encompass the intricate obstacle space and the optimization of the fitness function. The upper limit of the iteration count is set to T = 100 and the population size to N = 15. To simultaneously assess the feasibility and convergence performance of the algorithm, this experiment simulated a complex spatial environment for the robot.The detailed description is as follows: The matrix G provided undergoes a sequence of operations to produce a new matrix G. These operations are outlined as follows: The initial matrix G, with dimensions m $\times$ n, is extended by transposing it and vertically concatenating it with itself: Matrix Extension: The initial matrix $G$, sized $m \times n$, is extended by transposing and vertically concatenating it with itself:

41 $$\begin{aligned} G' = \begin{bmatrix} G^T \\ G^T \end{bmatrix} \end{aligned}$$

where $G^T$ denotes the transpose of $G$. Further Extension The resultant matrix $G'$, now sized $2m \times n$, is further extended by transposing and vertically concatenating it with itself again:

42 $$\begin{aligned} G'' = \begin{bmatrix} G'^T \\ G'^T \end{bmatrix} \end{aligned}$$

resulting in a matrix $G''$ of size $4m \times 2n$. Matrix Flipping The extended matrix $G''$ undergoes a flipping operation where the upper half rows are swapped with the corresponding lower half rows:

43 $$\begin{aligned} \forall i \in \left[ 1, \frac{\text {num}}{2}\right] , \forall j \in [1, \text {num}]: \left( \begin{array}{ll} G''(i, j)&\leftrightarrow G''(\text {num} + 1 - i, j) \end{array} \right) \end{aligned}$$

where $\text {num}$ is the total number of rows in $G''$. Through these operations, the initial matrix $G$ is transformed into the matrix $G''$, simulating the complex and challenging obstacle space We define the fitness function $f_{\text {fitness}}(x, G)$ that evaluates each path by checking if the path passes through an obstacle and by calculating the total length of the path. Starting Point End Point Definition:

The start point $S$ is fixed to the upper left corner of the map $(1, 1)$.

The end point $E$ is the lower right corner of the map $(m, n)$, where $G$ is a matrix of size $m \times n$. Path definition: A path $\textbf{route}$ consists of a start point $S$, a midpoint $x$ and an end point $E$:

44 $$\begin{aligned} \textbf{route} = [S, x, E] \end{aligned}$$

where $x$ is a series of coordinate points representing the intermediate path points from the start point to the end point.

Obstacle Checking: Each point in the path $\textbf{route}$ is checked and the number of obstacles the path passes through is calculated $n_B$:

45 $$\begin{aligned} n_B = \sum _{j=2}^{\text {dim}-1} (G(\textbf{route}(j), j) = 1) \end{aligned}$$

Path length calculation: If the path does not pass through any obstacle, i.e. $n_B = 0$, a smooth path $\textbf{path}$ is generated and the total length of the path is calculated:

46 $$\begin{aligned} f_{\text {fitness}}(x, G) = \sum _{i=1}^{|\textbf{path}|-1} \sqrt{(\textbf{path}(i + 1, 1) - \textbf{path}(i, 1))^2 + (\textbf{path}(i + 1, 2) - \textbf{path}(i, 2))^2} \end{aligned}$$

Collision penalty: If the path passes through an obstacle, i.e. $n_B > 0$, the adaptation value is the map area multiplied by the number of collisions:

47 $$\begin{aligned} f_{\text {fitness}}(x, G) = m \cdot n \cdot n_B \end{aligned}$$

The fitness function: Combining the above, the final form of the fitness function is:

48 $$\begin{aligned} \hspace{-1.3cm} f_{\text {fitness}}(x, G) = {\left\{ \begin{array}{ll} \frac{1}{m \cdot n \cdot n_B} \sum _{i=1}^{|\textbf{path}|-1} \sqrt{(\textbf{path}(i+1, 1) - \textbf{path}(i, 1))^2 + (\textbf{path}(i+1, 2) - \textbf{path}(i, 2))^2} & \text {if } n_B > 0 \\ 0 & \text {if } n_B = 0 \end{array}\right. } \end{aligned}$$

**Fig. 16:**
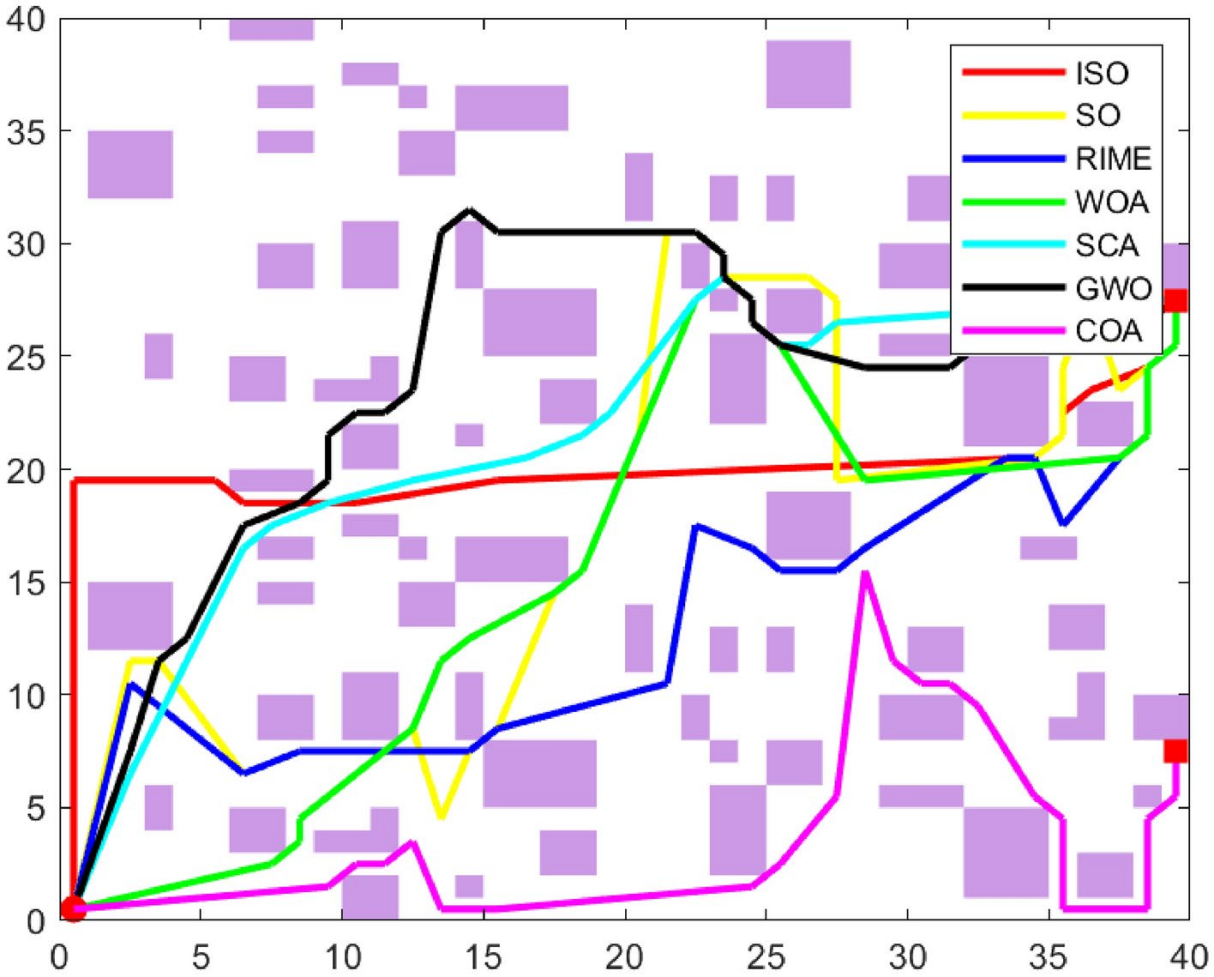
Visualization of the results for the robot path planning problem using seven different algorithms. ISO is able to obtain the shortest path.

Figure 16 presents the visualization of the results for the robot path planning problem, showing that ISO achieved the shortest path compared to the other algorithms. Moreover, The experimental results, as shown in Table 16, compare the performance of different algorithms for robot path planning. The SCA algorithm achieves an optimal value of 75.02, demonstrating its moderate performance in finding a path. The GWO algorithm performs slightly better with a value of 65.91, showing relatively efficient path planning but still not as effective as the best-performing algorithms. The WOA algorithm follows with a value of 66.43, and the RIME algorithm achieves a value of 67.96, indicating their comparable effectiveness in path planning, though they still do not reach the efficiency of the top algorithms. The COA algorithm delivers a value of 70.32, indicating better performance than some algorithms but not excelling in comparison to others like ISO. The SO algorithm, with a high value of 95.90, shows the least efficiency in optimizing the robot’s path, highlighting its relative inefficiency in this task. Finally, the ISO algorithm outperforms all other algorithms with the lowest value of 63.02, demonstrating its superior efficiency in finding the optimal path. This result confirms that ISO is the most effective solution among the algorithms tested, offering the best performance in robot path planning. Overall, the results highlight ISO as the leading algorithm for optimizing robot path planning, significantly outperforming the other methods tested.

**Table 16:** The final results of the robot path planning problem using seven different algorithms.

SCA	GWO	WOA	RIME	COA	SO	ISO
75.02	65.91	66.43	67.96	70.32	95.90	**63.02**

ISO achieved the shortest path.

###  Wireless sensor network node deployment(WSNND)

With the development of distributed environments such as grid computing^75^, peer-to-peer networks^76^ and cloud computing^77^ ,wireless sensor networks (WSNs) have become more popular than ever. WSNs represent an emerging computing and networking paradigm that can be defined as networks composed of miniature, small, inexpensive, and highly intelligent devices known as sensor nodes.^78^ In Wireless sensor network node deployment,We deploy $n$ wireless sensor nodes in a two-dimensional planar region $A$^79^. Each node can obtain its position and has an identical sensing radius $R$. The sensing radius $R$ denotes the maximum distance a sensor node can detect, meaning all points within a circular area centered at node $s_i$ with radius $R$ are covered by this node. Our objective is to optimize the distribution of nodes to maximize the proportion of the area that is covered. For this problem, set the maximum iteration count T = 500 and the population size N = 200. Let $S = \{s_1, s_2, \ldots , s_n\}$ represent all WSN nodes in region $A$, where $s_i = (x_i, y_i)$ denotes the coordinates of node $s_i$ in $A$. For any grid point $p_j$ ( $j = 1, 2, \ldots , L \times L$ ), with coordinates $(x_j, y_j)$, the distance between node $s_i$ and grid point $p_j$ is given by:

49 $$\begin{aligned} d_{ij} = \sqrt{(x_i - x_j)^2 + (y_i - y_j)^2} \end{aligned}$$

The coverage of grid point $p_j$ by node $s_i$ is described by:

50 $$\begin{aligned} C_{ij} = {\left\{ \begin{array}{ll} 1 & \text {if } d_{ij} \le R \\ 0 & \text {if } d_{ij} > R \end{array}\right. } \end{aligned}$$

Where $R$ is the sensing radius of the sensor Define an $L \times L$ matrix $M$ where each element indicates whether the corresponding grid point is covered. Initially, all elements of $M$ are 0. For each node $s_i$, compute all grid points it covers and set the corresponding elements in $M$ to 1. The coverage rate $C_s$ is defined as the ratio of covered grid points to the total number of grid points:

51 $$\begin{aligned} C_s = \frac{\sum _{i=1}^{L^2} M(i)}{L^2} \end{aligned}$$

Our optimization objective is to maximize the coverage rate $C_s$. Since $C_s$ represents the proportion of the covered area, the optimization problem can be formulated as:

52 $$\begin{aligned} \max C_s \end{aligned}$$

**Fig. 17:**
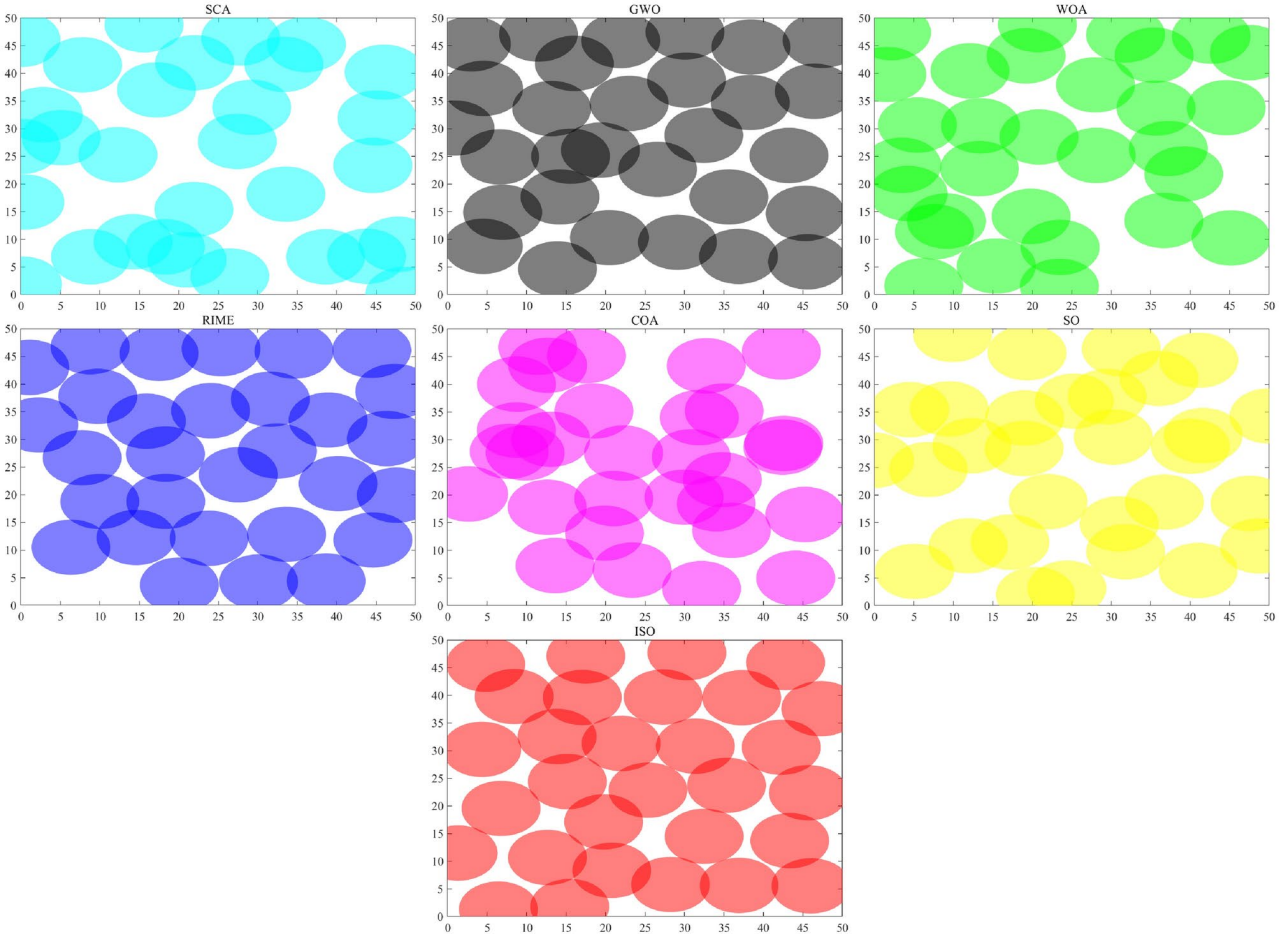
Visualization of the results for the wireless network coverage problem using seven different algorithms. The figure shows that the wireless network nodes deployed using ISO can achieve the maximum coverage area in the 2D space.

**Fig. 18:**
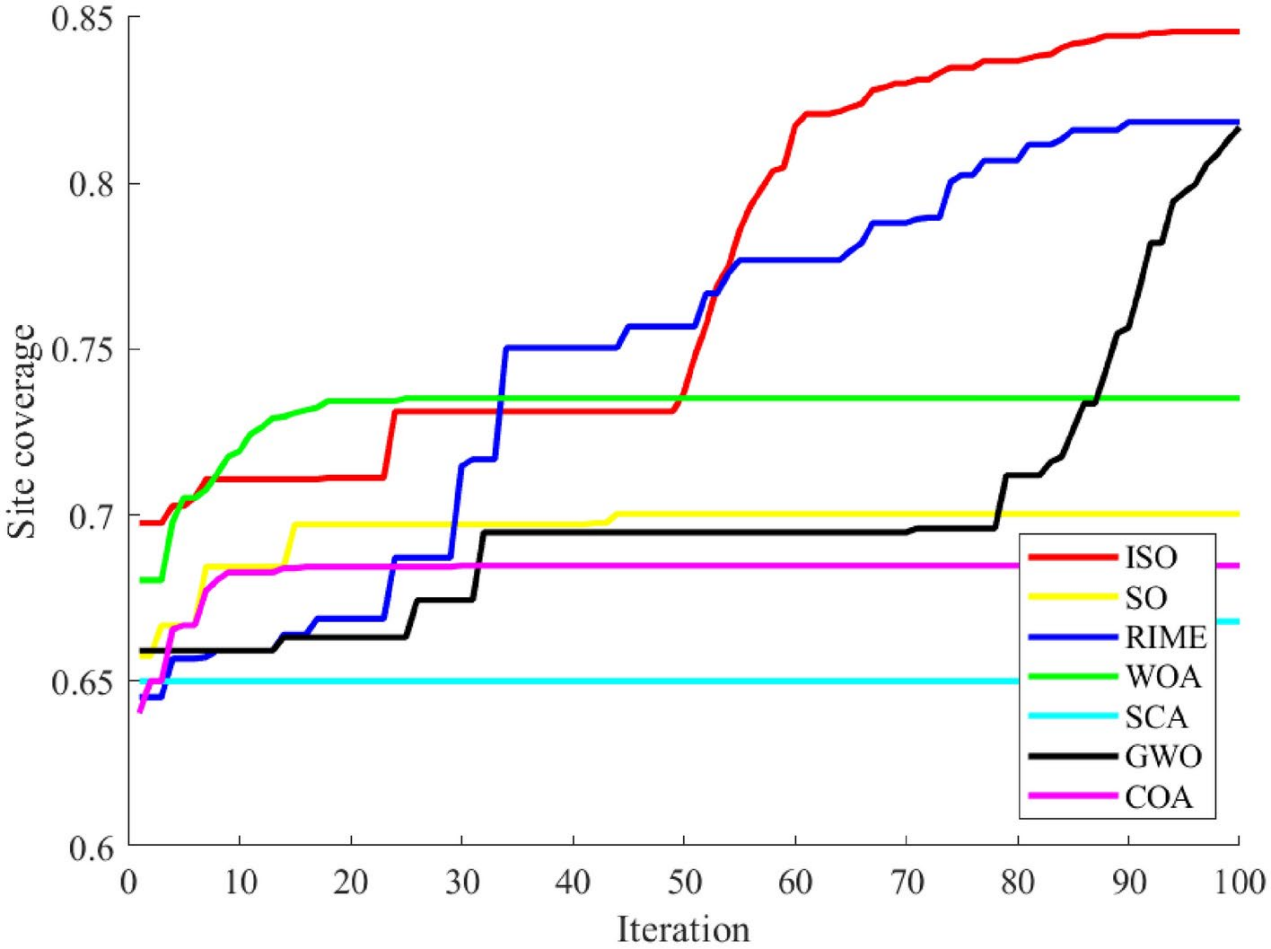
The convergence curves of seven different algorithms applied to the wireless network coverage problem. The figure demonstrates that ISO has the fastest convergence speed and is capable of achieving a larger wireless network coverage area.

Figure 17 presents the visual representation of the coverage area for different algorithms in solving the wireless network coverage problem. Figure 18 displays the convergence curves for the same problem, showing the coverage area for different algorithms. It is evident that ISO achieves the largest network coverage area, outperforming the six competing algorithms.. The experimental results presented in Table 17 compare the performance of various algorithms in optimizing sensor wireless network coverage. The ISO algorithm achieves the highest coverage value of 0.8456, demonstrating its superior ability to maximize sensor coverage. This result highlights ISO’s exceptional efficiency in optimizing network coverage compared to other algorithms. The RIME algorithm follows closely behind with a value of 0.8184, showing competitive performance in terms of coverage efficiency. The GWO algorithm achieves a coverage value of 0.8168, performing slightly better than the WOA algorithm, which achieved a value of 0.6680. The COA algorithm shows a value of 0.6848, indicating moderate performance, while the SO algorithm yields a value of 0.7004, demonstrating a less effective coverage solution. Finally, the SCA algorithm produces the lowest coverage value of 0.7352, indicating its relative inefficiency in optimizing wireless network coverage in this context. These results confirm that the ISO algorithm is the most effective solution for maximizing sensor network coverage, outperforming all other methods tested.

**Table 17:** The final results of the wireless network coverage problem using seven different algorithms.

SCA	GWO	WOA	RIME	COA	SO	ISO
0.7352	0.8168	0.6680	0.8184	0.6848	0.7004	**0.8456**

The table indicates that ISO can achieve the largest wireless network coverage area.

### Pressure vessel design(PVD)

Pressure vessels are widely used where it is required to hold the fluids at high pressure as well as temperature.^80^ They are majorly used in processing plants, nuclear power plants, and oil refining industries. The Pressure Vessel Design(PVD) problem features a structure as shown in Fig. 1. Our design philosophy is to maximize cost-effectiveness while fully meeting practical application needs, by finely adjusting four key parameters to achieve this goal. ? These four crucial optimization dimensions are: vascular thickness ($T_s$), head thickness ($T_h$), inner radius (*R*), and head length (*L*). In order to quantify and optimize the comprehensive impact of these parameters, we have constructed an accurate mathematical model, as shown in equation (54). This model not only reflects the complex relationship between design parameters and performance, but also provides us with a scientific basis for optimizing design, ensuring effective control and reduction of overall costs while meeting usage requirements.

**Fig. 19:**
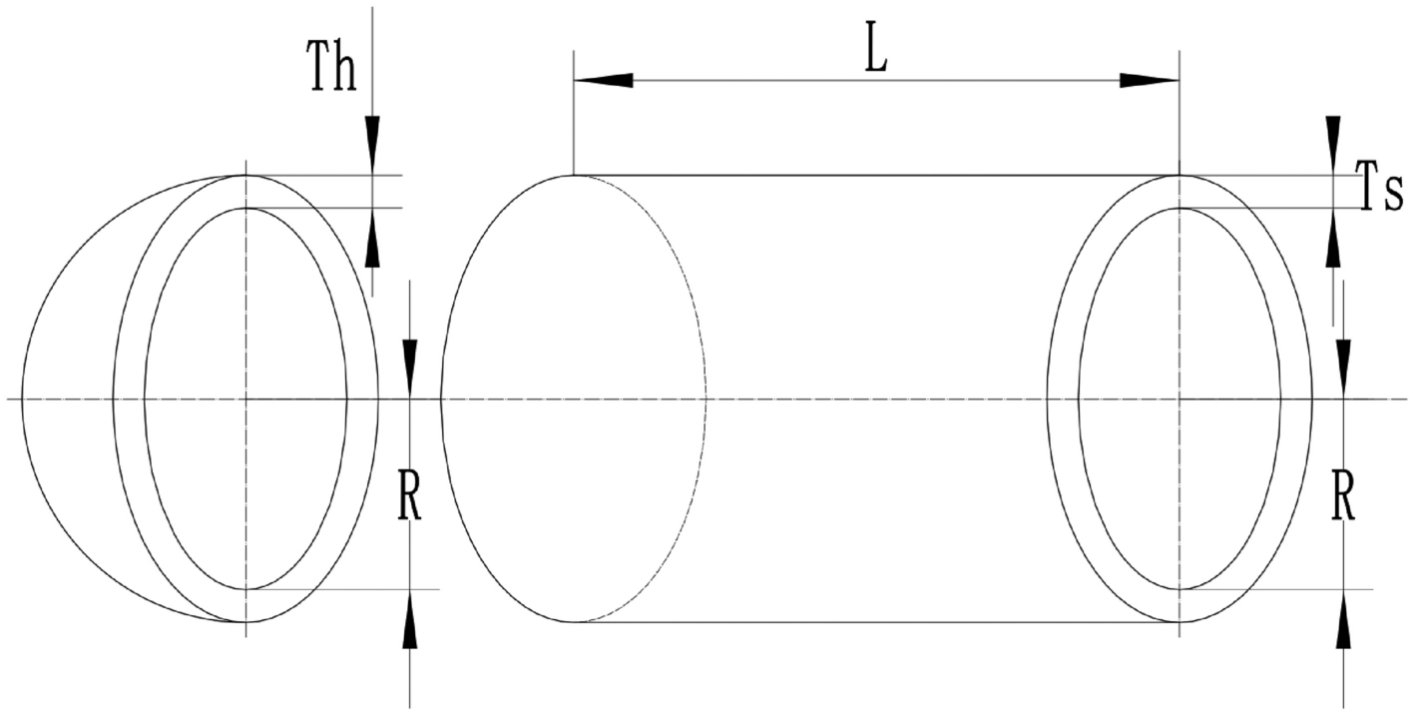
Pressure vessel design diagram, showing the key dimensions including vascular thickness (Ts), head thickness (Th), inner radius (R), and head length (L), which are essential parameters for optimizing the design to meet practical application needs and cost-effectiveness.

**Fig. 20:**
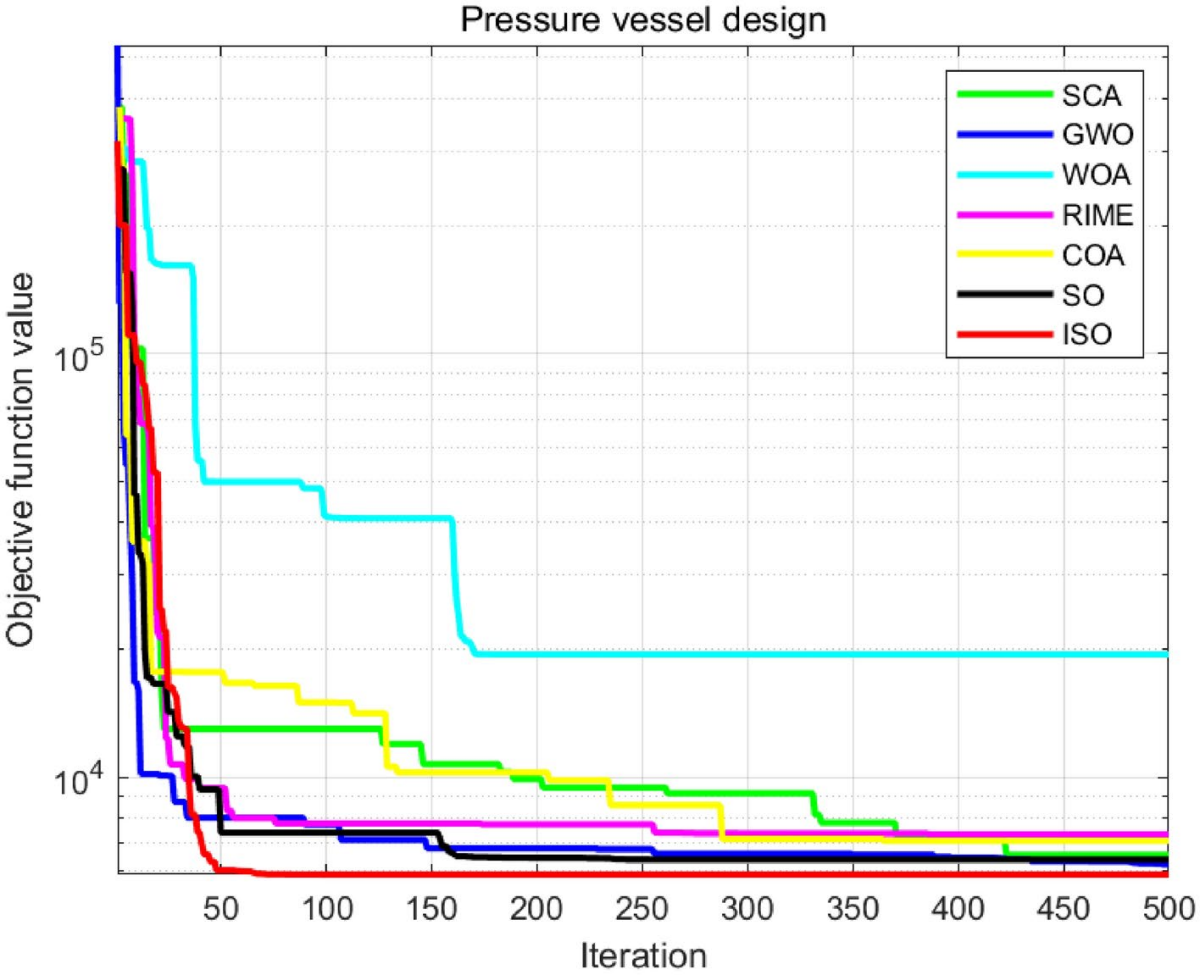
The convergence curves of seven different algorithms for solving the pressure vessel design problem. The ISO algorithm exhibits the fastest convergence speed and is able to obtain the smallest value when solving the pressure vessel design problem.

53 $$\begin{aligned} & \begin{aligned}&\text {Consider} \quad \vec {x} = [x_1, x_2, x_3, x_4] = [T_s, T_h, R, L], \\&\text {Minimize} \quad f(\vec {x}) = 0.6224x_1x_3x_4 + 1.7781x_2x_3^2 + 3.1661x_1^2x_4 + 19.84x_2^2x_3, \\&\text {Subject to} \quad {\left\{ \begin{array}{ll} g_1(\vec {x}) = -x_1 + 0.0193x_3 \le 0, \\ g_2(\vec {x}) = -x_2 + 0.00954x_3 \le 0, \\ g_3(\vec {x}) = -\pi x_3^2x_4 - \frac{4}{3}\pi x_3^3 + 1296000 \le 0, \\ g_4(\vec {x}) = x_4 - 240 \le 0, \end{array}\right. } \end{aligned} \end{aligned}$$

54 $$\begin{aligned} & \text {Parameter range} \quad 0 \le x_1, x_2 \le 99, \quad 10 \le x_3, x_4 \le 200. \end{aligned}$$

Figure 19 provides an illustration of the pressure vessel design problem. Figure 20 presents the convergence curves of different algorithms for solving the pressure vessel design problem. The ISO algorithm exhibits the fastest convergence speed and is able to obtain the smallest value, highlighting the superior performance of ISO in solving this problem. The results presented in Table 18 demonstrate the performance of each algorithm in the pressure vessel design optimization problem, with a clear indication that the ISO algorithm outperforms all other tested algorithms. The ISO algorithm achieves the lowest optimal value of 5885.33, marking it as the most efficient solution for minimizing cost in this design problem. This result places ISO in the leading position among the six competing algorithms, underscoring its strong convergence performance and effectiveness in engineering optimization tasks. The SO algorithm follows closely behind with an optimal value of 5885.33, very near to ISO’s result, but still slightly lagging, indicating that while it performs well, it does not achieve the same level of efficiency as ISO. The GWO and SCA algorithms achieve optimal values of 5903.02 and 7007.05, respectively, both significantly higher than ISO’s result, showing that they are less efficient in finding the optimal solutions for this particular problem. Both WOA and RIME algorithms perform relatively poorly in comparison, with optimal values of 6980.51 and 6482.95, respectively. These higher values suggest that these algorithms are less effective in reaching optimal solutions for the pressure vessel design problem. Overall, the results clearly indicate that the ISO algorithm is the most effective and robust method, consistently providing the best solutions and demonstrating superior convergence properties. Its outstanding performance across various test cases reinforces its reliability for solving the pressure vessel design problem, making ISO the optimal algorithm in this context.

**Table 18:** The detailed results of the seven different algorithms in solving the pressure vessel design problem are presented.

Algorithm	Parameter 1	Parameter 2	Parameter 3	Parameter 4	Optimal value
SCA	0.836050702	0.475055842	43.07312046	178.0706131	6549.74
GWO	0.935854555	0.463953818	48.45401418	111.1205725	6223.10
WOA	1.30641842	2.208910638	65.15999981	10.28122848	19482.87
RIME	1.2601308	0.622765526	65.22794079	10	7328.22
COA	1.02737446	0.465040654	47.24319808	126.283981	7070.00
SO	0.999687962	0.494381086	51.78426909	84.79955776	6384.65
ISO	0.778168641	0.384649163	40.31961872	200	**5885.33**

The first column lists the different algorithms, the next four columns list the relevant parameters, and the last column provides the optimal values.

## Conclusion

This paper introduces the Multi-strategy Improved Snake Optimization Algorithm (ISO), an advanced metaheuristic approach. While the original Snake Optimization Algorithm (SO) has demonstrated effectiveness in various problem domains, it suffers from slow convergence and a tendency to become trapped in local optima. To address these issues, ISO improves the population initialization by employing the Sobol sequence and enhances both convergence speed and global search capability through the integration of elements from the Rime Optimization Algorithm (RIME) and lens imaging-based reverse learning. Additionally, Levy Flight is incorporated to introduce larger random steps, improving the balance between global exploration and local refinement. The algorithm further employs adaptive step-size adjustment, dynamically modifying the step size based on the fitness of the solutions to optimize both exploration and exploitation. A Brownian random walk mechanism is also utilized to introduce random perturbations, which improve local search efficiency and enhance the precision of the solutions. These combined strategies contribute to ISO’s superior robustness, faster convergence, and enhanced global optimization performance. To validate the effectiveness of ISO, population initialization was quantitatively analyzed using metrics such as Star Discrepancy, Average Nearest Neighbor Distance, and Sum of Squared Deviations. Its exploration and exploitation capabilities were evaluated using CEC2017 benchmarks. The results demonstrate that ISO achieves a highly uniform initial population distribution and maintains an optimal balance between exploration and exploitation. Additionally, extensive evaluations were performed using 23 benchmark functions, including CEC 2011 and CEC2017 benchmark functions (across 30, 50, and 100 dimensions), covering unimodal, multimodal, hybrid, and composite functions. The effectiveness of the results was further confirmed through Wilcoxon rank-sum tests, highlighting ISO’s superior performance in terms of convergence speed, stability, and solution quality. ISO’s practical applicability is further evidenced through its successful implementation in complex engineering tasks such as UAV path planning, robotic path planning, wireless network layout optimization, and pressure vessel design. In most cases, ISO consistently outperformed comparative algorithms, demonstrating faster convergence and superior solutions. This reinforces the effectiveness of the integrated improvement strategies. As a result, the ISO algorithm is proven to be a more efficient and robust solution, advancing the field of optimization algorithms and offering a reliable and powerful tool for solving complex real-world problems. Although ISO demonstrates strong performance in most optimization problems, there are still some limitations. On one hand, the current ISO algorithm shows limited improvement on multimodal complex functions with numerous extreme points. On the other hand, the adaptability of the ISO algorithm to discrete variables, time-varying constraints, and Pareto front exploration in multi-objective optimization still requires further research and exploration. Future work will focus on developing self-adaptation mechanisms to reduce reliance on manual parameter tuning, as well as improving ISO’s flexibility in dynamic scheduling, heterogeneous constraint optimization, and multi-objective trade-offs. Furthermore, strengthening the theoretical foundations of the algorithm and expanding its applicability to more complex engineering scenarios will remain central to future research. Cross-domain exploration will also be pursued to fully exploit ISO’s potential and expand its applicability to a broader range of real-world scenarios.

## Data Availability

The relevant data supporting the findings of this study are available from the corresponding author upon reasonable request.
